# Coaxial Dielectric Spectroscopy as an In-Line Process Analytical Technique for Reaction Monitoring

**DOI:** 10.1021/acs.oprd.3c00081

**Published:** 2023-05-30

**Authors:** Desiree M. Dalligos, Michael J. Pilling, Georgios Dimitrakis, Liam T. Ball

**Affiliations:** † Department of Chemical and Environmental Engineering, University of Nottingham, Coates Building, Nottingham NG7 2RD, U.K.; ‡ School of Chemistry, University of Nottingham, Nottingham NG7 2RD, U.K.; § Chemical Development, Pharmaceutical Technology & Development, Operations, AstraZeneca, Macclesfield SK10 2NA, U.K.

**Keywords:** dielectric spectroscopy, reaction monitoring, process analytical technology, multivariate analysis

## Abstract

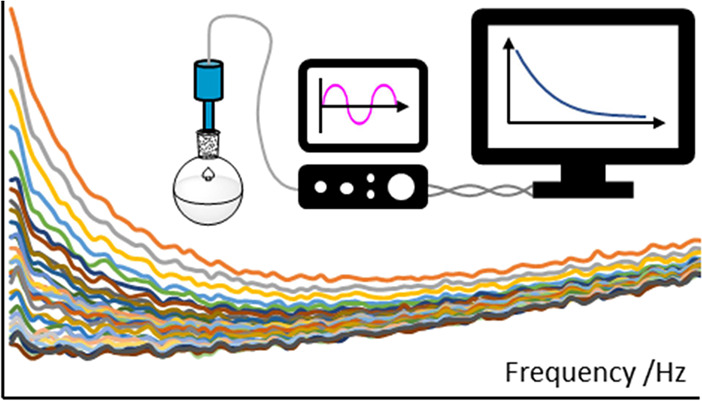

The suitability of broadband dielectric spectroscopy (DS) as a tool for in-line (in situ) reaction monitoring is demonstrated. Using the esterification of 4-nitrophenol as a test-case, we show that multivariate analysis of time-resolved DS data—collected across a wide frequency range with a coaxial dip-probe—allows reaction progress to be measured with both high precision and high accuracy. In addition to the workflows for data collection and analysis, we also establish a convenient method for rapidly assessing the applicability of DS to previously untested reactions or processes. We envisage that, given its orthogonality to other spectroscopic methods, its low cost, and its ease of implementation, DS will be a valuable addition to the process chemist’s analytical toolbox.

## Introduction

Reaction monitoring is essential for the development of new synthetic methodology and robust manufacturing processes, and can provide the quantitative kinetic data required for detailed mechanistic studies. Furthermore, it can provide data that feed directly into process control strategies, thereby facilitating optimization of processes in an industrial setting. Traditional methods of reaction monitoring that involve the collection, preparation, and off-line analysis of discrete samples are often time-consuming and materially wasteful, and do not permit the implementation of advanced process control strategies. Moreover, the very act of sampling may pose technical challenges or safety risks for reactions that employ hazardous reagents or extreme conditions, and may be a source of irreproducibility in sensitive systems.

In contrast, in-line (in situ) monitoring allows reaction progress to be measured in a nondestructive, near real-time fashion, and with reduced risks to process integrity and operator safety (even at extreme conditions^1^). It is therefore unsurprising that in-line monitoring methods have been adopted widely within the academic^2−5^ and industrial^6−9^ communities, and have been recognized formally in the FDA’s PAT framework.^10^ The convenience of modular, probe-based analytical methods has proven especially enabling, despite their potential impact on mixing dynamics^11^ and their susceptibility to fouling.^12^ Following the initial time-investment that may be required to develop an analytical model for an in-line technique—for example by reference to off-line measurements made with an orthogonal method—it can potentially be used to monitor a process ad infinitum; the rapidity with which feedback then becomes available can underpin the implementation of comprehensive process control algorithms.

Although there are many process analytical tools available,^3,4,7,11,13−16^ each has its own advantages and disadvantages (see Supporting Information, Table S1), and there is no single technique that can be applied to every reaction system.^5^ There thus remains a need for new reaction monitoring tools that are potentially complementary to the mainstream analytical methods, which may be able to add value to (in-line) analysis workflows.

Here, we establish dielectric spectroscopy (DS) as a new tool for in-line reaction monitoring and show that—when combined with multivariate data analysis techniques—it can be used to measure reaction profiles that are both accurate and precise. In addition, we also report a simple test for deciding rapidly on the suitability of DS as an analytical tool for any new process being studied.

**Figure 1:**
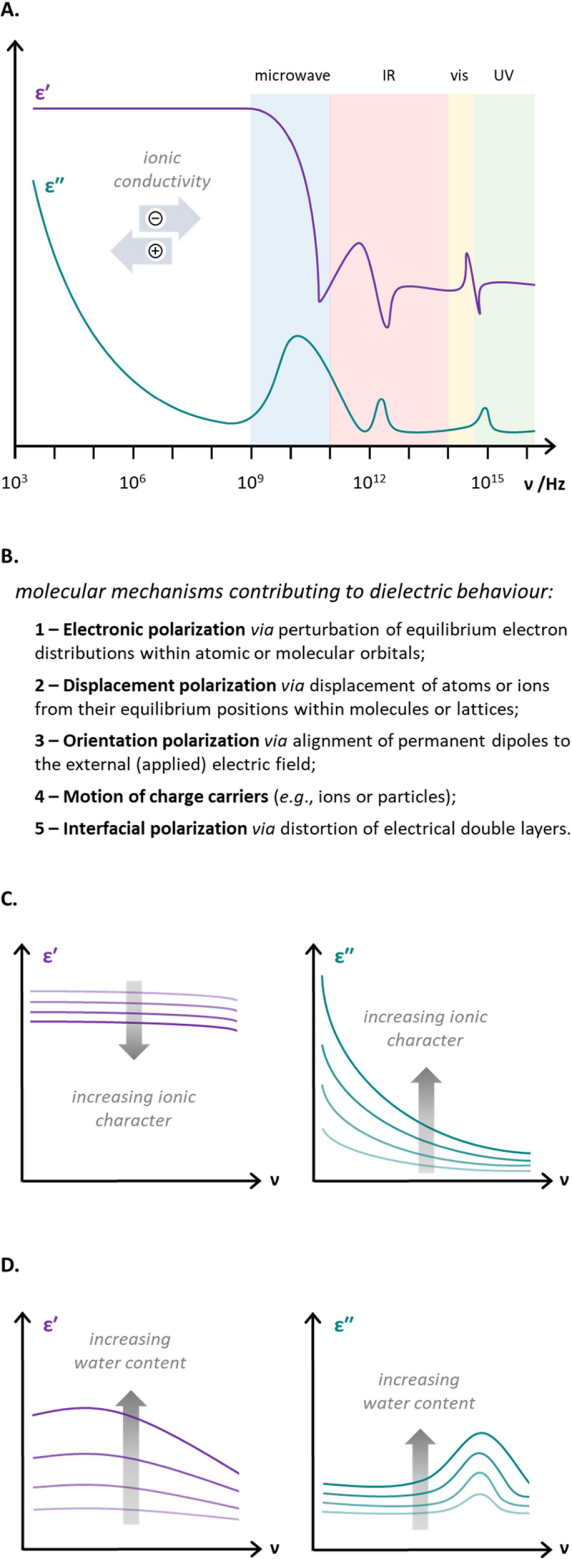
Key features of DS. (a) Overview of the dielectric spectrum; (b) key molecular mechanisms that contribute to the observed dielectric properties of a sample; (c) schematic representation of the changing dielectric properties of a sample as it becomes more ionic (assuming no accompanying changes in dipolar relaxation); (d) schematic representation of the changing dielectric properties of a nonpolar sample as water is added.

DS measures complex relative dielectric permittivity (ε_r_*) as a function of frequency (eq 1 and Figure 1A).^17,18^ The complex relative dielectric permittivity (ε_r_*) is composed of the dielectric constant (ε′) and the dielectric loss (ε″), which are respectively the ease with which a sample polarizes, and how easily that polarization energy is converted into heat. Of the many molecular mechanisms that can contribute to the observed dielectric properties of a sample,^19^ those that are most likely to change during a chemical reaction—and which are therefore most relevant to monitoring reaction progress—are listed in Figure 1B. It is important to note here that both the reorientation of permanent dipoles and the mobility of charge carriers are very sensitive to the viscosity of the medium, and hence to temperature.
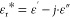
1where ε_r_* = complex relative dielectric permittivity; ε′ = dielectric constant; *j* = √(−1); ε″ = dielectric loss.

While the spectral contribution of individual species within a mixture may be resolved in some cases,^20,21^ DS more typically measures the net dielectric properties of the bulk sample. In contrast to the spectra of pure analytes,^22^ detailed interpretation or a priori prediction of the dielectric spectrum of a mixture is therefore rarely possible. Notwithstanding, the curvature of a dielectric spectrum may still provide valuable insight into the chemical composition of a sample and the nature of any changes it undergoes. For example, the trend illustrated in Figure 1C arises due to the particularly strong contribution that ionic species make to dielectric loss (ε″) at low frequencies and would be consistent with salt formation occurring during a reaction. Similarly, characteristic spectral changes accompany a change in the concentration of polar neutral species (Figure 1D), such as the elimination of water during a dehydrative condensation.

Because DS often provides insight only into the composition of the whole mixture, its use in reaction monitoring will likely be sensitive to the presence of impurities and parallel processes (e.g., side-reactions, crystallization events, etc.). In this way, it is similar to established ‘integral’ methods^19^ including reaction calorimetry^23^ and measurements of physico-chemical properties (e.g., density,^24^ conductivity,^25^ and refractive index^26,27^). Furthermore, as noted above, dielectric property measurements can also be very sensitive to temperature.^19^ Given the number of intimately linked factors contributing to the dielectric properties of a sample (Figure 1B), it is not straightforward to predict how sensitive a single measurement will be to temperature, or whether the degree of sensitivity changes as a function of composition (e.g., as a reaction progresses). A process-by-process analysis is thus required to understand the influence of temperature on DS measurements and hence to determine how rigorously this process parameter must be accounted for^28^ or controlled.

**Figure 2:**
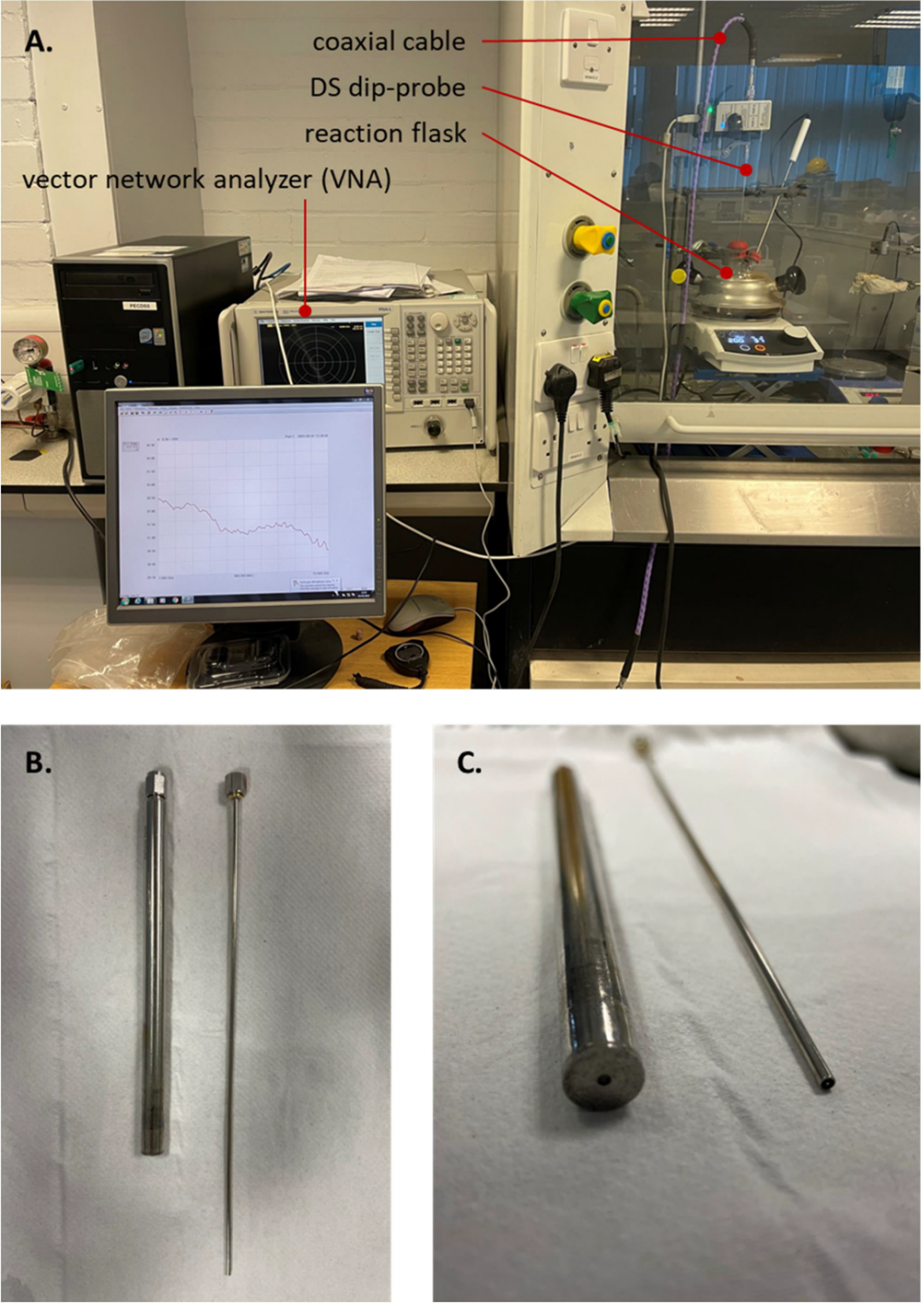
(a) Typical experimental setup for coaxial DS; (b and c) close-up of high-performance (left) and slim (right) probes.

Since much of organic synthesis is predicated on making changes to polar functional groups,^29−35^ we hypothesized that DS might be an appropriate spectroscopic method for measuring reaction progress. DS boasts several features that are well-suited to in-line reaction monitoring in general (Table 1, entries 1, 2 and 4–6; Figure 2), as well as more specifically to plant-scale operations (entries 3 and 7).^36,37^

**Table 1:** Practical Considerations

entry	attributes of DS beneficial to in-line reaction monitoring
1	low cost,^a^ operational simplicity, and low maintenance requirements of equipment
2	commercial availability of broad-frequency coaxial dip-probes (Figure 2), allowing for integration with preexisting chemistry workflows
3	commercial availability of coaxial cables of sufficient length^b^ for insertion into manufacturing-scale vessels
4	high temporal resolution, potentially allowing for measurements on a subsecond timescale^c^
5	applicability to measurements in flow^38^
6	nondestructive in nature
7	low power (<1 mW) emitted from the vector network analyzer, such that a fouled probe will not cause localized heating that could otherwise be a source of ignition

a Typical prices for research-quality (process-agnostic) instruments: vector network analyzer (VNA), £6–£20k; coaxial dip-probe, £3–£4k; coaxial cable, £1–£2k. Instrumentation dedicated to monitoring a specific, well-characterized process: nano-VNA, £200–£500; coaxial dip-probe, £1k; coaxial cable, £1k.

b For example, 4 m coaxial cables are available, comparable to fiber optics used in well-established FT-IR and UV–vis process monitoring technologies. Alternatively, the VNA and coaxial probe can be connected wirelessly.

c For example, the instrument used in this study sweeps the entire frequency range in 1.459 ms.

Although DS is used widely in the analysis of material properties^17,39,40^ and biomass concentration,^41−44^ its application to reaction monitoring is—to the best of our knowledge—limited to polymerizations.^45−50^ These typically represent relatively simple systems in which neat monomers are converted into very physically and chemically distinct products, with no byproducts or solvents that could convolute DS measurements or confound univariate (single-frequency) data analysis. The applicability and robustness of DS as a tool for monitoring the progress of more complex, small-molecule processes thus remain to be established.

## Results and Discussion

**Figure 3:**
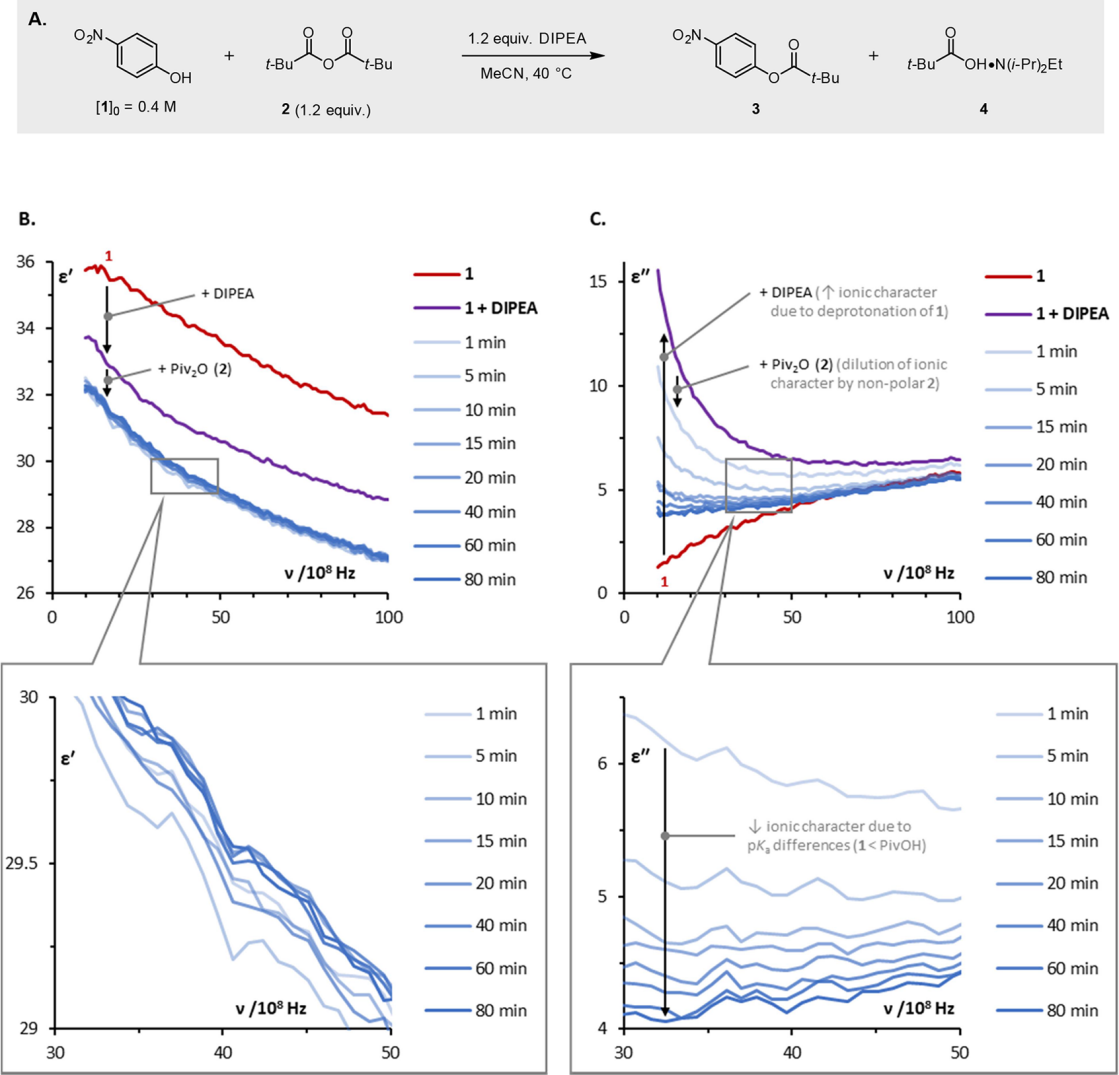
(a) Esterification of 4-nitrophenol **1** with pivalic anhydride **2** (≥95% yield at 80 min, as determined by ^1^H NMR spectroscopic analysis vs 1,3,5-trimethoxybenzene); (b) representative, time-resolved plots of dielectric constant (ε′) vs frequency; (c) representative, time-resolved plots of dielectric loss (ε″) vs frequency. Spectra are raw data from a single measurement and show noise which originates from the residual errors after the open-ended coaxial line calibration. Lines between implicit data points (every 90 MHz) are intended as a guide for the eye; expansions are selected to illustrate spectral resolution only. DIPEA = diisopropylethylamine; Piv = pivaloyl (trimethylacetyl).

To investigate the suitability of DS for in-line reaction monitoring, we studied the esterification of 4-nitrophenol **1** with pivalic anhydride **2** in the presence of DIPEA (Figure 3A; see the Experimental Section for details). We anticipated that the formation of a nominally ionic co-product—the trialkylammonium pivalate salt **4**—from neutral starting materials would result in a significant change in dielectric properties between reaction initiation and completion, and therefore that it would be suitable for testing the concept. Furthermore, this reaction can be followed conveniently by orthogonal analytical techniques (e.g., ^1^H NMR and FT-IR spectroscopy methods), allowing for both independent validation of DS results and the production of quantitative models.

Addition of DIPEA to a thermally equilibrated solution of 4-nitrophenol **1** in MeCN led to significant changes in both the dielectric constant and the dielectric loss across a broad frequency range (1–10 GHz; compare the red and purple curves in Figure 3B,C). The observed drop in the dielectric constant is consistent with the addition of a relatively nonpolar species (DIPEA) to a polar medium (MeCN). The change in dielectric loss is perhaps more revealing: the shape of the initial spectrum (red curve) in the low-frequency region is typical of a polar neutral mixture (i.e., 4-nitrophenol **1** in MeCN), whereas addition of DIPEA leads to a spectrum characteristic of ionic speciation (cf. Figure 1C). This spectral change suggests that 4-nitrophenol **1** is at least partially deprotonated by the base, consistent with their relative acidity and basicity (in MeCN at 298 K: p*K*_aH_(DIPEA), 18.2 ± 0.9;^51^ p*K*_a_(4-nitrophenol), 21.3 ± 0.2^52^).

The dielectric constant decreases further when the esterification reaction is initiated by addition of pivalic anhydride **2** (compare the purple and light blue curves in Figure 3B), but only minimal changes are subsequently observed over the course of the reaction. In contrast, the dielectric loss exhibits low-frequency dispersion, which falls upon initiation of the reaction (compare the purple and light blue curves in Figure 3C) and then continues to fall significantly as the reaction progresses. The changes observed in dielectric loss suggest that the reaction mixture becomes less ionic in character as the esterification proceeds, consistent with the higher p*K*_a_ of carboxylic acids relative to 4-nitrophenol **1** (p*K*_a_ in MeCN at 298 K: AcOH, 23.5;^53^ 4-nitrophenol, 21.3 ± 0.2^52^).

**Figure 4:**
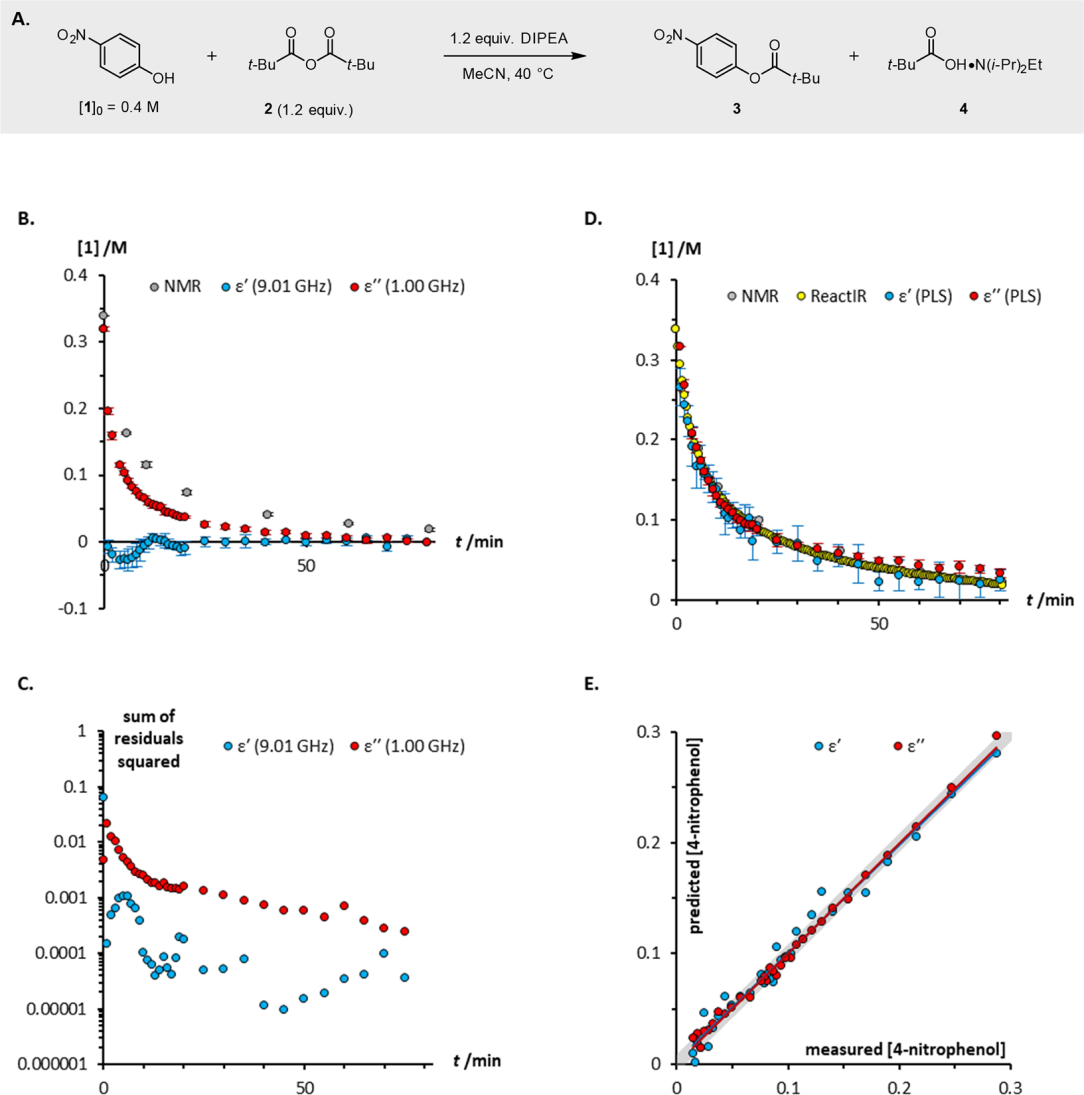
(a) Esterification of 4-nitrophenol **1** with pivalic anhydride **2**; (b) reaction profiles measured using ^1^H NMR spectroscopy, and calculated from dielectric constant and dielectric loss data at single frequencies; (c) error in the fit of the reaction profiles calculated using DS to the profile measured using ^1^H NMR spectroscopy; (d) reaction profiles measured using ^1^H NMR and FT-IR spectroscopy, and predicted from dielectric constant and dielectric loss data using partial least squares (PLS) multivariate analysis; (e) correlation of the measured and predicted concentrations of 4-nitrophenol **1** (for the dielectric constant model: RMSEC = 0.0101, RMSECV = 0.0202, RMSEP average = 2.00 ± 1.20 stdv; for the dielectric loss model: RMSEC = 0.00489, RMSECV = 0.00518, RMSEP average = 0.0493 ± 0.0246 stdv). Data points in Figure 4B,D are averages of five independent repetitions; error bars represent the standard deviation in each time-point. Red and blue lines in Figure 4E are the least squares lines of best fit; the gray line is ‘predicted = measured’ (*x* = *y*).

We initially considered a simple univariate (single frequency) analysis of the DS data, analogous to the approach employed previously in the monitoring of caprolactone polymerization.^45−47^ Given the spectral width of the individual DS measurements, a statistically informed approach to frequency selection was employed. Thus, the concentration-time profile generated at each frequency of the DS data was compared to a quantitative model prepared from ^1^H NMR spectroscopic data. The frequencies that returned the lowest sum of residual squares—9.01 GHz and 1.00 GHz for dielectric constant and dielectric loss, respectively—were selected for further assessment (Figure 4B,C). Although the reaction profile generated from dielectric loss has better accuracy compared to that derived from the dielectric constant, neither model gives an acceptable correlation to the NMR spectroscopic data.

Figure 4B,C provides a clear illustration of the limitations associated with univariate analysis: although it provides convenience, a significant amount of spectral data that could otherwise be used to accurately fit complex datasets is sacrificed. To address this shortcoming, multivariate data analysis was applied to the dielectric data. A prediction model was created using PLS regression by using a training data set and the model was validated using independent data sets. As shown in Figure 4D,E, the resulting model gives both excellent accuracy and precision and allows for reliable quantification of reaction progress to approximately 95% conversion. We note here that, although we performed PLS regression using a proprietary software suite within MATLAB, the same functions are available within other common software (e.g., using proprietary plug-ins for Microsoft Excel, the nonproprietary Sci-kit learn module in Python,^54^ or the nonproprietary pls package in R^55^).

### Robustness

**Figure 5:**
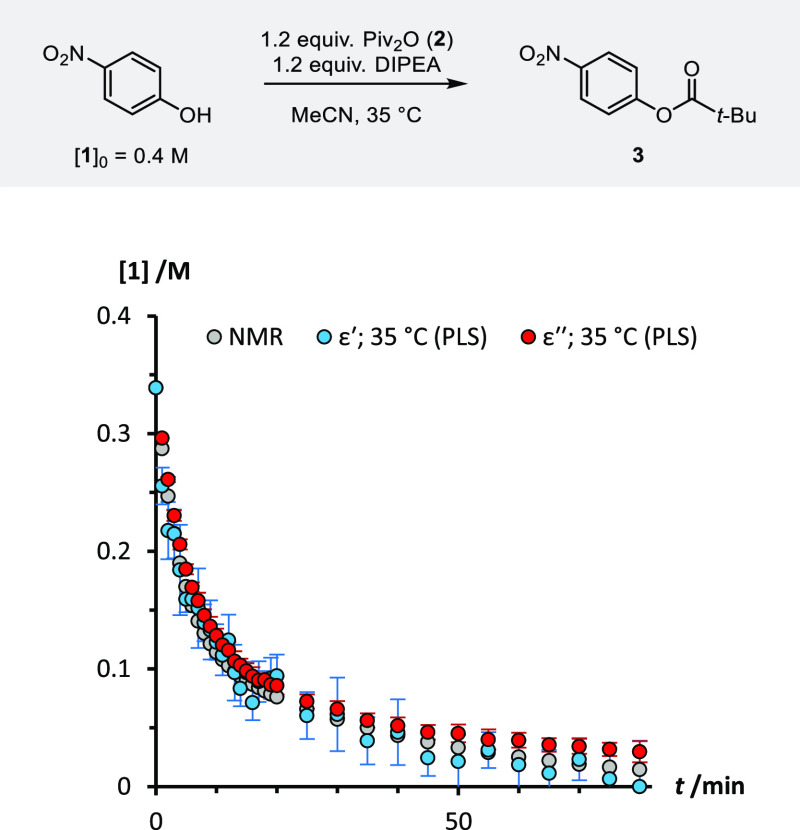
Reaction profiles for the esterification of 4-nitrophenol **1** at 35 °C measured using ^1^H NMR spectroscopy, and predicted from dielectric constant and dielectric loss data using the PLS model trained on DS data collected from reactions performed at 40 °C. Data points are averages of five independent repetitions; error bars represent the standard deviation in each time-point.

To investigate the robustness of the PLS model, and of DS as an in-line process analytical tool, the esterification of 4-nitrophenol **1** was repeated at 35 °C. When the resulting five new data sets were tested on the 40 °C PLS model, excellent correlation was obtained against profiles generated from both NMR and IR spectroscopy methods (Figure 5). The PLS model developed for this reaction can therefore still be applied with good accuracy over a 5 °C variation in temperature. However, as discussed in the Introduction, the temperature sensitivity of DS varies on a sample-by-sample basis and should therefore be assessed for each new process under investigation.

### Sensitivity

An important application of PAT is in the determination of reaction endpoint,^56−58^ which necessarily requires the detection of small changes in the concentration of low abundance (limiting) reagents. While an appreciation of the limit of detection of DS would be valuable in this regard, it is hard to predict by simple inspection of a chemical scheme. Indeed, due to the integral nature of DS, any predictions or generalizations would need to account for a large number of polarization mechanisms (Figure 1B) across all components of the mixture, and the modification of these contributions as a result of intermolecular interactions. Furthermore, in addition to spectroscopic considerations, the practical limit of detection offered by DS will depend on how well the instrument is calibrated.

To expedite the uptake of DS as a tool for reaction monitoring, we therefore sought to develop a convenient and reliable experimental method that could determine whether DS is applicable to a previously untested reaction. Crucially, this triage process should account for both spectroscopic considerations and the quality of instrument calibration, while avoiding the need to collect the full datasets required for PLS model generation (i.e., measuring multiple reaction profiles by both DS and an orthogonal technique, such as quantitative NMR or calibrated FT-IR/HPLC), by confirming that:

(1) there is an appreciable change in dielectric properties over the course of the reaction, and
(2) this change is above the limit of detection of the technique.

A straightforward visualization of suitability is provided by making three repeat DS measurements at the beginning and at the end of the candidate reaction, but without having to measure a full reaction time-course, perform independent repetitions, or use orthogonal monitoring techniques. We thus define the ‘sensitivity’ at each frequency point measured (*S*_ν_) as the average difference between the initial and final measurements, compared to the average standard deviations of the individual measurements (eq 2). The ‘error term’ represented by the standard deviations in eq 2 accounts for the quality of instrument calibration, and therefore ensures that a practical—rather than a theoretical—sensitivity is determined.
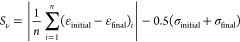
2where *S*_ν_ = sensitivity at frequency ν; *n* = number of repeat measurements (typically *n* = 3); ε_initial_ = DS measurement at frequency ν, made at the start of the reaction; ε_final_ = DS measurement at frequency ν, made at the end of the reaction; σ_initial_ = standard deviation in all the DS measurements at frequency ν, made at the start of the reaction; σ_final_ = standard deviation in all the DS measurements at frequency ν, made at the end of the reaction.

**Figure 6:**
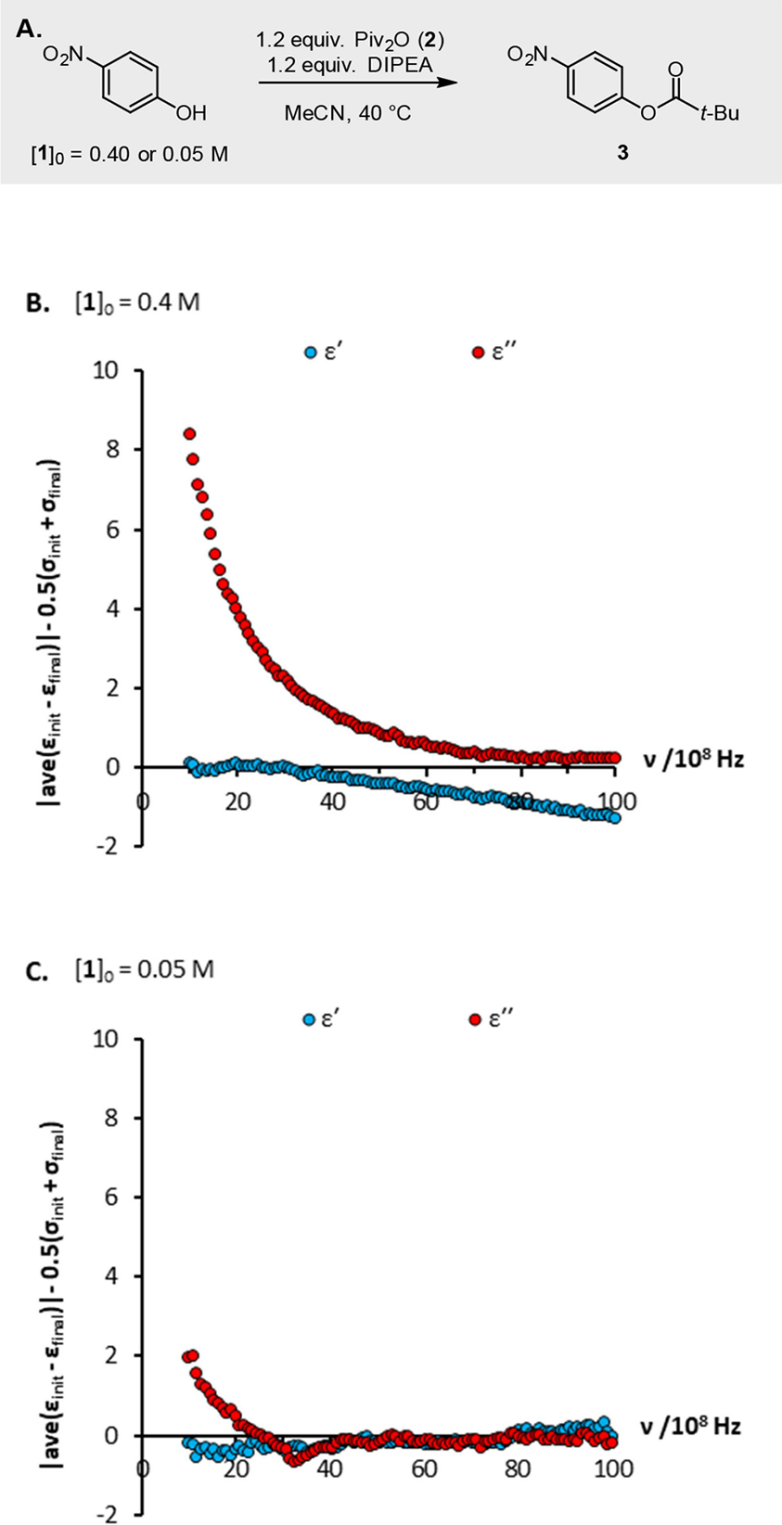
Suitability of a reaction for monitoring by DS can be determined visually by comparison of the net spectral change observed over the course of a reaction to the errors inherent in the measurements (eq 2). (a) Esterification of 4-nitrophenol **1** with pivalic anhydride **2**; analysis demonstrating that (b) at [**1**]_0_ = 0.4 M, the esterification can be monitored by dielectric loss but not dielectric constant (cf. Figure 4D), whereas (c) at [**1**]_0_ = 0.05 M, the esterification cannot be monitored by either the dielectric constant or dielectric loss.

The resulting ‘sensitivity’ values (*S*_ν_) are plotted against frequency (ν/Hz); if the resulting curve lies above the *x*-axis, then the reaction likely provides sufficient spectral change for monitoring by DS to be viable. However, if the curve largely lies close to or below the *x*-axis, then the reaction likely cannot be monitored accurately with DS. A posteriori application of this test to the esterification of 4-nitrophenol **1** (Figure 6A; [**1**]_0_ = 0.4 M) indicates that the dielectric constant gives insufficient change (blue curve; Figure 6B) and is therefore poorly suited to monitoring this specific reaction. In contrast, the dielectric loss shows a very clear and statistically significant spectral change (red curve; Figure 6B) and—as borne out in experiment (Figure 4D)—is predicted to be suitable for monitoring this reaction.

The same approach can be employed to estimate rapidly the limit of detection of DS and hence determine the lowest initial concentration of a given reaction that can be monitored reliably. Thus, by making repeat DS measurements at different serial dilutions of an initial and a final reaction mixture, we determine that for the esterification shown in Figure 6A, there is no longer sufficient spectral change in the dielectric loss to allow the reaction to be monitored reliably at an initial nitrophenol concentration of 0.05 M (Figure 6C).

**Figure 7:**
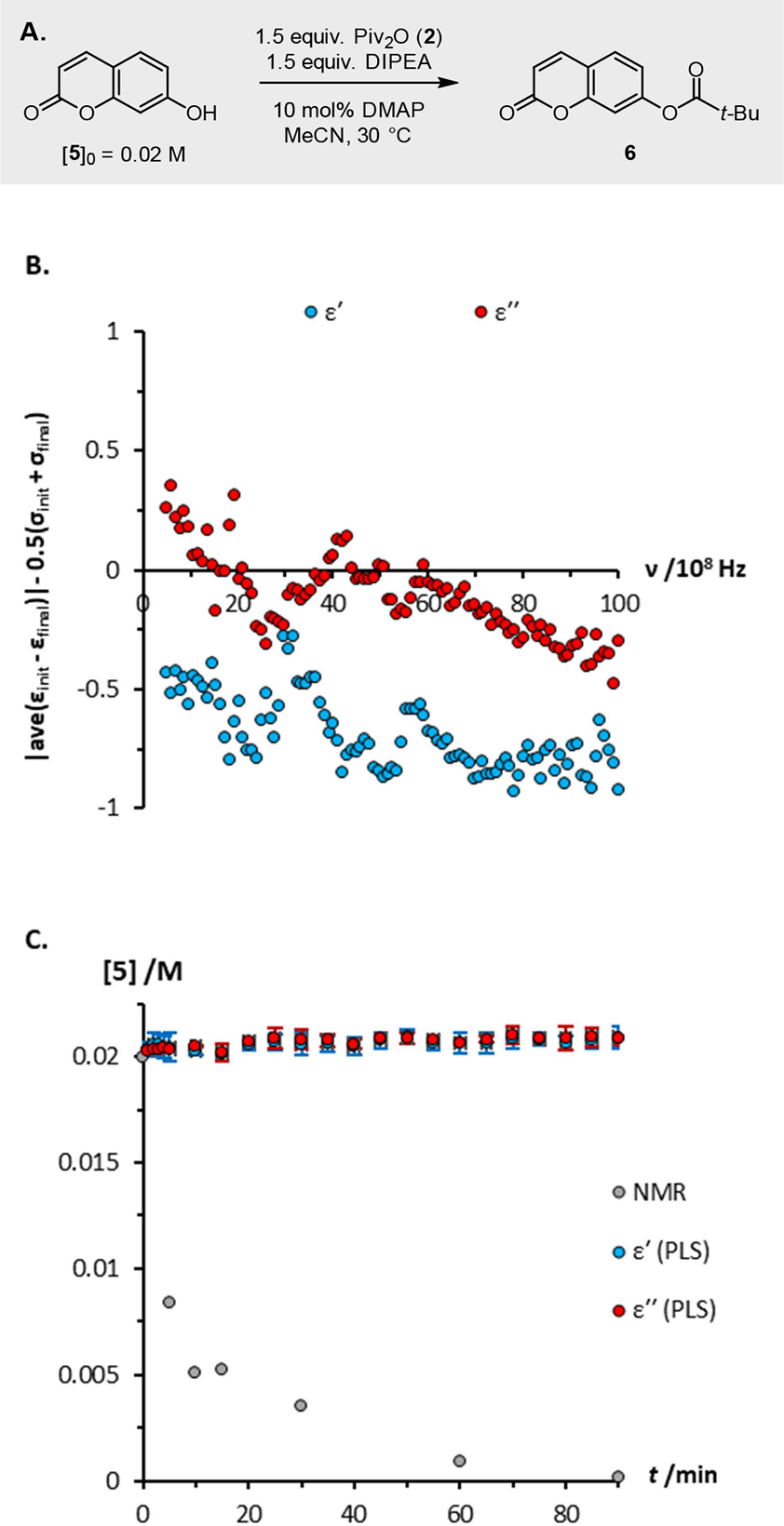
(a) Esterification of umbelliferone **5** with pivalic anhydride **2**; (b) analysis predicting that the reaction cannot be monitored reliably using changes in dielectric constant or dielectric loss; (c) reaction profiles measured using ^1^H NMR, and predicted from dielectric constant and dielectric loss data using PLS multivariate analysis. Error bars in Figure 7C represent the standard deviation in each time-point. DMAP = *N*,*N*-dimethylaminopyridine.

The utility of this ‘reaction suitability’ test protocol is illustrated by its application to processes that had not been studied previously with DS. For the esterification of umbelliferone **5** with pivalic anhydride **2** (Figure 7A), the test protocol predicts that neither the dielectric constant nor the dielectric loss change sufficiently over the course of the reaction for it to be monitored reliably by DS (Figure 7B). To confirm these predictions, the esterification was monitored by both ^1^H NMR spectroscopy and DS, and a PLS model was prepared. As shown in Figure 7C, the concentration-time profiles predicted using the dielectric data are in no way representative of the actual reaction progress.

**Figure 8:**
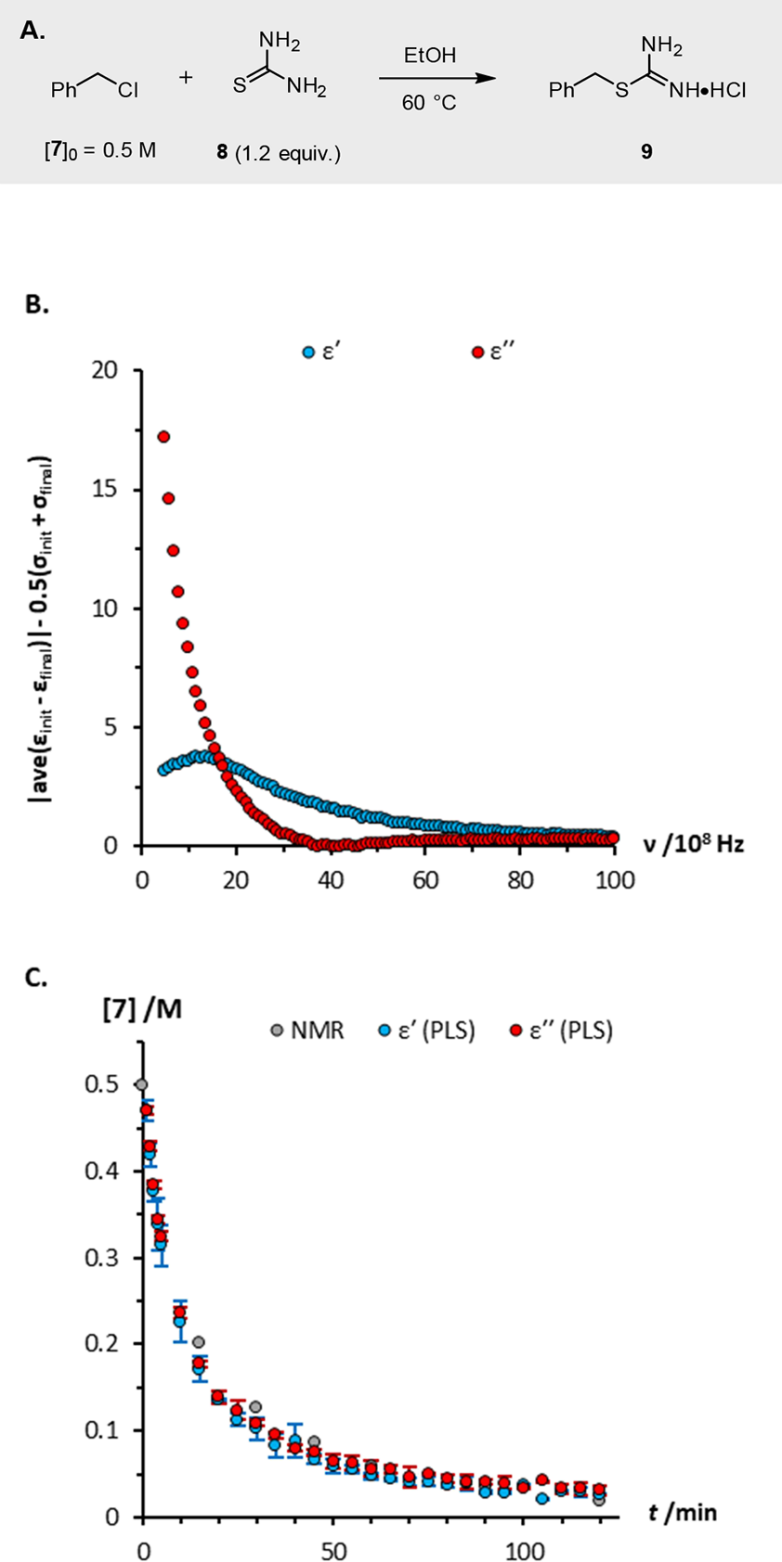
(a) *S*-Alkylation of thiourea **8** with benzyl chloride **7**; (b) analysis predicting that the reaction can be monitored reliably using changes in either dielectric constant or dielectric loss; (c) reaction profiles measured using ^1^H NMR, and predicted from dielectric constant and dielectric loss data using PLS multivariate analysis. Error bars in Figure 8C represent the standard deviation in each time-point.

Finally, we studied the synthesis of *S*-benzyl isothiouronium chloride **9** via alkylation of thiourea **8** with benzyl chloride **7** in ethanol (Figure 8A). While this reaction converts neutral starting materials to an ionic product—and could therefore be expected to result in a significant change in dielectric properties—the highly polar solvent is likely to reduce the sensitivity of the measurements. Given this potential juxtaposition, the ‘reaction suitability’ test protocol was employed to assess the appropriateness of DS as a monitoring technique (Figure 8B).

As shown in Figure 8B, the *S*-benzylation of thiourea **8** is accompanied by major changes in both the dielectric constant and the dielectric loss of the reaction mixture. These spectral changes are significant relative to the error in the measurements, indicating that the reaction is appropriate for monitoring by DS. Indeed, the reaction profiles predicted from the dielectric constant and the dielectric loss both replicate accurately the profile measured using ^1^H NMR spectroscopy (Figure 8C), thereby validating both the ‘reaction suitability’ test protocol and the utility of DS as a tool for in-line reaction monitoring.

## Conclusions

We have demonstrated that DS can be employed as a process analytical technique for the in-line monitoring of chemical reactions. By applying DS to three distinct systems, we establish (a) the importance of using multivariate data analysis in order to obtain precise and accurate reaction profiles, (b) the method’s sensitivity to temperature fluctuations, and (c) a convenient method for rapidly assessing the applicability of DS to a new reactions or processes. Thus, given the low cost and robustness of the requisite hardware, and its complementarity to more widespread spectroscopic techniques, we anticipate that DS will prove a useful addition to the reaction monitoring toolbox.

## Experimental Section

### General Methods

#### Synthetic Chemistry

Reagents and solvents were used as supplied by commercial vendors. ^1^H NMR spectra were obtained at 25 °C on a Bruker Avance 400 spectrometer. Chemical shifts (δ) are given in ppm relative to tetramethylsilane and are referenced to residual solvent peaks for DMSO-*d*_6_ (2.50/39.5 ppm) or CDCl_3_ (7.26/77.2 ppm). Multiplicities are reported as singlet (s), doublet (d), triplet (t), multiplet (m), broad (br), app (apparent) or combinations thereof. Coupling constants, *J*, are reported in Hz. High-resolution mass spectrometry (HRMS) was performed using a Bruker MicroTOF spectrometer with an electrospray ionization (ESI) ion source. Infrared spectra (IR) were recorded on a Bruker Alpha platinum-ATR with diamond window. Melting points were measured on a Stuart SMP20 melting point apparatus in open capillaries.

#### In-Line Analytical Measurements

Dielectric measurements were made using an Agilent 85070E open-ended coaxial slim probe or high performance probe connected to Keysight N5232A PNA-L Vector Network Analyzer (VNA) with a frequency range of 0.5–20 GHz. Three standards were used for calibration: open (air), short (calibration short tool), and a reference liquid (deionized water). 101 data points were acquired for each measurement. In-line FT-IR spectra were obtained using a Mettler Toledo ReactIR iC10 equipped with a liquid nitrogen-cooled MCT detector and a 6 mm AgX Fiber DiComp probe at 8 cm^–1^ resolution. Spectra were recorded every 30 s.

#### Data Analysis

Calculations and data processing were performed using Microsoft Excel, MATLAB and PLS_Toolbox (Eigenvector Research, Inc.). Kinetic simulations were performed using COPASI.

### Typical Procedures

#### Esterification of 4-Nitrophenol **1** with Pivalic Anhydride **2**

4-Nitrophenol **1** (1.67 g, 12.0 mmol) and acetonitrile (30 mL) were added to a two-necked round bottomed flask (50 mL) equipped with a cross-shaped magnetic stirrer bar. The DS probe was calibrated, and the probe and a digital thermocouple were immersed in the solution through a pierced rubber septum. The solution was stirred (200 rpm) at 40 °C and allowed to thermally equilibrate for 30 min, before a dielectric measurement was made (1–10 GHz). *N*,*N*-Diisopropylethylamine (2.50 mL, 14.4 mmol) was added, and the mixture was again analyzed by DS. Pivalic anhydride **2** (2.90 mL, 14.4 mmol) was then added to initiate the esterification, and the reaction was analyzed by DS every min until 20 min, then every 5 min until 80 min. Aliquots of the reaction mixture were removed for off-line analysis by ^1^H NMR spectroscopy at 5.5, 10.5, 20.5, 40.5, 60.5, and 80.5 min. Each sample was quenched immediately with aqueous HCl (1 M) and extracted with Et_2_O, the organic layer was separated, and the solvent was evaporated in vacuo. CDCl_3_ was added to the residue, and the resulting sample was passed through a pad of MgSO_4_ in a Pasteur pipette prior to analysis by ^1^H NMR spectroscopy. At the end of the reaction, the remaining reaction mixture was quenched with aqueous HCl (1 M), extracted with Et_2_O (3 × 20 mL), the organic layers were combined, washed with saturated aqueous NaHCO_3_ and brine, dried over MgSO_4_, and concentrated in vacuo to give 4-nitrophenyl pivalate **3** (2.14 g, 9.60 mmol, 80%) as a yellow solid (m.p. 95–97 °C, lit.^59^ 95–96 °C). ^1^H NMR (400 MHz, CDCl_3_): δ 8.27 (d, *J* = 8.0 Hz, 2H), 7.25 (d, *J* = 8.0 Hz, 2H), 1.38 (s, 9H). ^13^C{^1^H} NMR (100 MHz, CDCl_3_): δ 176.3, 156.1, 145.4, 125.3, 122.5, 39.5, 27.1. υ_max_ (ATR/cm^–1^): 3117, 3086, 2974, 2940, 2876, 1747, 1520, 1480, 1345, 1206, 1092, 1026, 895, 739, 492. HRMS (ESI^+^) *m*/*z*: calcd for C_11_H_13_NO_4_ + Na^+^: 246.0737 [M + Na]^+^; found 246.0743. Characterization data were consistent with literature values.^59^

#### Esterification of Umbelliferone **5** with Pivalic Anhydride **2**

Umbelliferone **5** (81.1 mg, 0.50 mmol), DMAP (6.11 mg, 0.050 mmol), and acetonitrile (25 mL) were added to a two-necked round bottomed flask (50 mL) equipped with a cross-shaped magnetic stirrer bar. The DS probe was calibrated, and the probe and a digital thermocouple were immersed in the solution through a pierced rubber septum. The solution was stirred (200 rpm) at 30 °C and allowed to thermally equilibrate for 30 min, before a dielectric measurement was made (1–10 GHz). *N*,*N*-Diisopropylethylamine (131 μL, 0.75 mmol) was added, and the mixture was again analyzed by DS. Pivalic anhydride **2** (152 μL, 0.75 mmol) was then added to initiate the esterification, and the reaction was analyzed by DS every min until 5 min, and then every 5 min until 90 min. Aliquots of the reaction mixture were removed for off-line analysis by ^1^H NMR spectroscopy at 0.5, 5.5, 10.5, 15.5, 30.5, 60.5, and 90.5 min. Each sample was quenched immediately with aqueous HCl (1 M), extracted with EtOAc, the organic layer was separated and the solvent evaporated in vacuo. CDCl_3_ was added to the residue, and the resulting sample was passed through a pad of MgSO_4_ in a Pasteur pipette prior to analysis by ^1^H NMR spectroscopy. At the end of the reaction, the remaining reaction mixture was quenched with aqueous HCl (1 M), extracted with EtOAc (3 × 20 mL), the organic layers were combined, dried over MgSO_4_, and concentrated in vacuo to give umbelliferyl pivalate **6** (95.8 mg, 0.39 mmol, 78%) as a colorless solid (m.p. 140–143 °C, lit.^60^ 136–138 °C). ^1^H NMR (400 MHz, CDCl_3_): δ 7.69 (d, *J* = 9.5 Hz, 1H), 7.48 (d, *J* = 8.4 Hz, 1H), 7.07 (d, *J* = 2.2 Hz, 1H), 7.01 (dd, *J* = 8.4, 2.2 Hz, 1H), 6.39 (d, *J* = 9.5 Hz, 1H), 1.37 (s, 9H). ^13^C{^1^H} NMR (100 MHz, CDCl_3_): δ 176.6, 160.5, 154.9, 153.9, 143.0, 128.6, 118.5, 116.6, 116.1, 110.5, 39.4, 27.2. υ_max_ (ATR/cm^–1^): 2975, 1743, 1614, 1481, 1426, 1207, 1093, 1028. HRMS (ESI^+^) *m*/*z*: calcd for C_14_H_14_O_4_ + H^+^: 247.0962 [M + H]^+^; found 247.0965. Characterization data were consistent with literature values.^60^

#### *S*-Benzylation of Thiourea **8**

Thiourea **8** (1.37 g, 18.0 mmol), 1,3,5-trimethoxybenzene (internal standard for ^1^H NMR spectroscopy; 252 mg, 1.50 mmol) and ethanol (30 mL) were added to a two-necked round bottomed flask (50 mL) equipped with a cross-shaped magnetic stirrer bar. The DS probe was calibrated, and the probe and a digital thermocouple were immersed in the solution through a pierced rubber septum. The solution was stirred (200 rpm) at 60 °C and allowed to thermally equilibrate for 30 min, before a dielectric measurement was made (1–10 GHz). Benzyl chloride **7** (1.73 mL, 15.0 mmol) was then added to initiate the reaction, which was analyzed by DS every min until 5 min, then every 5 min until 120 min. Aliquots of the reaction mixture were removed for off-line analysis by ^1^H NMR spectroscopy at 0.5, 15.5, 30.5, 45.5, 60.5, 90.5, and 120.5 min. Each sample was diluted in DMSO-*d*_6_ prior to analysis by ^1^H NMR spectroscopy. At the end of the reaction, the remaining reaction mixture was allowed to cool to room temperature, then Et_2_O (25 mL) was added. The resulting solid was collected by Büchner filtration, washed with Et_2_O, and dried under a flow of air to give *S*-benzylisothiouronium chloride **9** (1.32 g, 6.50 mmol, 53%) as a colorless solid (m.p. 177–178 °C, lit.^61^ 174 °C). ^1^H NMR (400 MHz, DMSO-*d*_6_): δ 9.28 (br s, 4H), 7.39 (d, *J* = 7.5 Hz, 2H), 7.31 (app. t, *J* = 7.2 Hz, 2H), 7.27–7.23 (m, 1H), 4.49 (s, 2H). ^13^C{^1^H} NMR (100 MHz, DMSO-*d*_6_): δ 168.4, 134.4, 128.1, 127.9, 127.0, 33.2. υ_max_ (ATR/cm^–1^): 3272, 3000, 1643, 1423, 755, 700, 567, 466. HRMS (ESI^+^) *m*/*z*: calcd for C_8_H_11_N_2_S^+^: 167.0637 [M – Cl]^+^; found 167.0642. Characterization data were consistent with literature values.^62^

## Supporting Information Available

Procedures for data analysis, plots of spectroscopic data, summary statistics for PLS models, raw dielectric spectroscopy data, and predicted and measured concentration-time data (PDF)
Supporting data for Figure 4 (XLSX)
Supporting data for Figure 5 (XLSX)
Supporting data for Figure 7 (XLSX)
Supporting data for Figure 8 (XLSX)
