# Supplementary material for: Coaxial Dielectric Spectroscopy as an In-Line Process Analytical Technique for Reaction Monitoring
Source: Org Process Res Dev. 2023 May 30;27(6):1094–103. doi: 10.1021/acs.oprd.3c00081 (PMC10278184; doi:10.1021/acs.oprd.3c00081)

SUPPORTING INFORMATION

Coaxial Dielectric Spectroscopy as an In-Line Process Analytical Technique for Reaction Monitoring

Desiree M. Dalligos,[†‡] Michael J. Pilling,[§*] Georgios Dimitrakis, [†*] and Liam T. Ball[‡*]

[†] Department of Chemical and Environmental Engineering, Coates Building, University of Nottingham, Nottingham NG7 2RD, U.K.

[‡] School of Chemistry, University of Nottingham, Nottingham NG7 2RD, U.K.

[§] Chemical Development, Pharmaceutical Technology & Development, Operations, AstraZeneca, Macclesfield SK10 2NA, U.K.

Corresponding authors:

*Email: michaeljohn.pilling@astrazeneca.com,

*Email: georgios.dimitrakis@nottingham.ac.uk,

*Email: liam.ball@nottingham.ac.uk

Table of Contents

1. **Comparison of DS to Common In-line Analytical Techniques** 3
2. **Calculation of Reaction Profiles from ^{1}H NMR Spectroscopy** 4
3. **Creation of PLS Models** 4
4. **Reaction Monitoring at a Single Frequency (Figure 4B & 4C)** 5
5. **Data From Manuscript Figure 4** 6

Plots of Dielectric Constant (ε′) *vs* Frequency 6

Plots of Dielectric Loss (ε′′) *vs* Frequency 11

Concentration-Time Plots Measured by ^{1}H NMR Spectroscopy and *In Situ* FTIR Spectroscopy 16

6. **Data From Manuscript Figure 5** 19

Plots of Dielectric Constant (ε′) *vs* Frequency 19

Plots of Dielectric Loss (ε′′) *vs* Frequency 25

Concentration-Time Plot Measured by ^{1}H NMR Spectroscopy 31

7. **Data From Manuscript Figure 7** 32

Plots of Dielectric Constant (ε′) *vs* Frequency 32

Plots of Dielectric Loss (ε′′) *vs* Frequency 35

Concentration-Time Plots Measured by ^{1}H NMR Spectroscopy 38

PLS Model and Summary Statistics 41

8. **Data From Manuscript Figure 8** 42

Plots of Dielectric Constant (ε′) *vs* Frequency 42

Plots of Dielectric Loss (ε′′) *vs* Frequency 45

Concentration-Time Plots Measured by ^{1}H NMR Spectroscopy 48

PLS Model and Summary Statistics 51

9. **NMR Spectra of Isolated Compounds** 52

1. Comparison of DS to Common In-line Analytical Techniques

Table S1. Key features and typical requirements / limitations of dielectric spectroscopy and common analytical techniques used for in-line reaction monitoring.

	Mid IR	Raman	UV-Vis	Dielectric Spectroscopy
**Spectral range ***	4000-16000 nm<br>(650-2500 cm^{-1})	2500-10^6 nm<br>(raman shift: 200-3300 cm^{-1})	210-390 nm; 390-870 nm	10^{-6}-10^{12} Hz
**Molecular phenomena observed**	Bond vibrations and rotations	Bond vibrations	Electronic transitions	Induced dipole rotations
**Insight obtained**	Chemical bonding	Chemical bonding	Molecular structure	Molecular structure
**Physical form of sample**	Liquid	Solid or liquid	Liquid	Liquid, gel or malleable† solid
**Signal resolution**	High	High	Low	Very low or non-existent
**Sensitivity**	mM	mM	µM	Sample dependent‡
**Specific sample requirements and limitations**	● Heterogeneous samples cause spectral complexity<br>● Water can give broad absorption bands that mask analyte signals	● Heterogeneous samples give non-representative results<br>● Fluorescent analytes or impurities mask Raman effect<br>● Not applicable to pure metal or alloy samples<br>● Laser irradiation can decompose sensitive samples	● Heterogeneous samples not typically appropriate<br>● Stray light can lead to deviations from the Beer-Lambert law	● Heterogeneous samples only appropriate if domains are small and evenly distributed

* Note: spectral ranges refer to those typical for in-line variants of each technique, which are often limited by hardware requirements (*e.g.*, fibre optics) and which are therefore more restricted than the ranges possible for off-line versions of the same technique. † Sample must be able to contact the probe-end without air gaps. ‡ Sensitivity is very dependent on the properties of the analyte, with measurements at pM concentrations possible for appropriate systems.

2. Calculation of Reaction Profiles from ^{1}H NMR Spectroscopy

Reaction profiles (concentration *vs* time) were calculated from ^{1}H NMR spectroscopy using the relative integrals of the starting material and an internal standard. The resulting profiles were fitted with a second order exponential decay function using the curve fitting tool in MATLAB. The resulting model was used in subsequent univariate and multivariate data analysis.

3. Creation of PLS Models

Partial Least Squares (PLS) analysis was performed using EigenVector's PLS_toolbox plug-in for MATLAB. Dielectric spectroscopic data (signal intensity *vs* frequency) were used as the **X** matrix, and reagent concentration was used as the **Y** vector. The number of components was determined by cross-validation where the variance captured is >95% in both **X** and **Y**. A PLS model was generated and then the model was validated with new, independent data sets.

4. Reaction Monitoring at a Single Frequency (Figure 4B & 4C)

(1) Extract the DS signal intensity at a single frequency across all time-points;

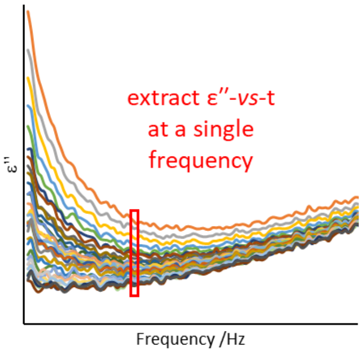

(2) Plot DS signal intensity at that frequency as a function of time;

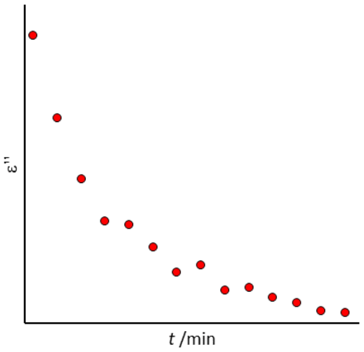

(3) Normalise the resulting DS-time curve such that the initial and final points correspond to the reagent concentrations determined by ^{1}H NMR spectroscopic analysis, giving a concentration-time curve;

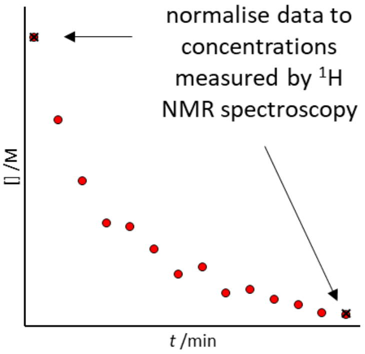

(4) Calculate the residual sum of squares between the concentration-time curve measured with DS, and the concentration-time curve measured using ^{1}H NMR spectroscopy.

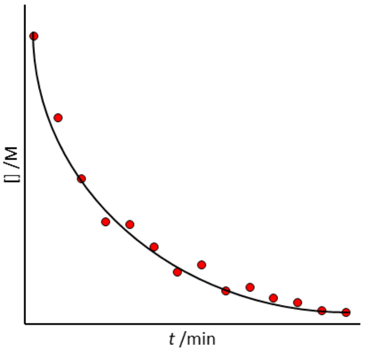

This process was repeated at each frequency of dielectric constant and dielectric loss. In each case, the frequency giving the lowest residual sum of squares was identified as most appropriate for univariate (single frequency) data analysis.

5. Data From Manuscript Figure 4

Plots of Dielectric Constant (ε′) *vs* Frequency

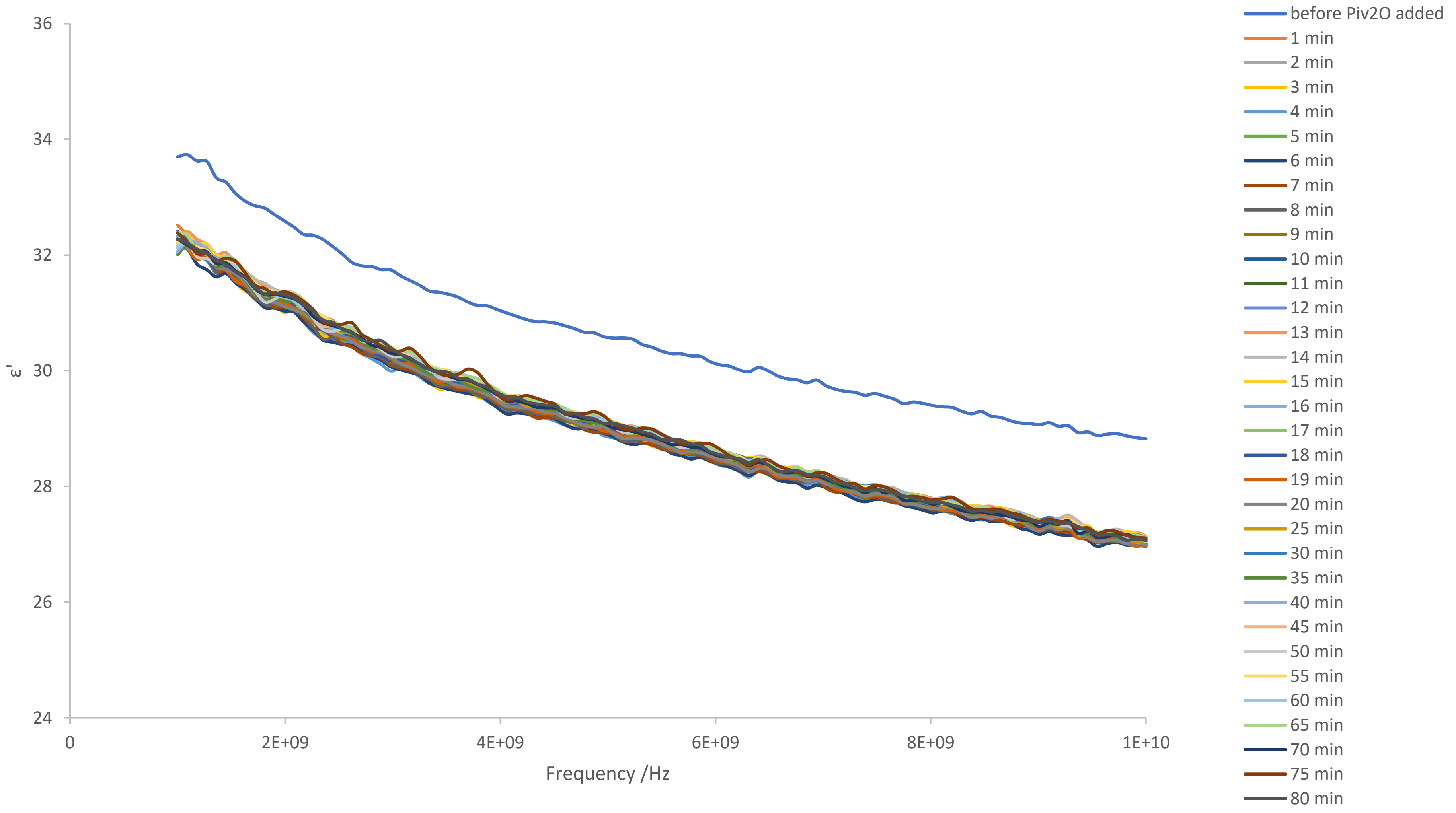

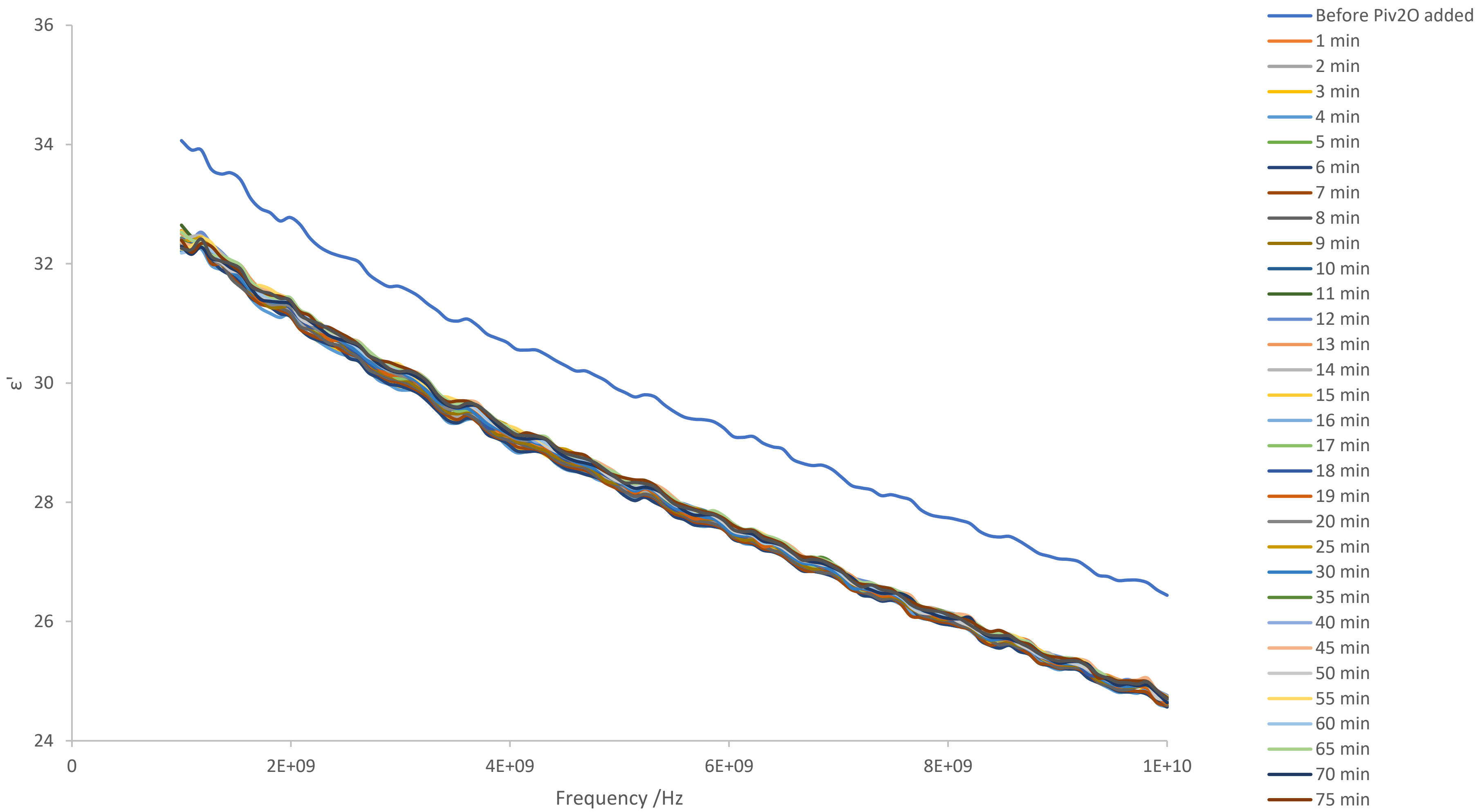

36
34
32
30
28
26
24
ε'
0
2E+09
4E+09
6E+09
8E+09
1E+10
Frequency /Hz
Before Piv2O added
1 min
2 min
3 min
4 min
5 min
6 min
7 min
8 min
9 min
10 min
11 min
12 min
13 min
14 min
15 min
16 min
17 min
18 min
19 min
20 min
25 min
30 min
35 min
40 min
45 min
50 min
55 min
60 min
65 min
70 min
75 min
80 min

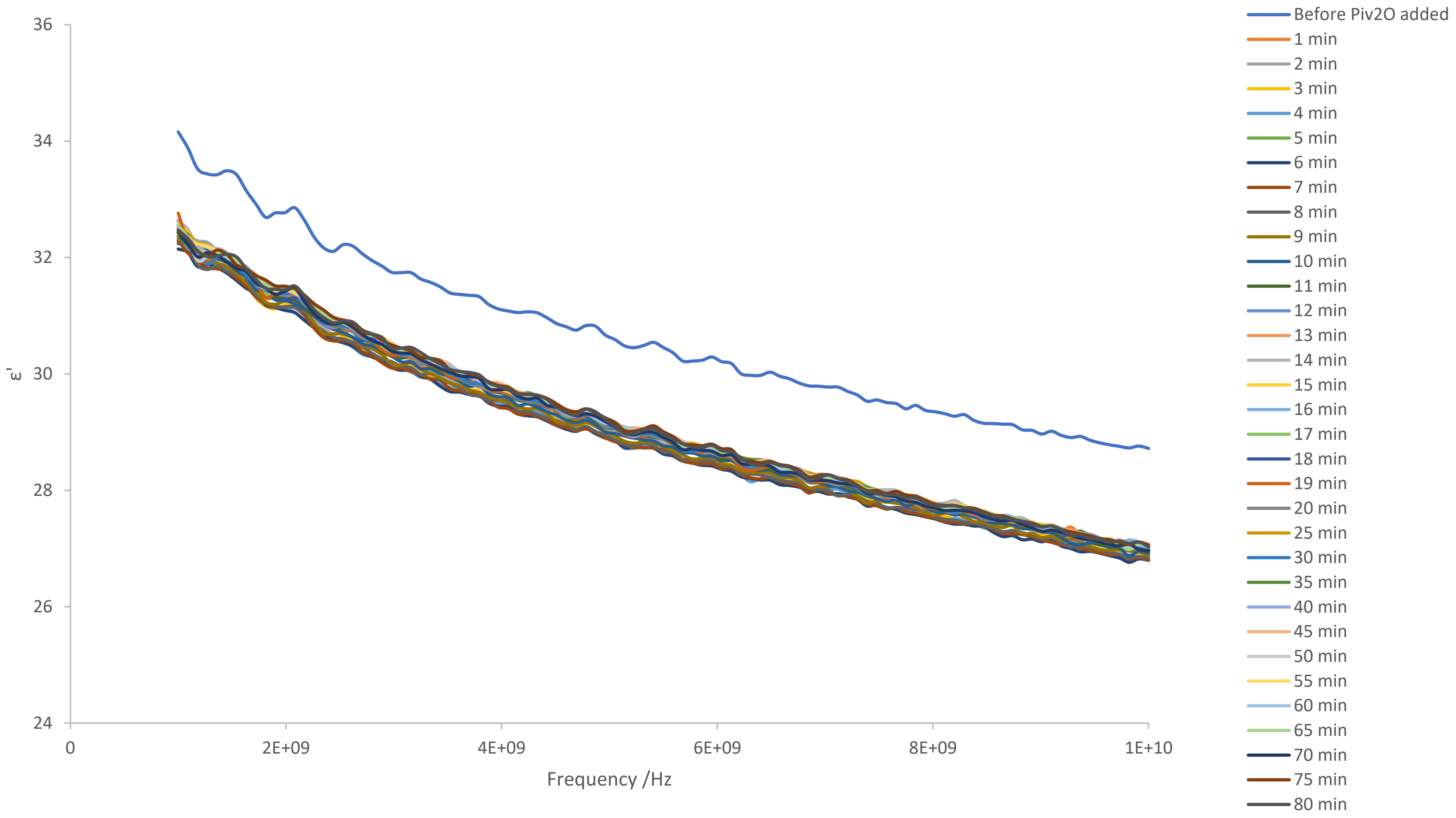

36
34
32
30
28
26
24
ε'
0
2E+09
4E+09
6E+09
8E+09
1E+10
Frequency /Hz
Before Piv2O added
1 min
2 min
3 min
4 min
5 min
6 min
7 min
8 min
9 min
10 min
11 min
12 min
13 min
14 min
15 min
16 min
17 min
18 min
19 min
20 min
25 min
30 min
35 min
40 min
45 min
50 min
55 min
60 min
65 min
70 min
75 min
80 min

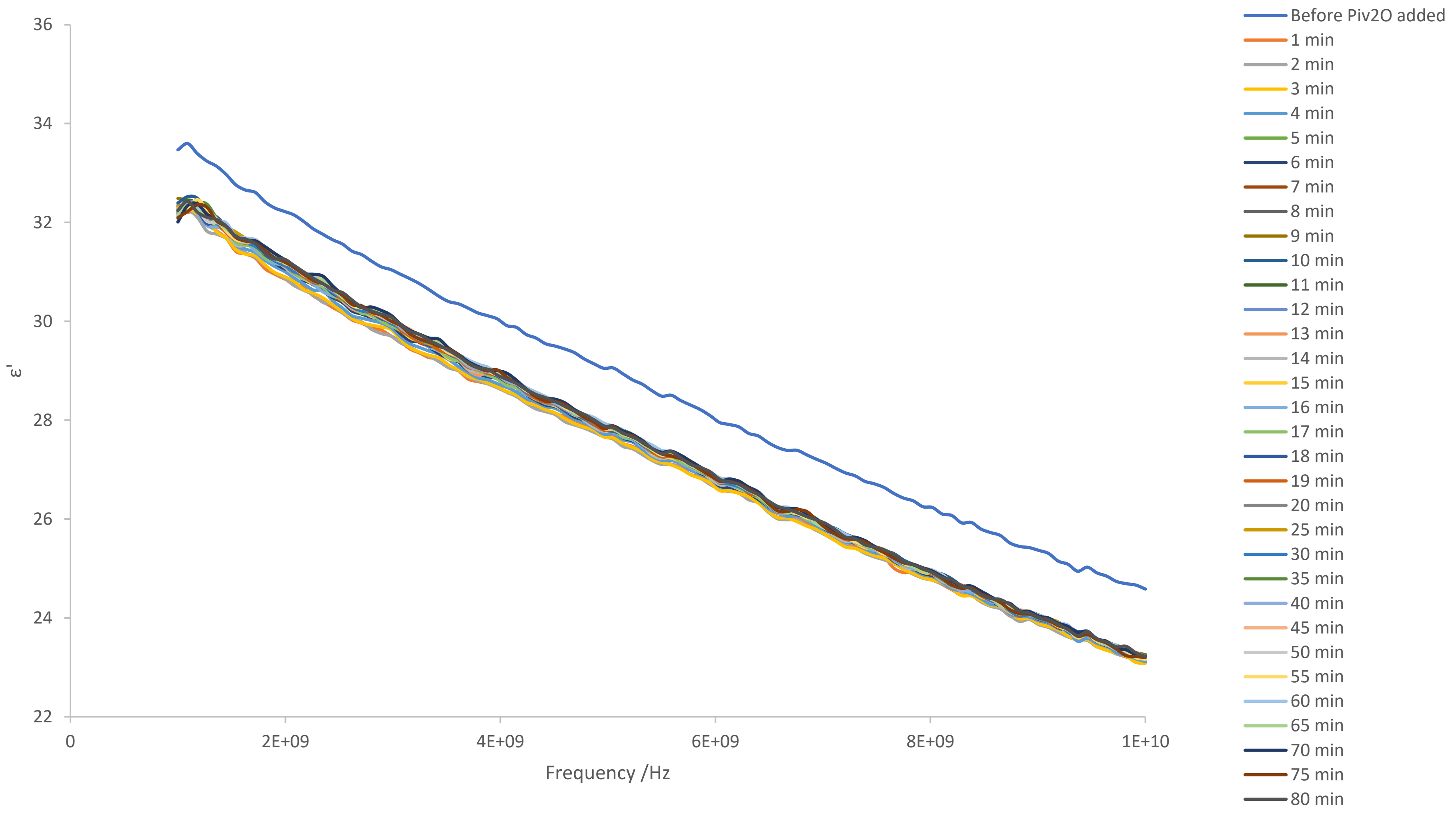
36
34
32
30
28
26
24
22
ε'
0
2E+09
4E+09
6E+09
8E+09
1E+10
Frequency /Hz
Before Piv2O added
1 min
2 min
3 min
4 min
5 min
6 min
7 min
8 min
9 min
10 min
11 min
12 min
13 min
14 min
15 min
16 min
17 min
18 min
19 min
20 min
25 min
30 min
35 min
40 min
45 min
50 min
55 min
60 min
65 min
70 min
75 min
80 min

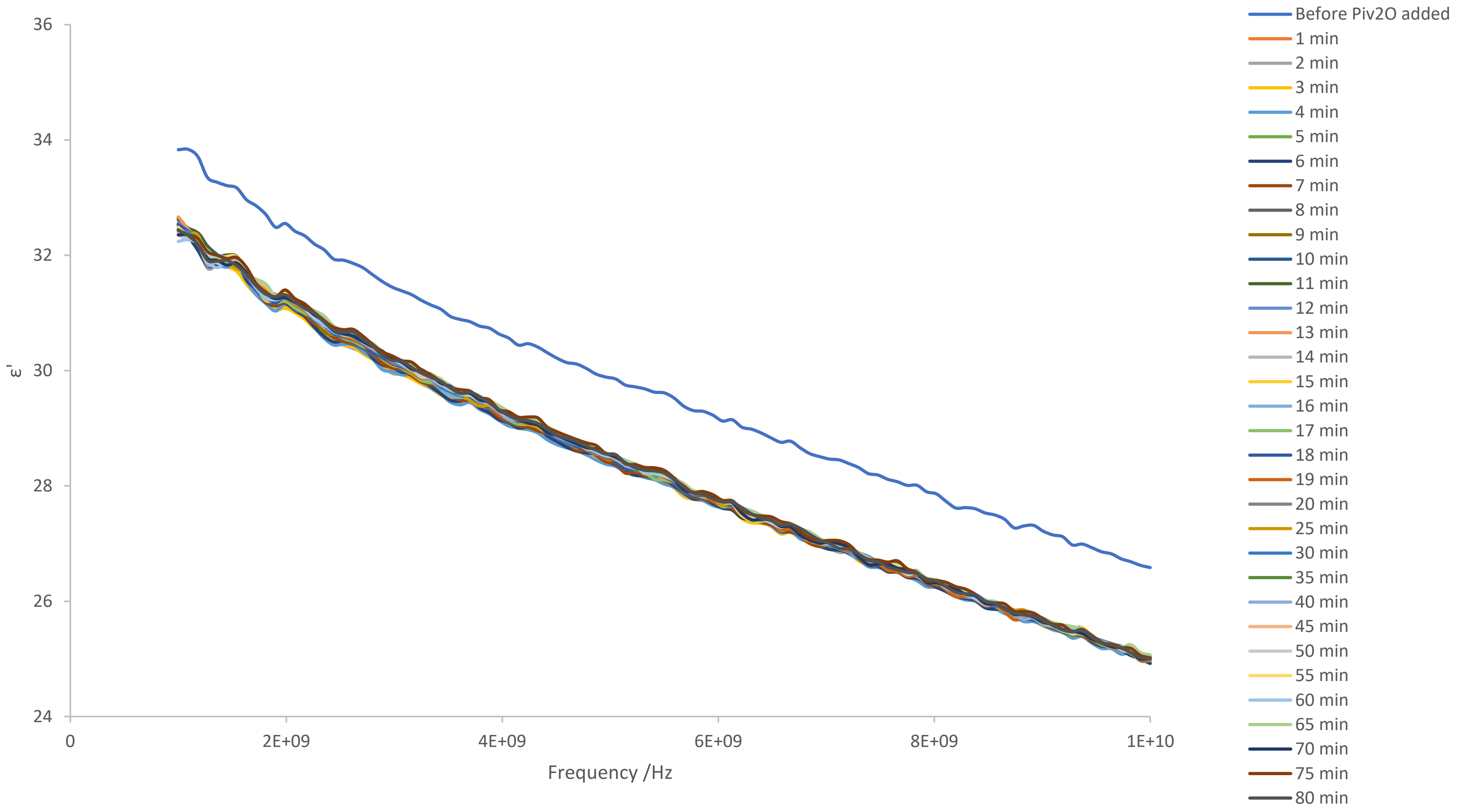

36
34
32
30
28
26
24
ε'
0
2E+09
4E+09
6E+09
8E+09
1E+10
Frequency /Hz
Before Piv2O added
1 min
2 min
3 min
4 min
5 min
6 min
7 min
8 min
9 min
10 min
11 min
12 min
13 min
14 min
15 min
16 min
17 min
18 min
19 min
20 min
25 min
30 min
35 min
40 min
45 min
50 min
55 min
60 min
65 min
70 min
75 min
80 min

Plots of Dielectric Loss (ε'') *vs* Frequency

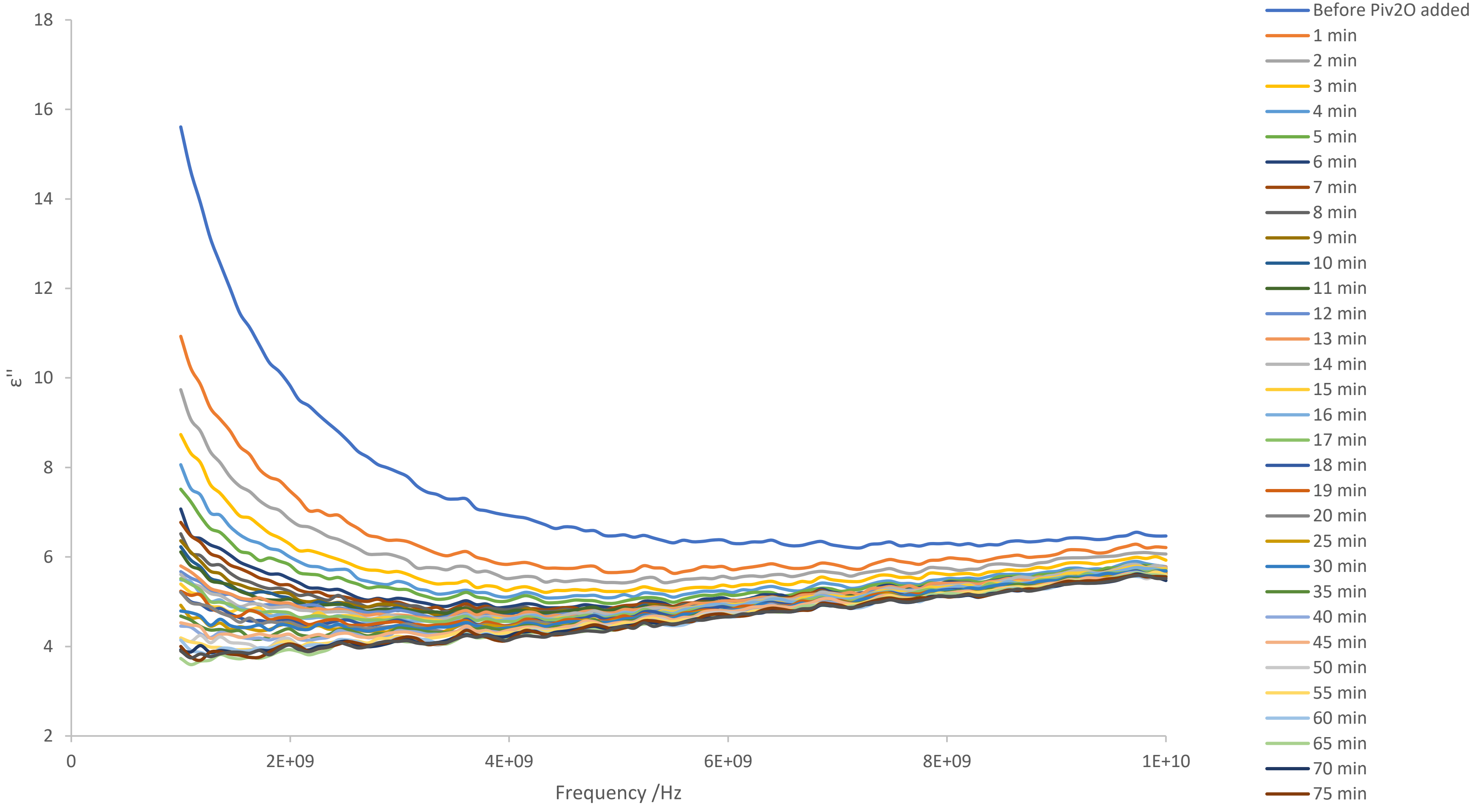

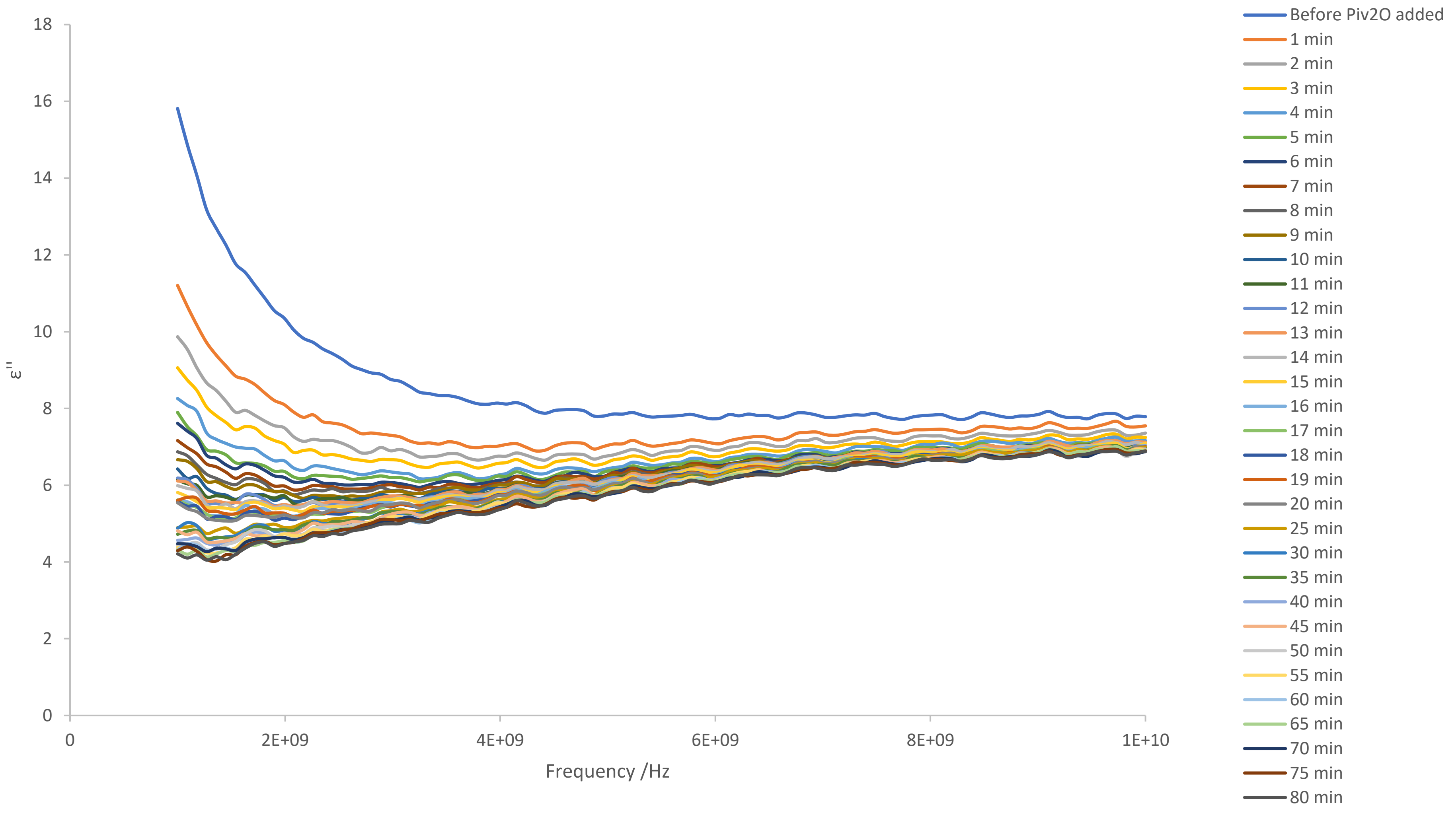
18
16
14
12
10
8
6
4
2
0
ε''
0
2E+09
4E+09
6E+09
8E+09
1E+10
Frequency /Hz
Before Piv2O added
1 min
2 min
3 min
4 min
5 min
6 min
7 min
8 min
9 min
10 min
11 min
12 min
13 min
14 min
15 min
16 min
17 min
18 min
19 min
20 min
25 min
30 min
35 min
40 min
45 min
50 min
55 min
60 min
65 min
70 min
75 min
80 min

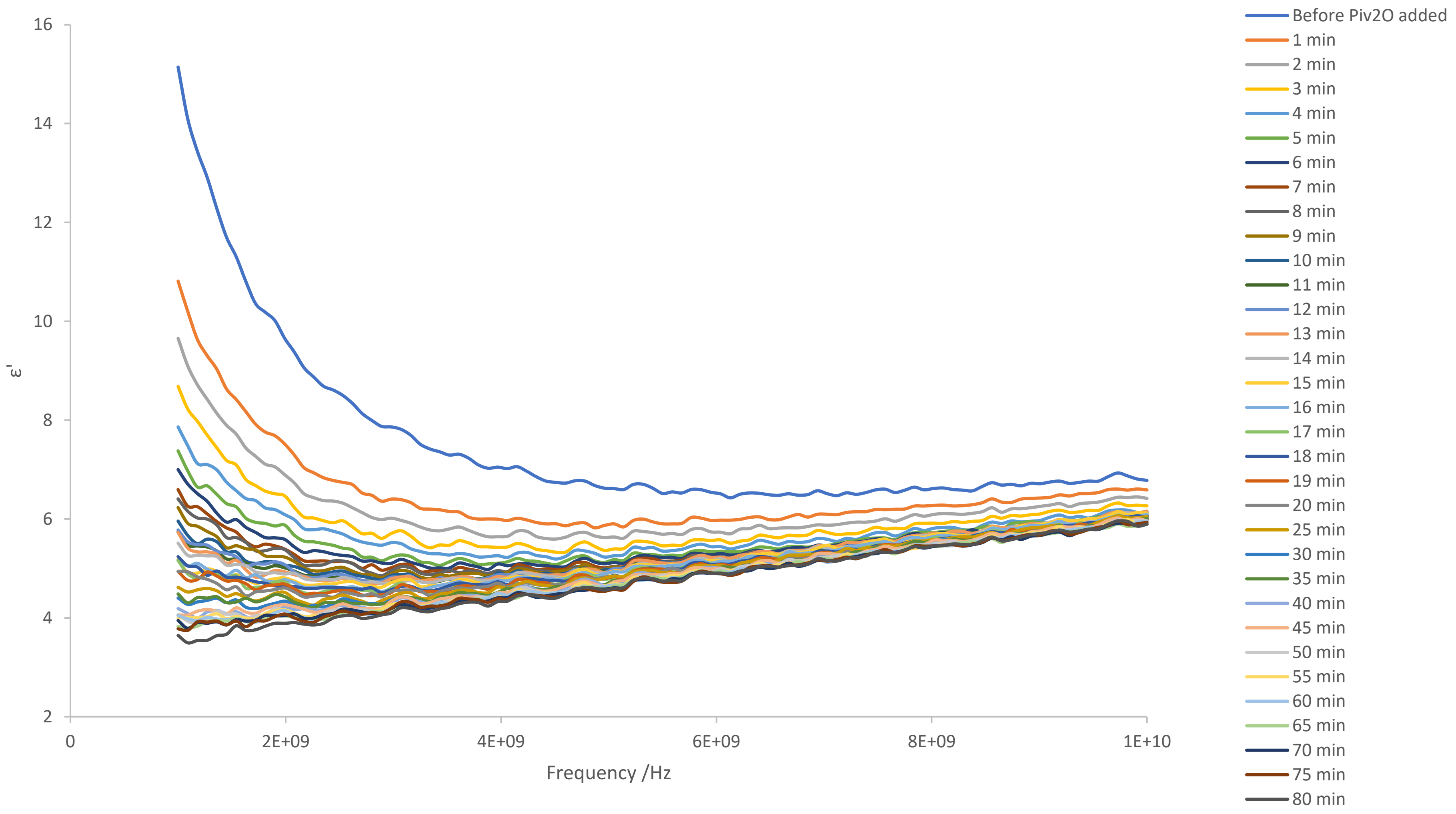
16
14
12
10
8
6
4
2
ε'
0
2E+09
4E+09
6E+09
8E+09
1E+10
Frequency /Hz
Before Piv2O added
1 min
2 min
3 min
4 min
5 min
6 min
7 min
8 min
9 min
10 min
11 min
12 min
13 min
14 min
15 min
16 min
17 min
18 min
19 min
20 min
25 min
30 min
35 min
40 min
45 min
50 min
55 min
60 min
65 min
70 min
75 min
80 min

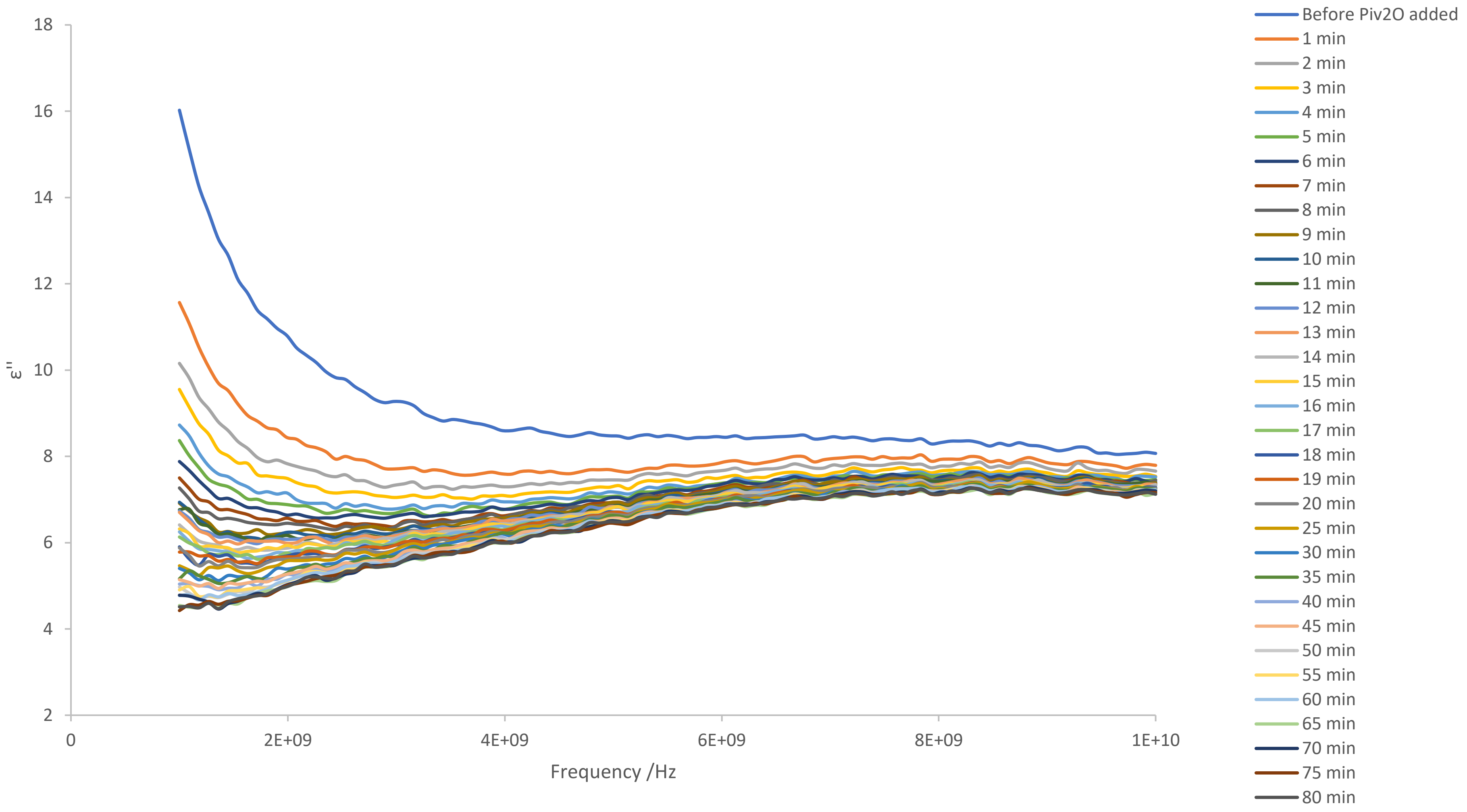
18
16
14
12
10
8
6
4
2
ε''
0
2E+09
4E+09
6E+09
8E+09
1E+10
Frequency /Hz
Before Piv2O added
1 min
2 min
3 min
4 min
5 min
6 min
7 min
8 min
9 min
10 min
11 min
12 min
13 min
14 min
15 min
16 min
17 min
18 min
19 min
20 min
25 min
30 min
35 min
40 min
45 min
50 min
55 min
60 min
65 min
70 min
75 min
80 min

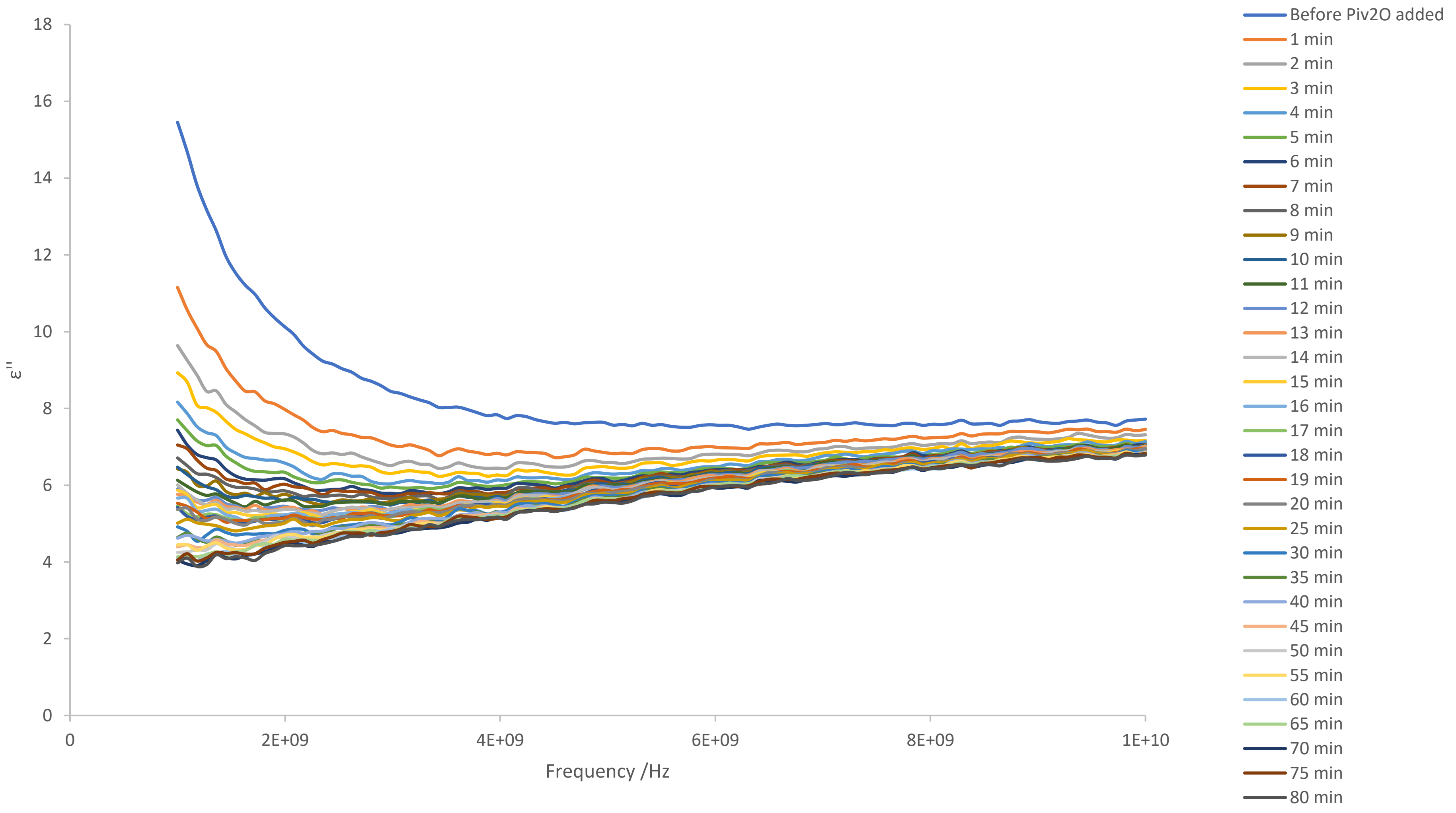
18
16
14
12
10
8
6
4
2
0
ε''
0
2E+09
4E+09
6E+09
8E+09
1E+10
Frequency /Hz
Before Piv2O added
1 min
2 min
3 min
4 min
5 min
6 min
7 min
8 min
9 min
10 min
11 min
12 min
13 min
14 min
15 min
16 min
17 min
18 min
19 min
20 min
25 min
30 min
35 min
40 min
45 min
50 min
55 min
60 min
65 min
70 min
75 min
80 min

Concentration-Time Plots Measured by ^{1}H NMR Spectroscopy and *In Situ* FTIR Spectroscopy

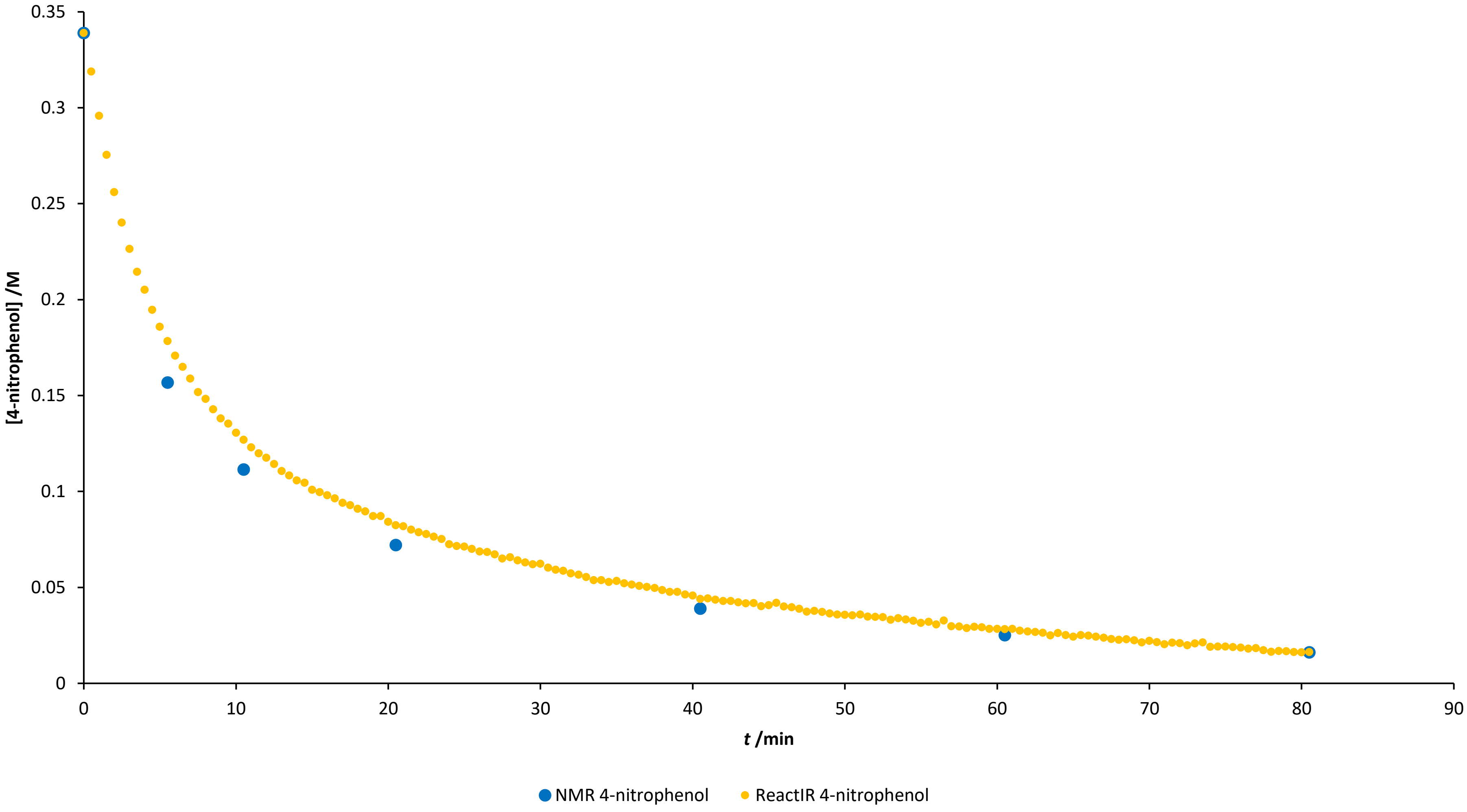

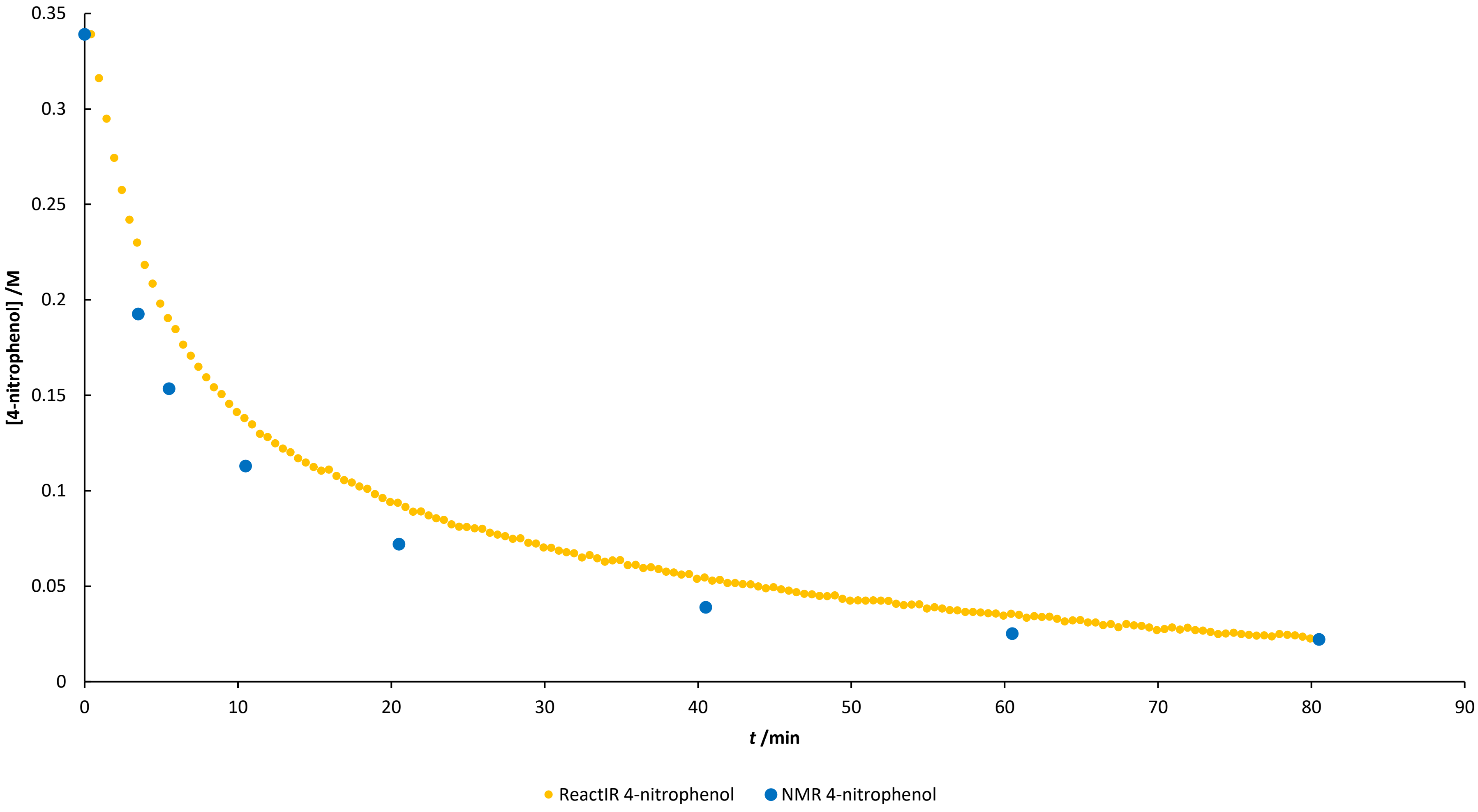

0.35
0.3
0.25
0.2
0.15
0.1
0.05
0
[4-nitrophenol] /M
0
10
20
30
40
50
60
70
80
90
t /min
ReactIR 4-nitrophenol
NMR 4-nitrophenol

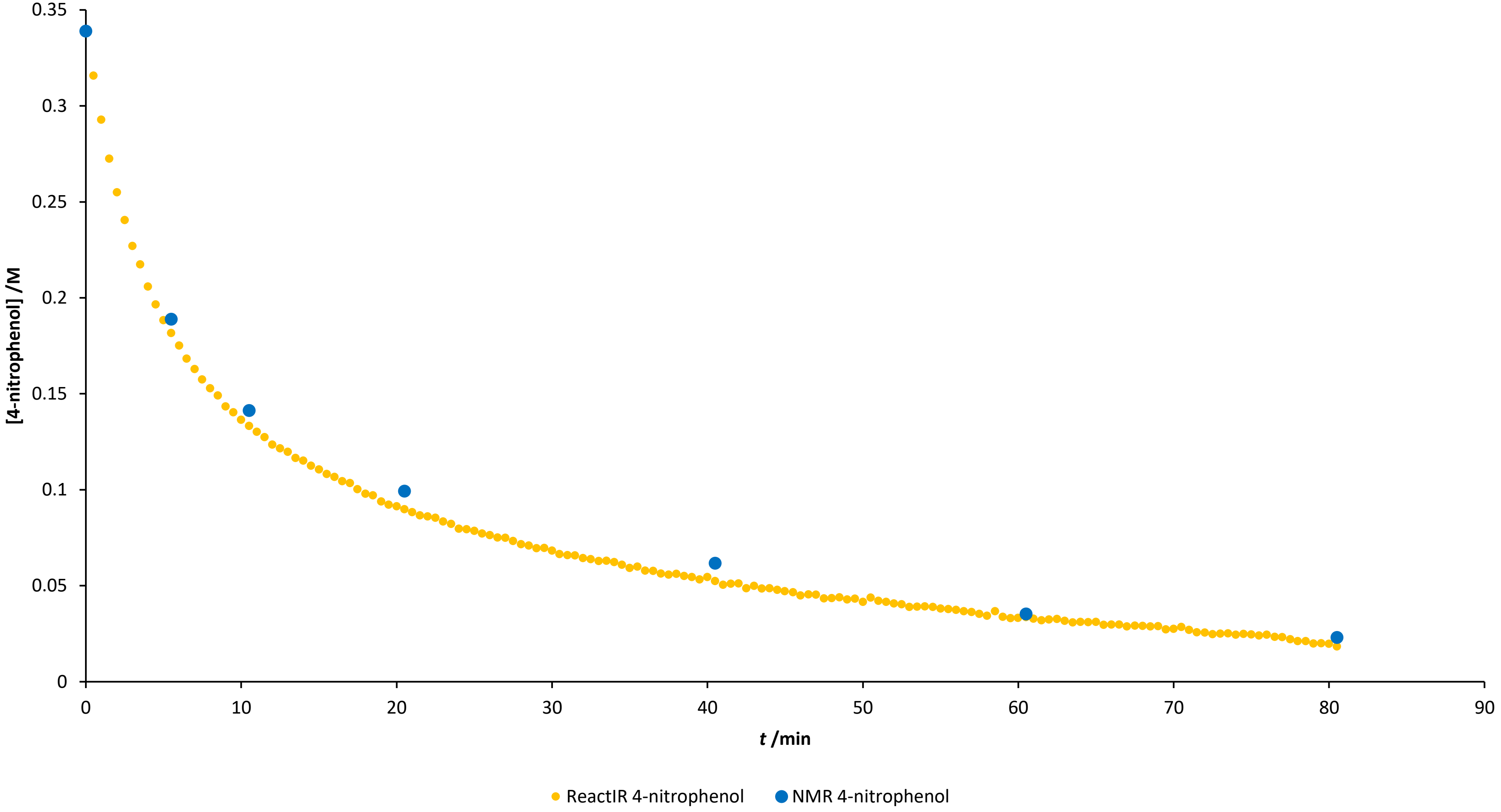
0.35
0.3
0.25
0.2
0.15
0.1
0.05
0
[4-nitrophenol] /M
0
10
20
30
40
50
60
70
80
90
t /min
ReactIR 4-nitrophenol
NMR 4-nitrophenol

6. Data From Manuscript Figure 5

Plots of Dielectric Constant (ε′) *vs* Frequency

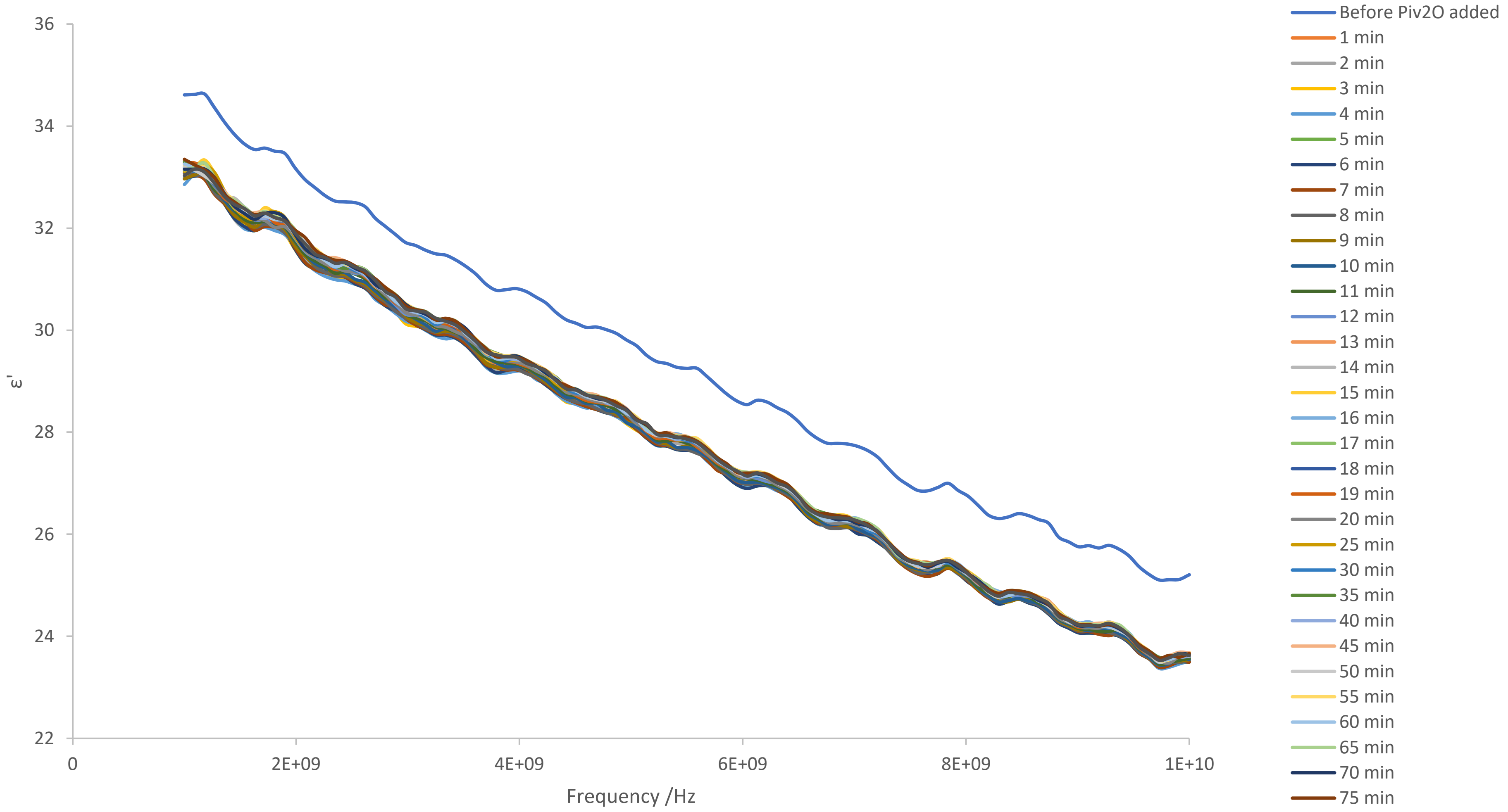

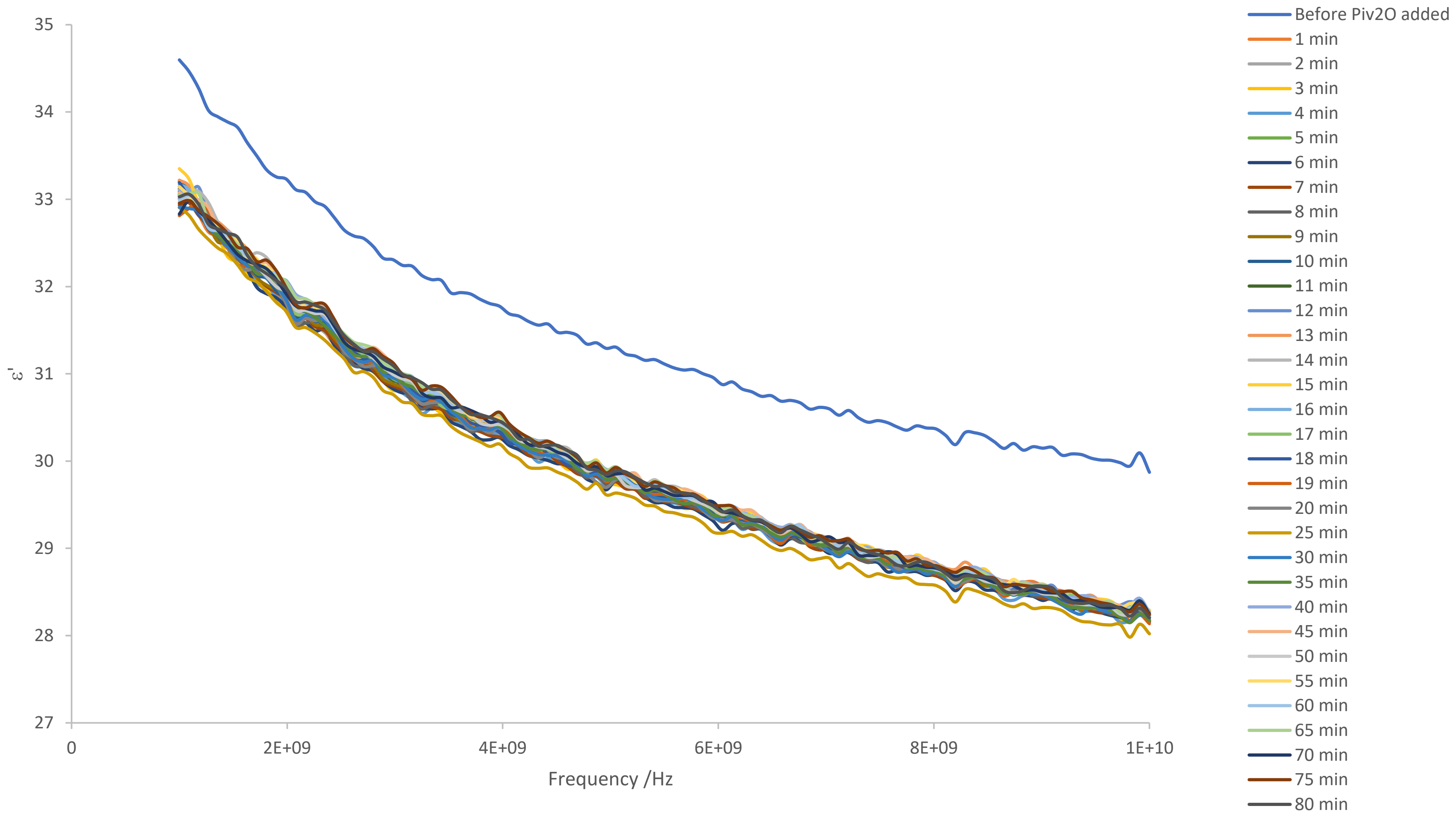

35
34
33
32
31
30
29
28
27
ε'
0
2E+09
4E+09
6E+09
8E+09
1E+10
Frequency /Hz
Before Piv2O added
1 min
2 min
3 min
4 min
5 min
6 min
7 min
8 min
9 min
10 min
11 min
12 min
13 min
14 min
15 min
16 min
17 min
18 min
19 min
20 min
25 min
30 min
35 min
40 min
45 min
50 min
55 min
60 min
65 min
70 min
75 min
80 min

S20

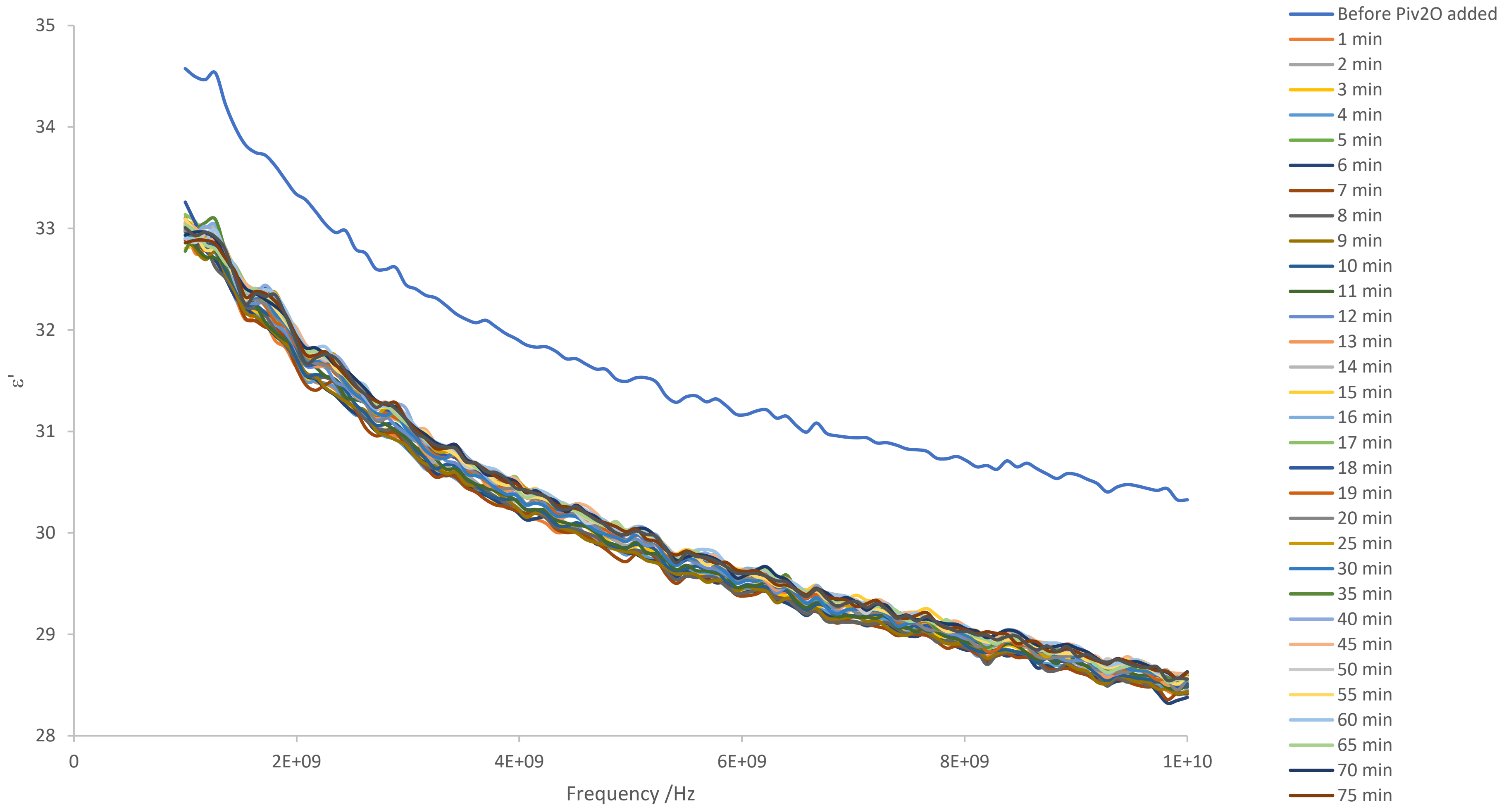
35
34
33
32
31
30
29
28
ε'
0
2E+09
4E+09
6E+09
8E+09
1E+10
Frequency /Hz
Before Piv2O added
1 min
2 min
3 min
4 min
5 min
6 min
7 min
8 min
9 min
10 min
11 min
12 min
13 min
14 min
15 min
16 min
17 min
18 min
19 min
20 min
25 min
30 min
35 min
40 min
45 min
50 min
55 min
60 min
65 min
70 min
75 min
80 min

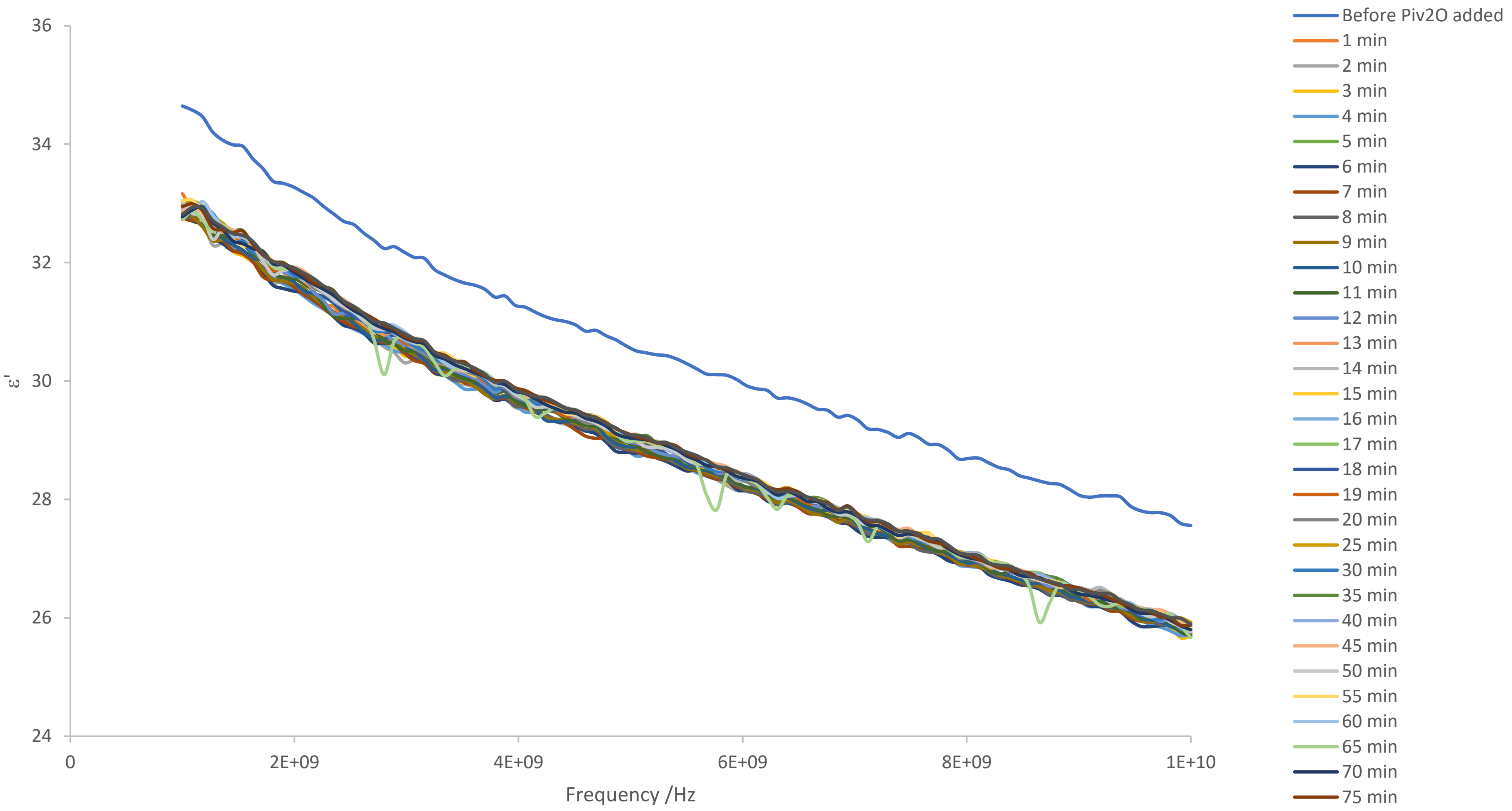

36
34
32
30
28
26
24
ε'
0
2E+09
4E+09
6E+09
8E+09
1E+10
Frequency /Hz
Before Piv2O added
1 min
2 min
3 min
4 min
5 min
6 min
7 min
8 min
9 min
10 min
11 min
12 min
13 min
14 min
15 min
16 min
17 min
18 min
19 min
20 min
25 min
30 min
35 min
40 min
45 min
50 min
55 min
60 min
65 min
70 min
75 min
80 min

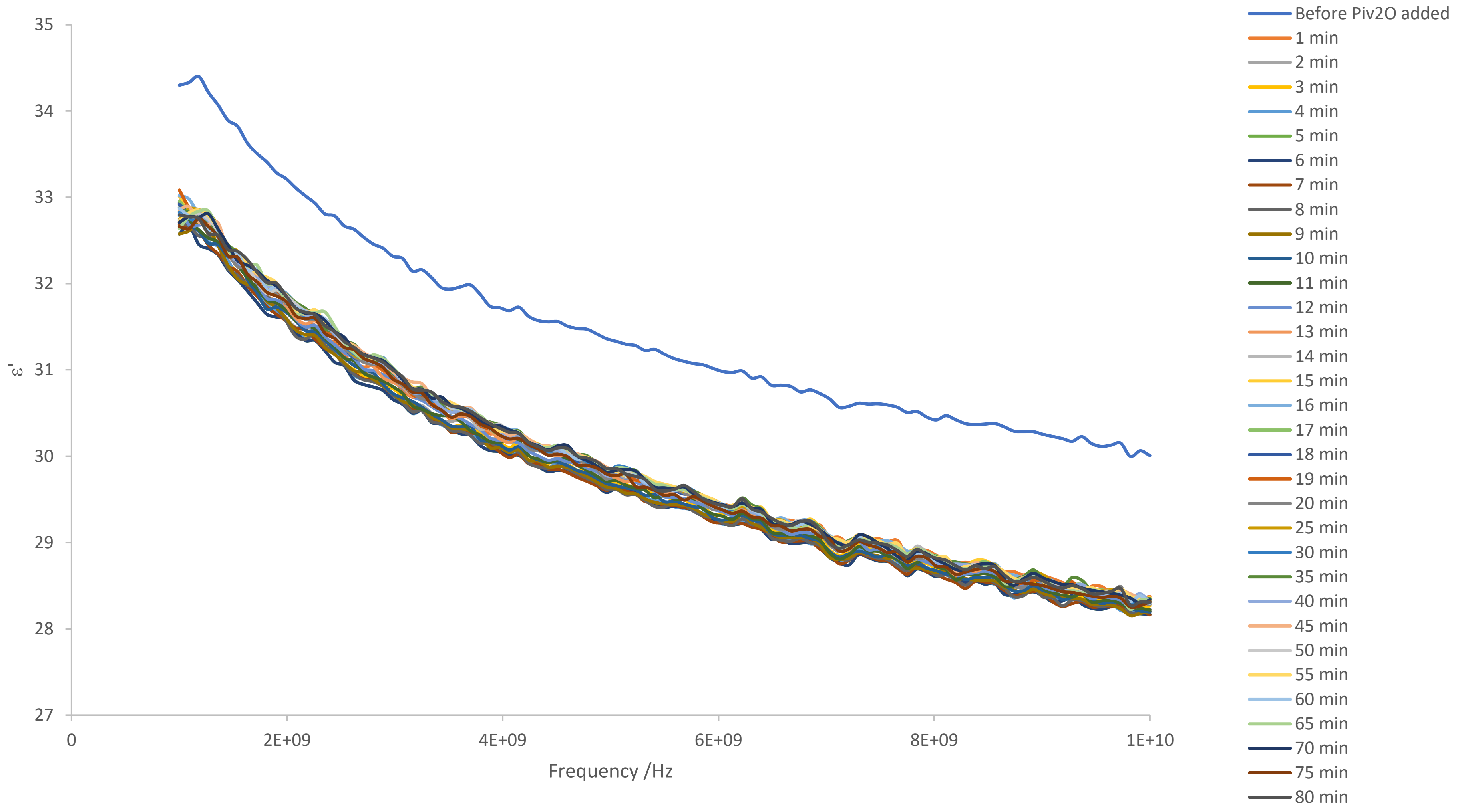

35
34
33
32
31
30
29
28
27
ε'
0
2E+09
4E+09
6E+09
8E+09
1E+10
Frequency /Hz
Before Piv2O added
1 min
2 min
3 min
4 min
5 min
6 min
7 min
8 min
9 min
10 min
11 min
12 min
13 min
14 min
15 min
16 min
17 min
18 min
19 min
20 min
25 min
30 min
35 min
40 min
45 min
50 min
55 min
60 min
65 min
70 min
75 min
80 min

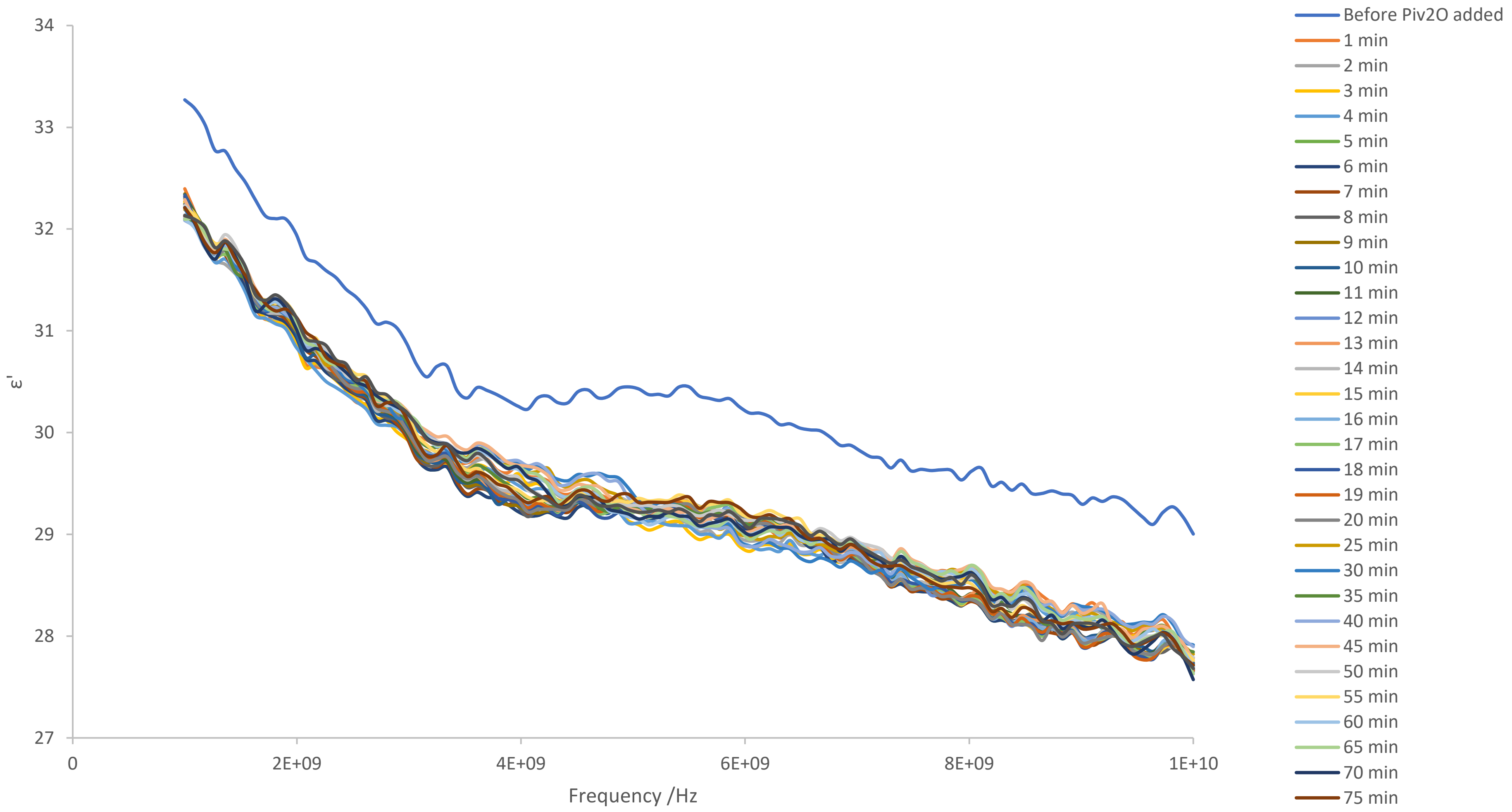

34
33
32
31
30
29
28
27
ε'
0
2E+09
4E+09
6E+09
8E+09
1E+10
Frequency /Hz
Before Piv2O added
1 min
2 min
3 min
4 min
5 min
6 min
7 min
8 min
9 min
10 min
11 min
12 min
13 min
14 min
15 min
16 min
17 min
18 min
19 min
20 min
25 min
30 min
35 min
40 min
45 min
50 min
55 min
60 min
65 min
70 min
75 min
80 min

Plots of Dielectric Loss (ε'') *vs* Frequency

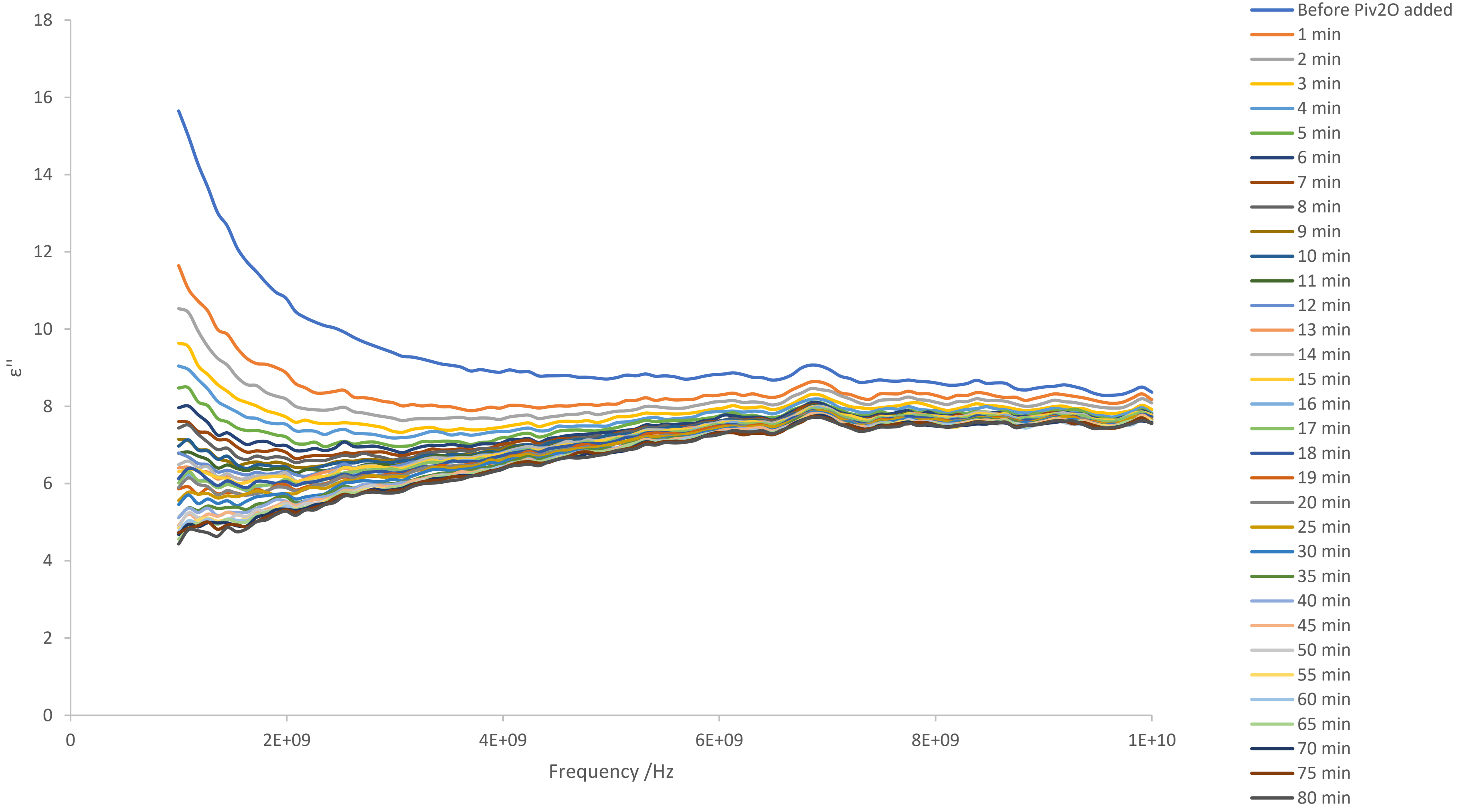

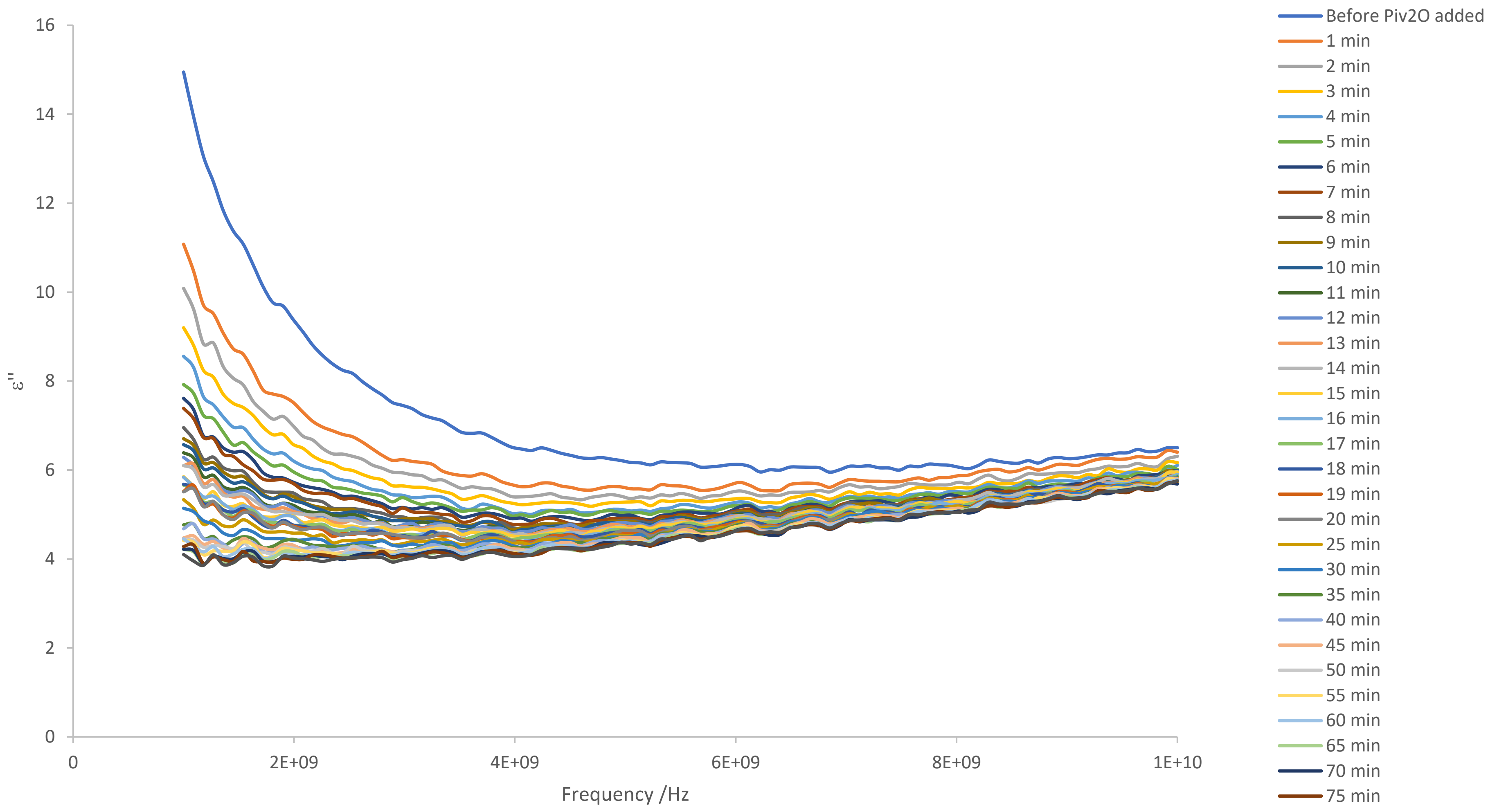
16
14
12
10
8
6
4
2
0
ε''
0
2E+09
4E+09
6E+09
8E+09
1E+10
Frequency /Hz
Before Piv2O added
1 min
2 min
3 min
4 min
5 min
6 min
7 min
8 min
9 min
10 min
11 min
12 min
13 min
14 min
15 min
16 min
17 min
18 min
19 min
20 min
25 min
30 min
35 min
40 min
45 min
50 min
55 min
60 min
65 min
70 min
75 min
80 min

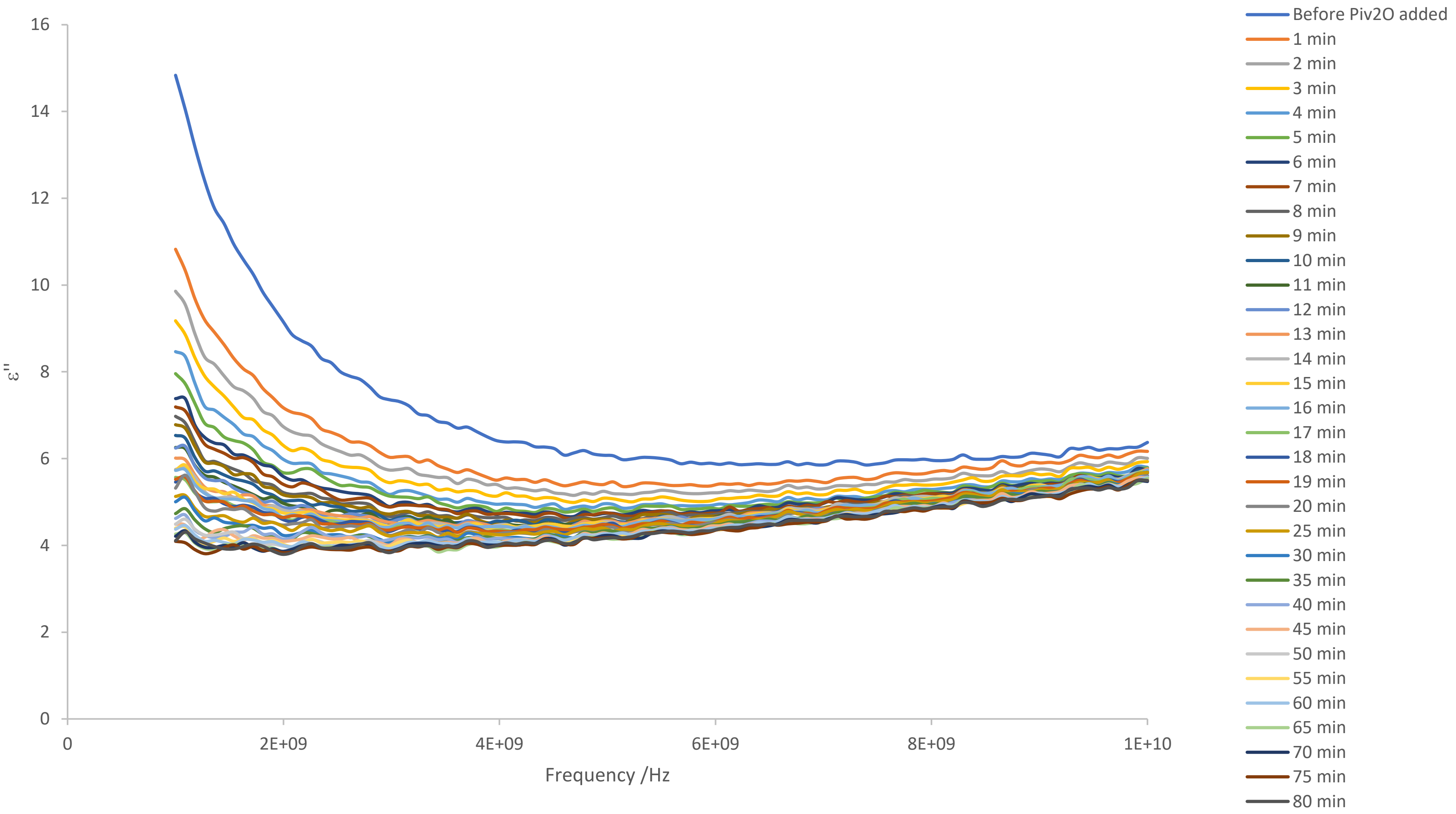

16
14
12
10
8
6
4
2
0
ε''
0
2E+09
4E+09
6E+09
8E+09
1E+10
Frequency /Hz
Before Piv2O added
1 min
2 min
3 min
4 min
5 min
6 min
7 min
8 min
9 min
10 min
11 min
12 min
13 min
14 min
15 min
16 min
17 min
18 min
19 min
20 min
25 min
30 min
35 min
40 min
45 min
50 min
55 min
60 min
65 min
70 min
75 min
80 min

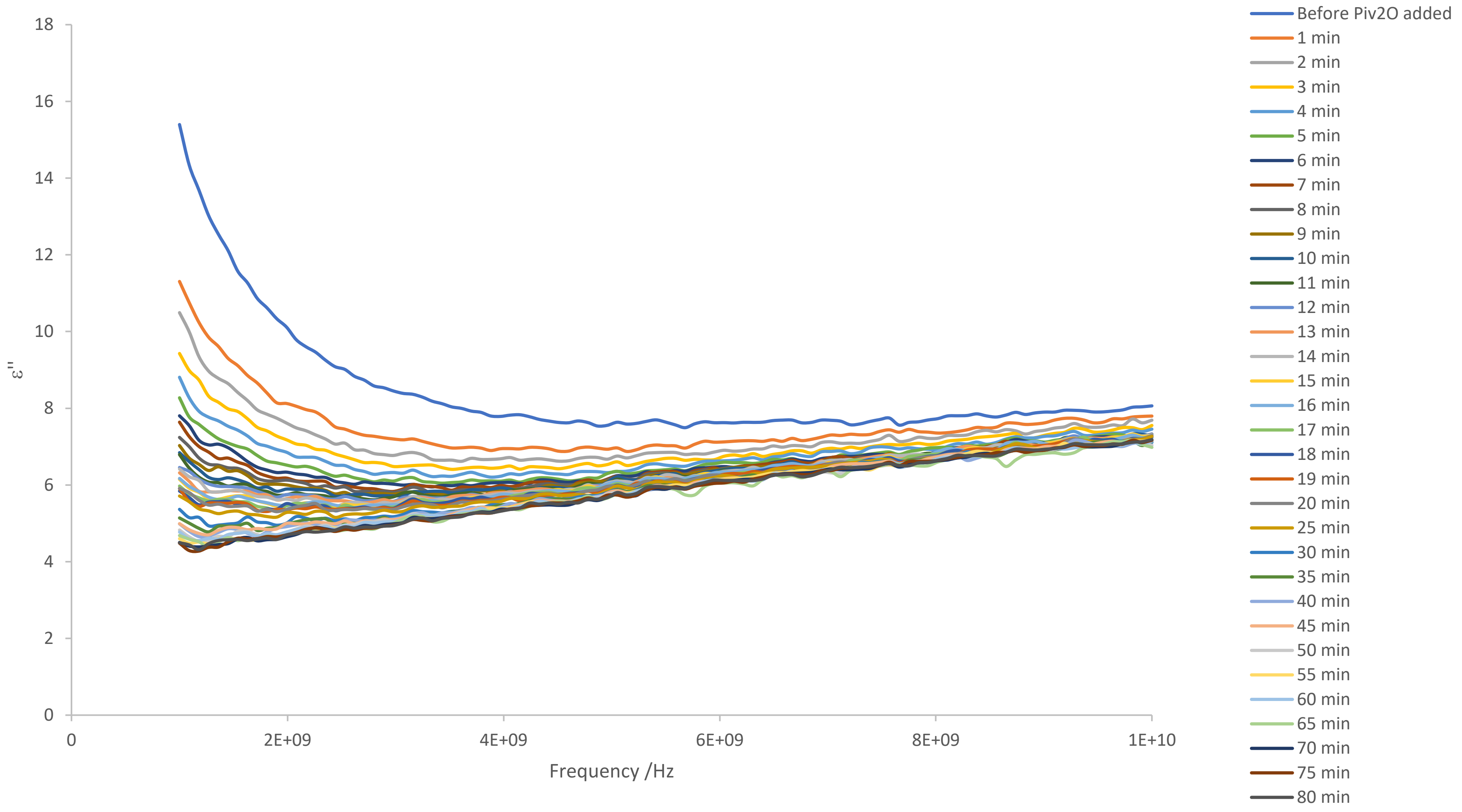

18
16
14
12
10
8
6
4
2
0
ε''
0
2E+09
4E+09
6E+09
8E+09
1E+10
Frequency /Hz
Before Piv2O added
1 min
2 min
3 min
4 min
5 min
6 min
7 min
8 min
9 min
10 min
11 min
12 min
13 min
14 min
15 min
16 min
17 min
18 min
19 min
20 min
25 min
30 min
35 min
40 min
45 min
50 min
55 min
60 min
65 min
70 min
75 min
80 min

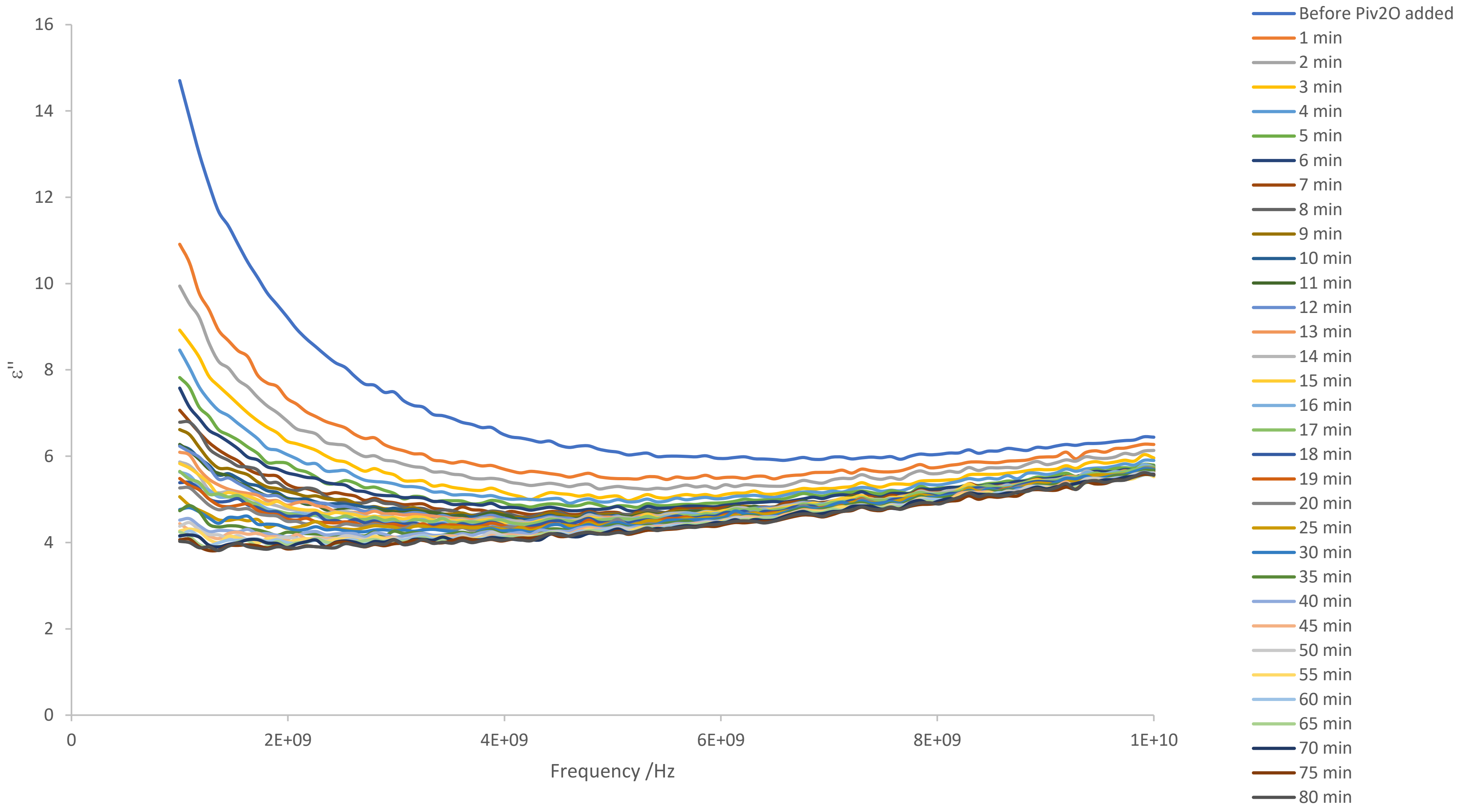

16
14
12
10
8
6
4
2
0
ε''
0
2E+09
4E+09
6E+09
8E+09
1E+10
Frequency /Hz
Before Piv2O added
1 min
2 min
3 min
4 min
5 min
6 min
7 min
8 min
9 min
10 min
11 min
12 min
13 min
14 min
15 min
16 min
17 min
18 min
19 min
20 min
25 min
30 min
35 min
40 min
45 min
50 min
55 min
60 min
65 min
70 min
75 min
80 min

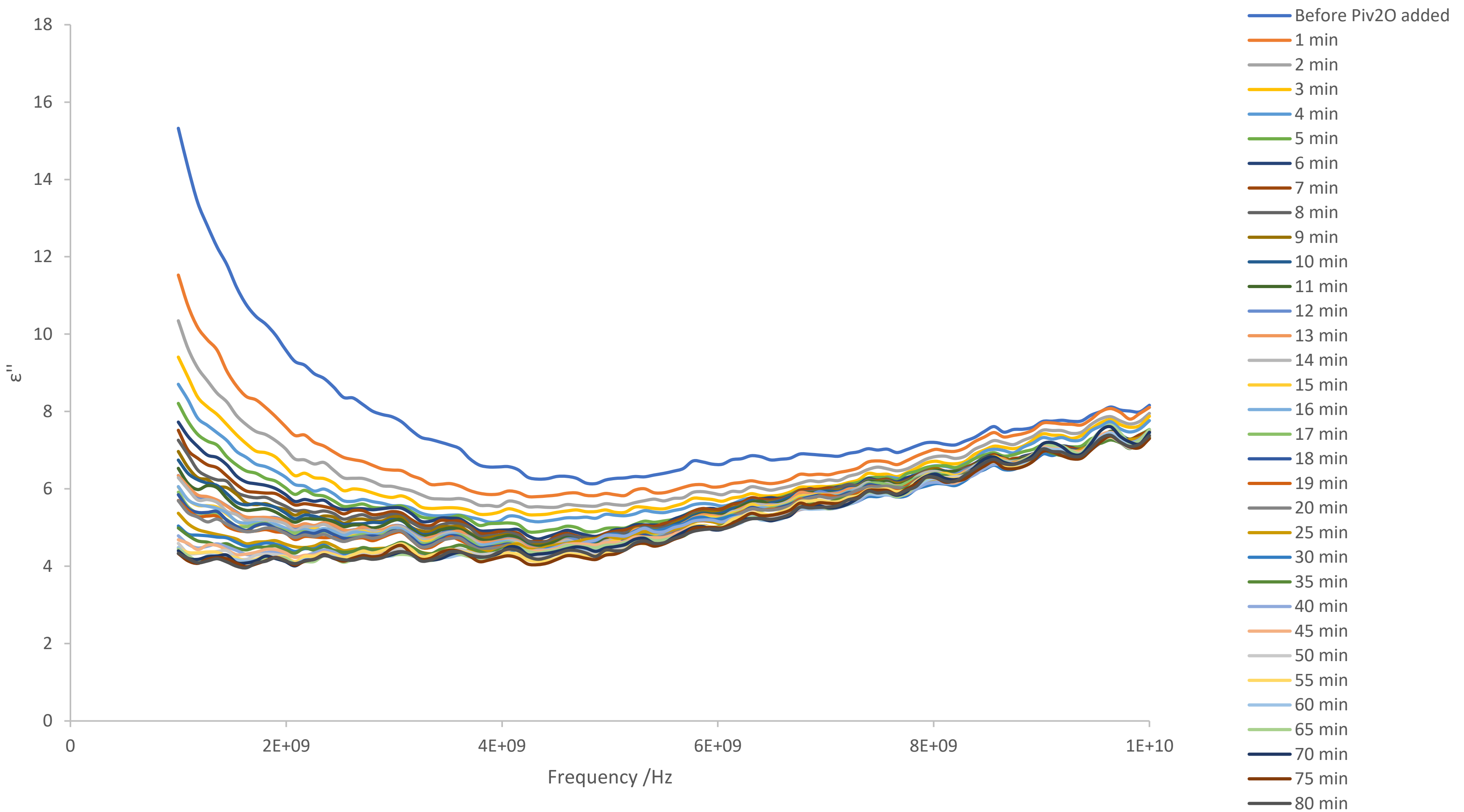

ε''
Frequency /Hz
0
2
4
6
8
10
12
14
16
18
0
2E+09
4E+09
6E+09
8E+09
1E+10
Before Piv2O added
1 min
2 min
3 min
4 min
5 min
6 min
7 min
8 min
9 min
10 min
11 min
12 min
13 min
14 min
15 min
16 min
17 min
18 min
19 min
20 min
25 min
30 min
35 min
40 min
45 min
50 min
55 min
60 min
65 min
70 min
75 min
80 min

S30

Concentration-Time Plot Measured by ^{1}H NMR Spectroscopy

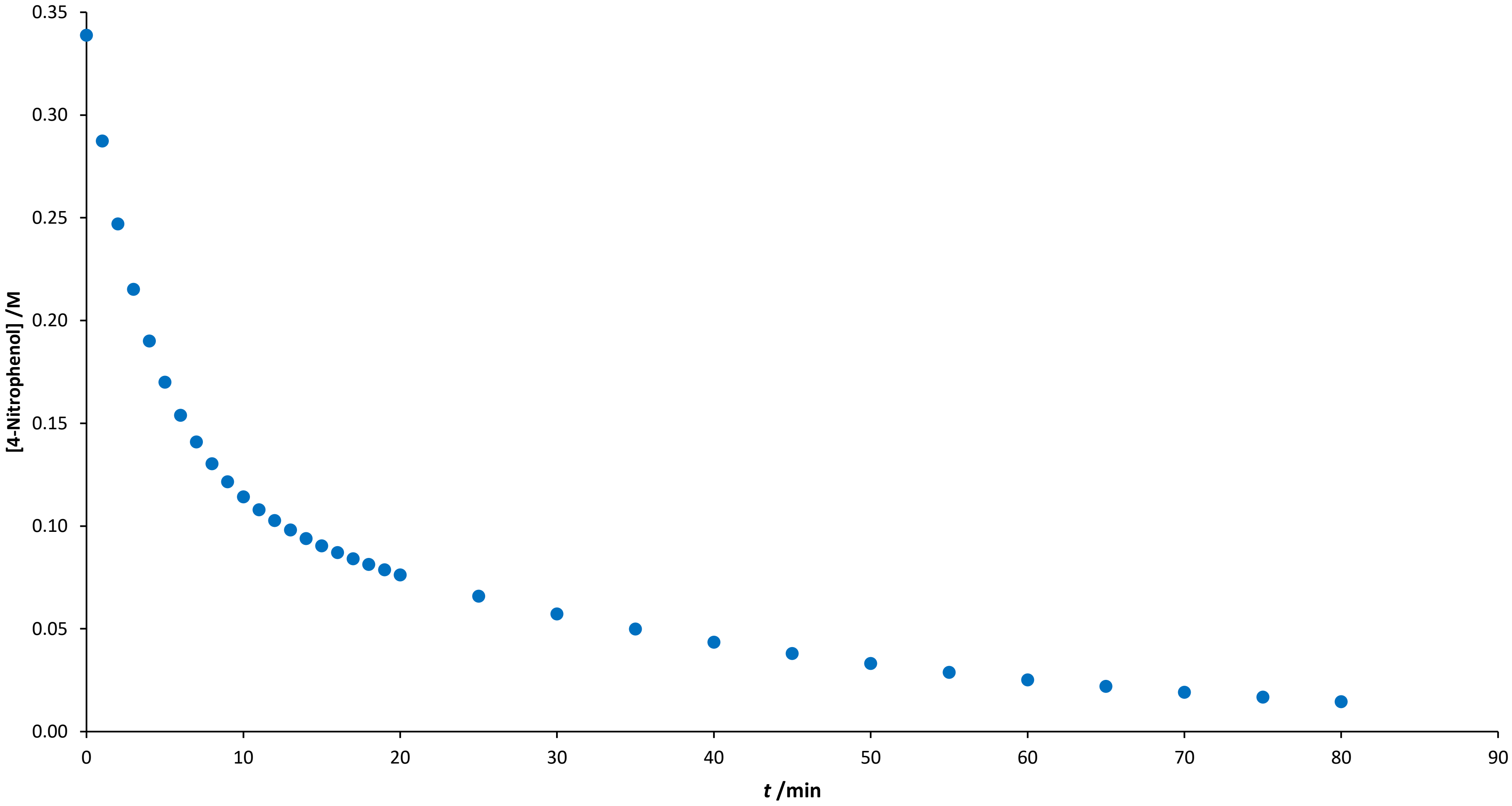

7. Data From Manuscript Figure 7

Plots of Dielectric Constant (ε’) *vs* Frequency

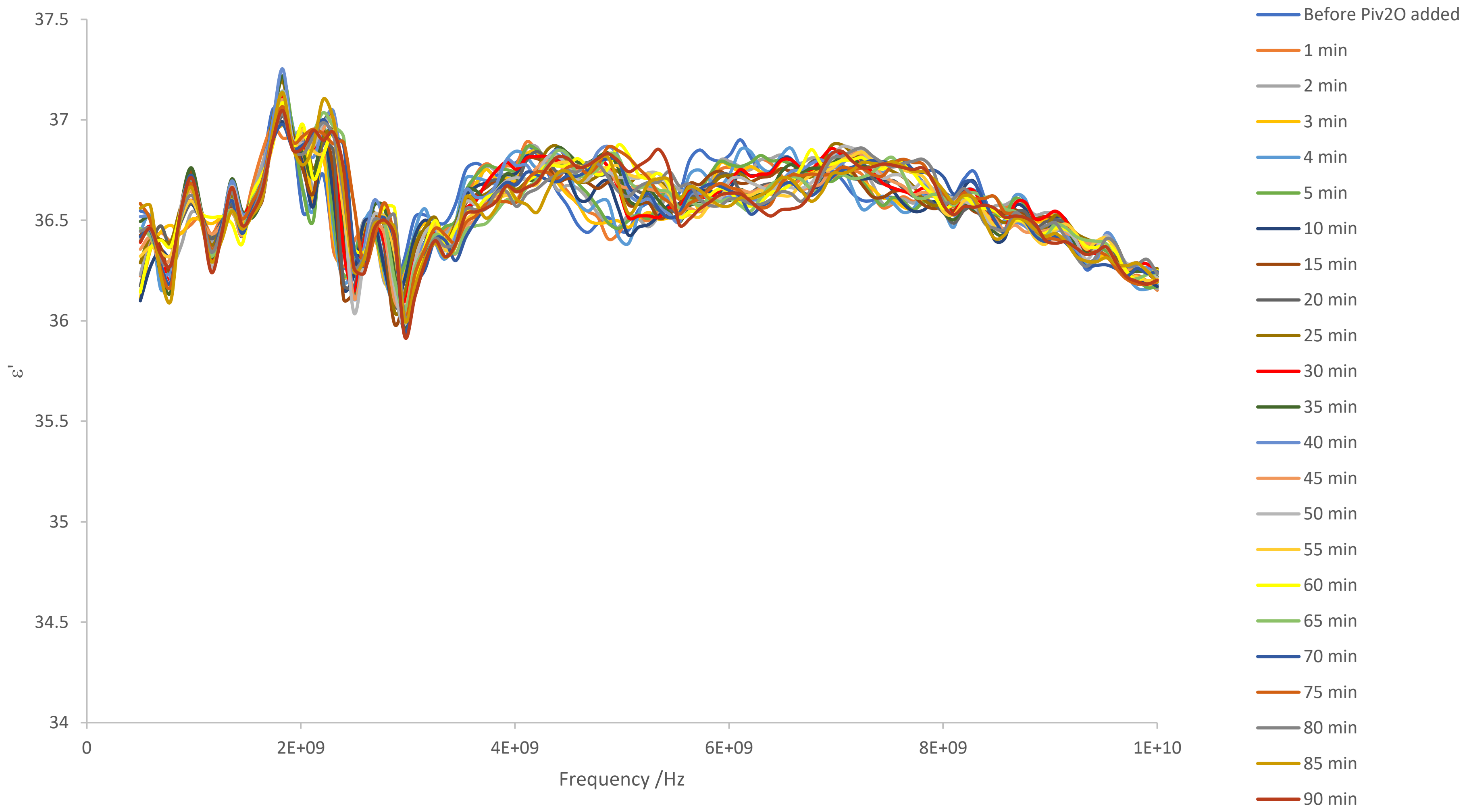

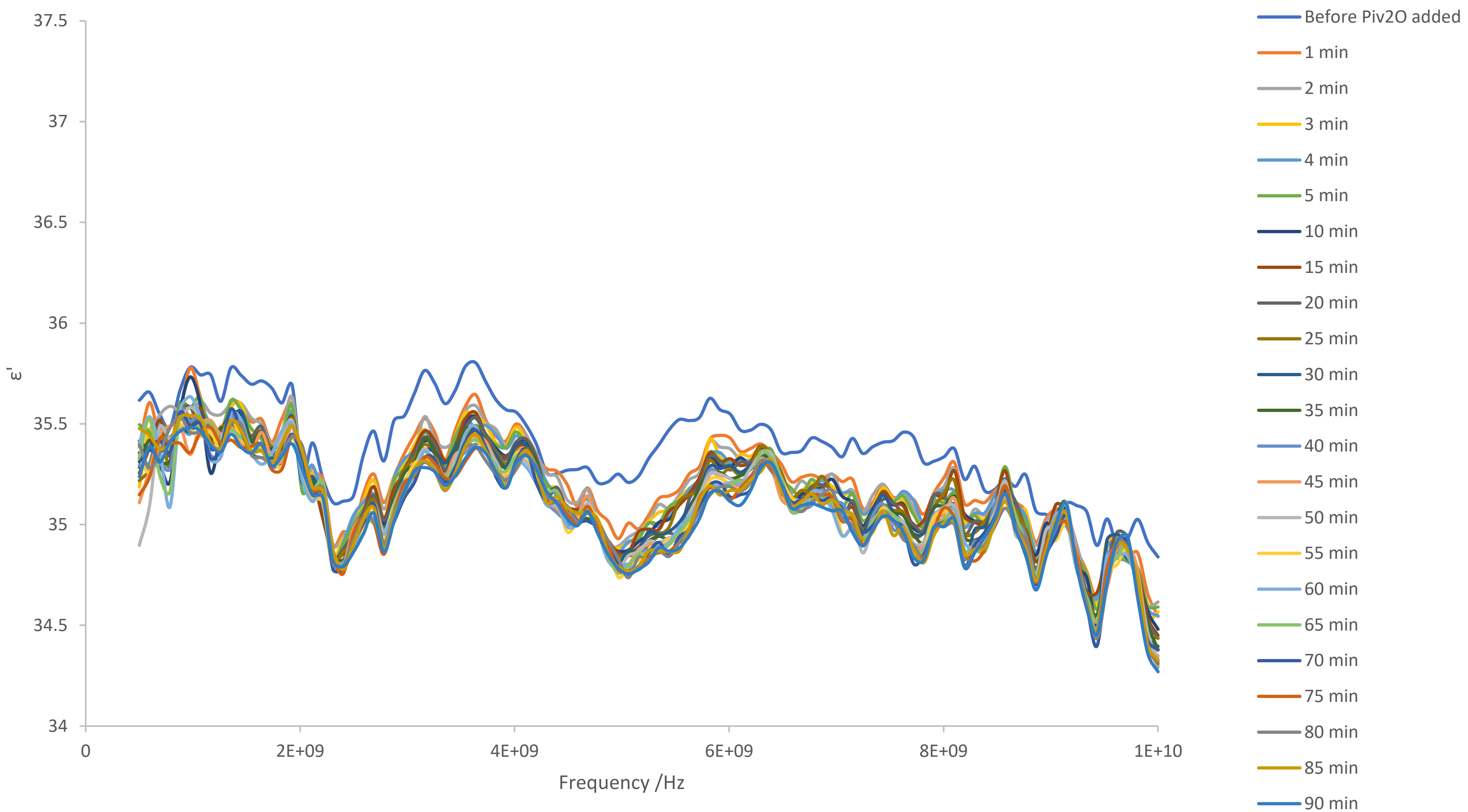

37.5
37
36.5
36
35.5
35
34.5
34
ε'
0
2E+09
4E+09
6E+09
8E+09
1E+10
Frequency /Hz
Before Piv2O added
1 min
2 min
3 min
4 min
5 min
10 min
15 min
20 min
25 min
30 min
35 min
40 min
45 min
50 min
55 min
60 min
65 min
70 min
75 min
80 min
85 min
90 min

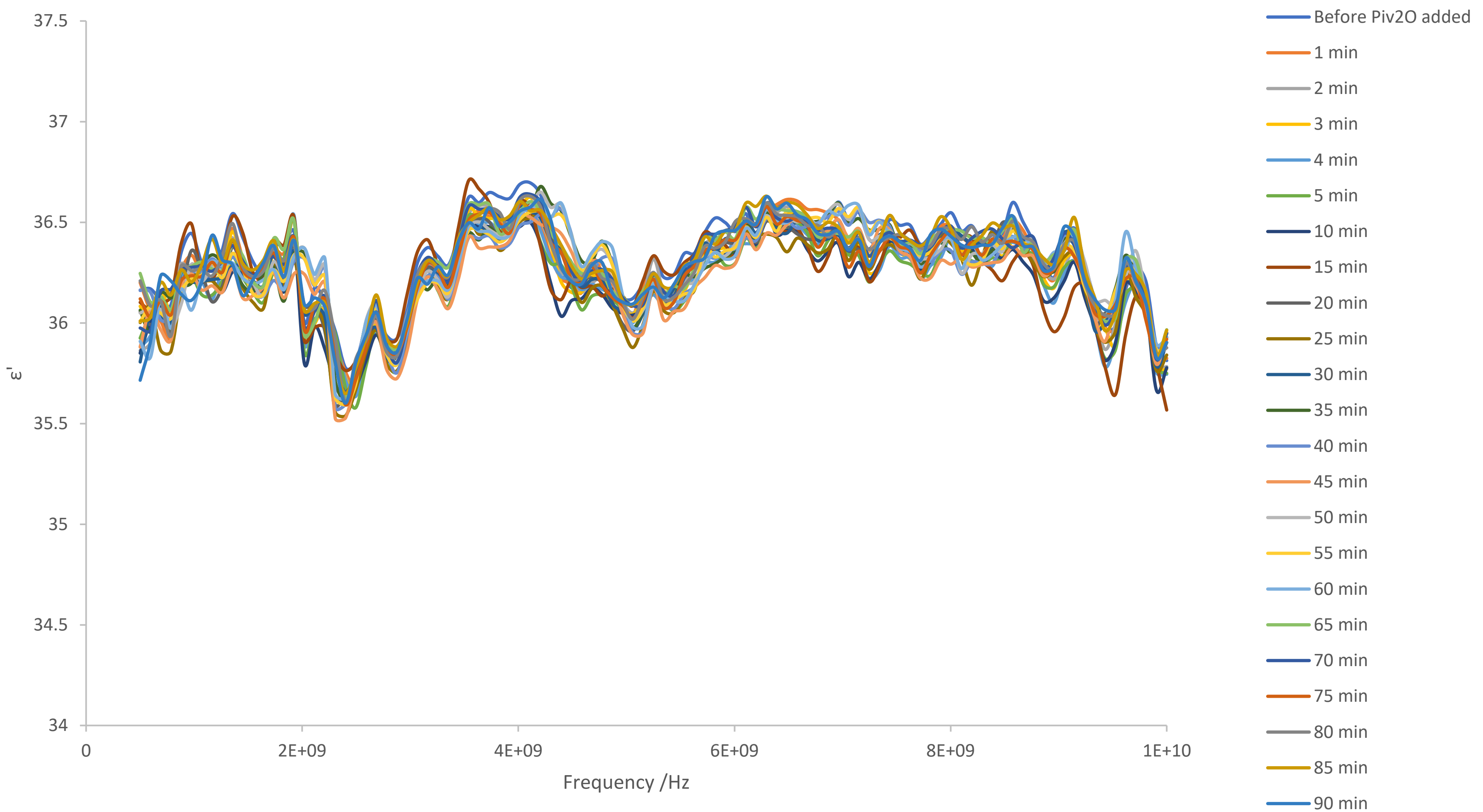

37.5
37
36.5
36
35.5
35
34.5
34
ε'
0
2E+09
4E+09
6E+09
8E+09
1E+10
Frequency /Hz
Before Piv2O added
1 min
2 min
3 min
4 min
5 min
10 min
15 min
20 min
25 min
30 min
35 min
40 min
45 min
50 min
55 min
60 min
65 min
70 min
75 min
80 min
85 min
90 min

S34

Plots of Dielectric Loss (ε'') *vs* Frequency

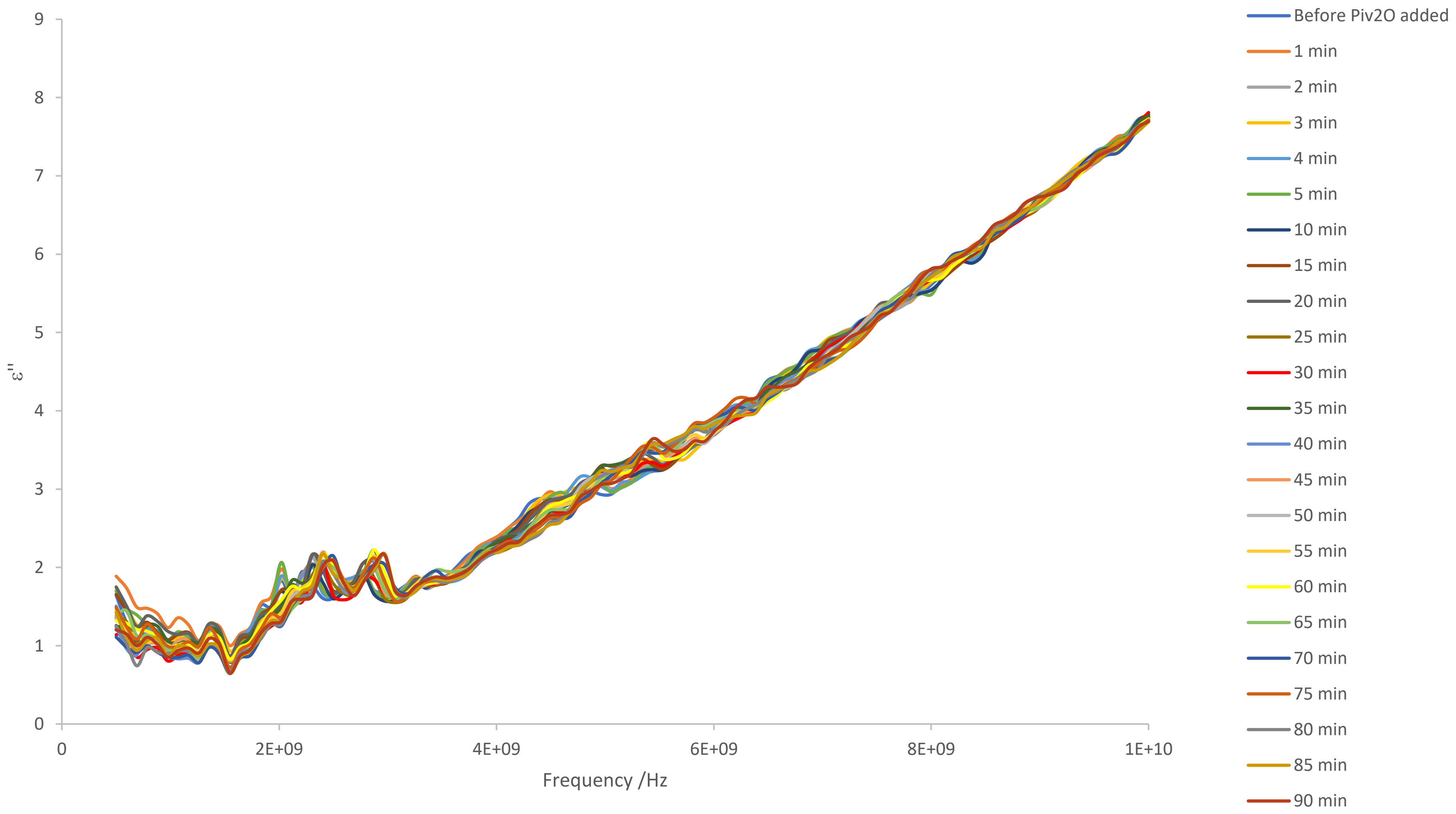

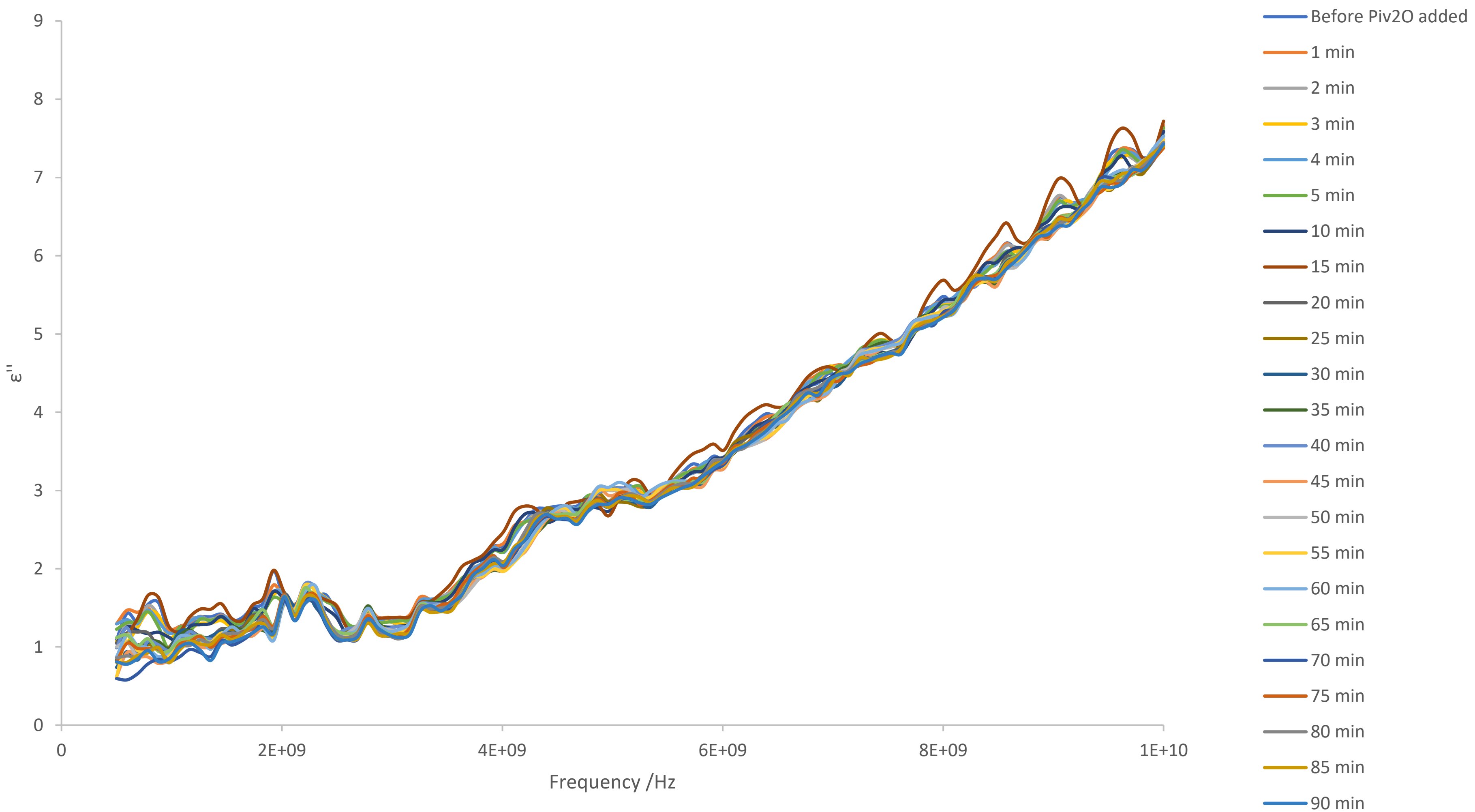

ε''
9
8
7
6
5
4
3
2
1
0
0
2E+09
4E+09
6E+09
8E+09
1E+10
Frequency /Hz
Before Piv2O added
1 min
2 min
3 min
4 min
5 min
10 min
15 min
20 min
25 min
30 min
35 min
40 min
45 min
50 min
55 min
60 min
65 min
70 min
75 min
80 min
85 min
90 min

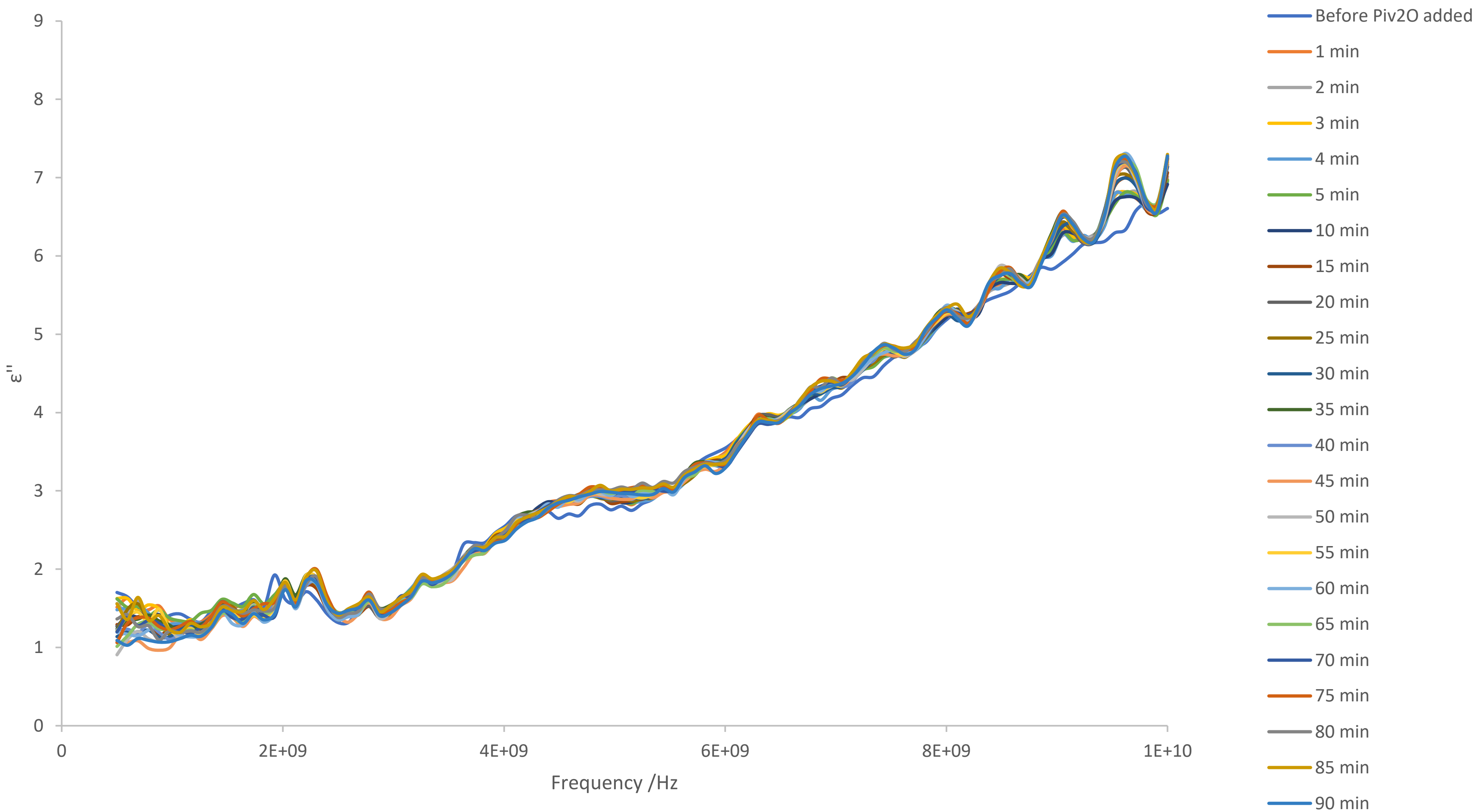

9
8
7
6
5
4
3
2
1
0
ε''
0
2E+09
4E+09
6E+09
8E+09
1E+10
Frequency /Hz
Before Piv2O added
1 min
2 min
3 min
4 min
5 min
10 min
15 min
20 min
25 min
30 min
35 min
40 min
45 min
50 min
55 min
60 min
65 min
70 min
75 min
80 min
85 min
90 min

Concentration-Time Plots Measured by ^{1}H NMR Spectroscopy

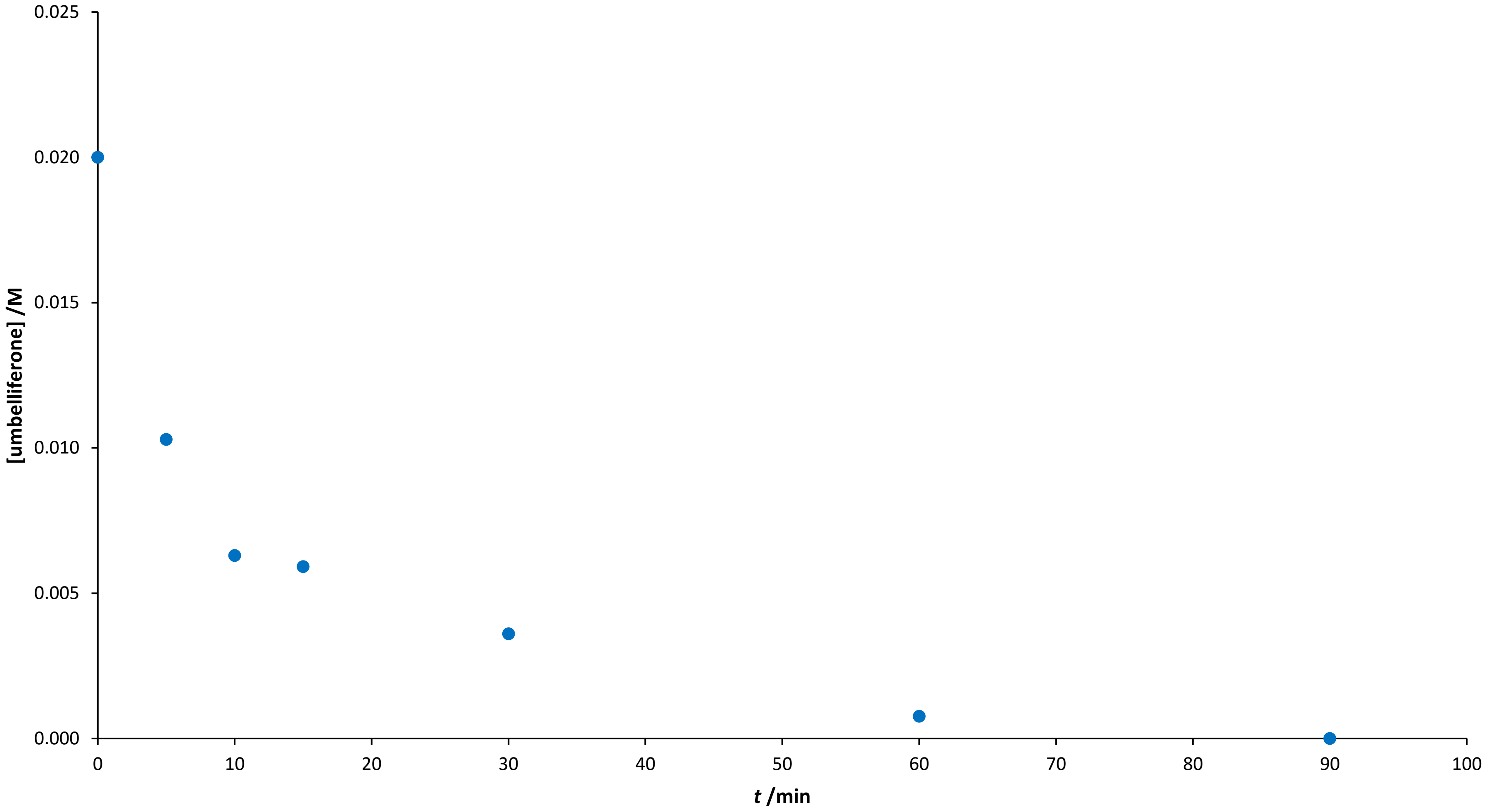

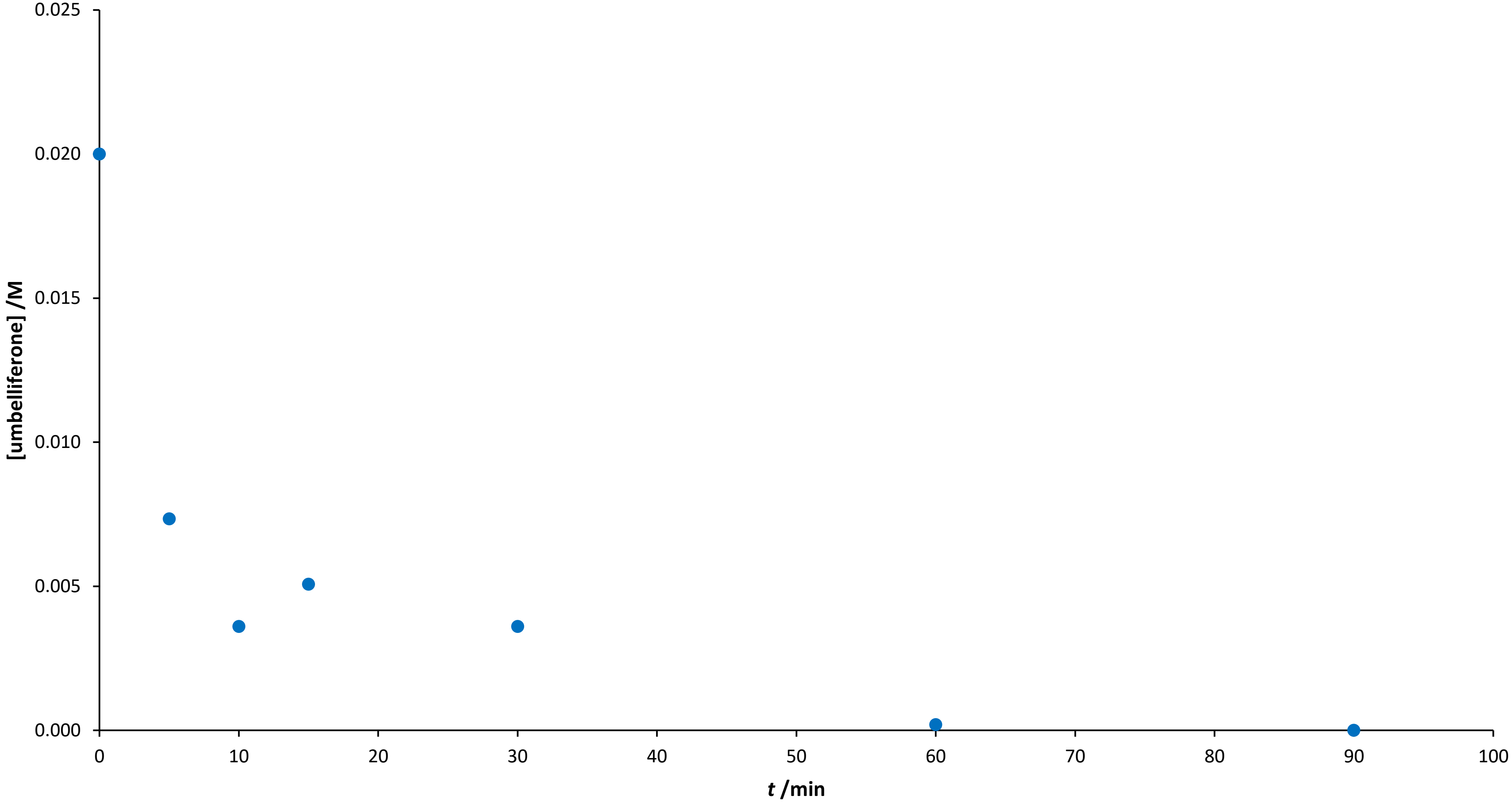

0.025
0.020
0.015
0.010
0.005
0.000
[umbelliferone] /M
0
10
20
30
40
50
60
70
80
90
100
t /min

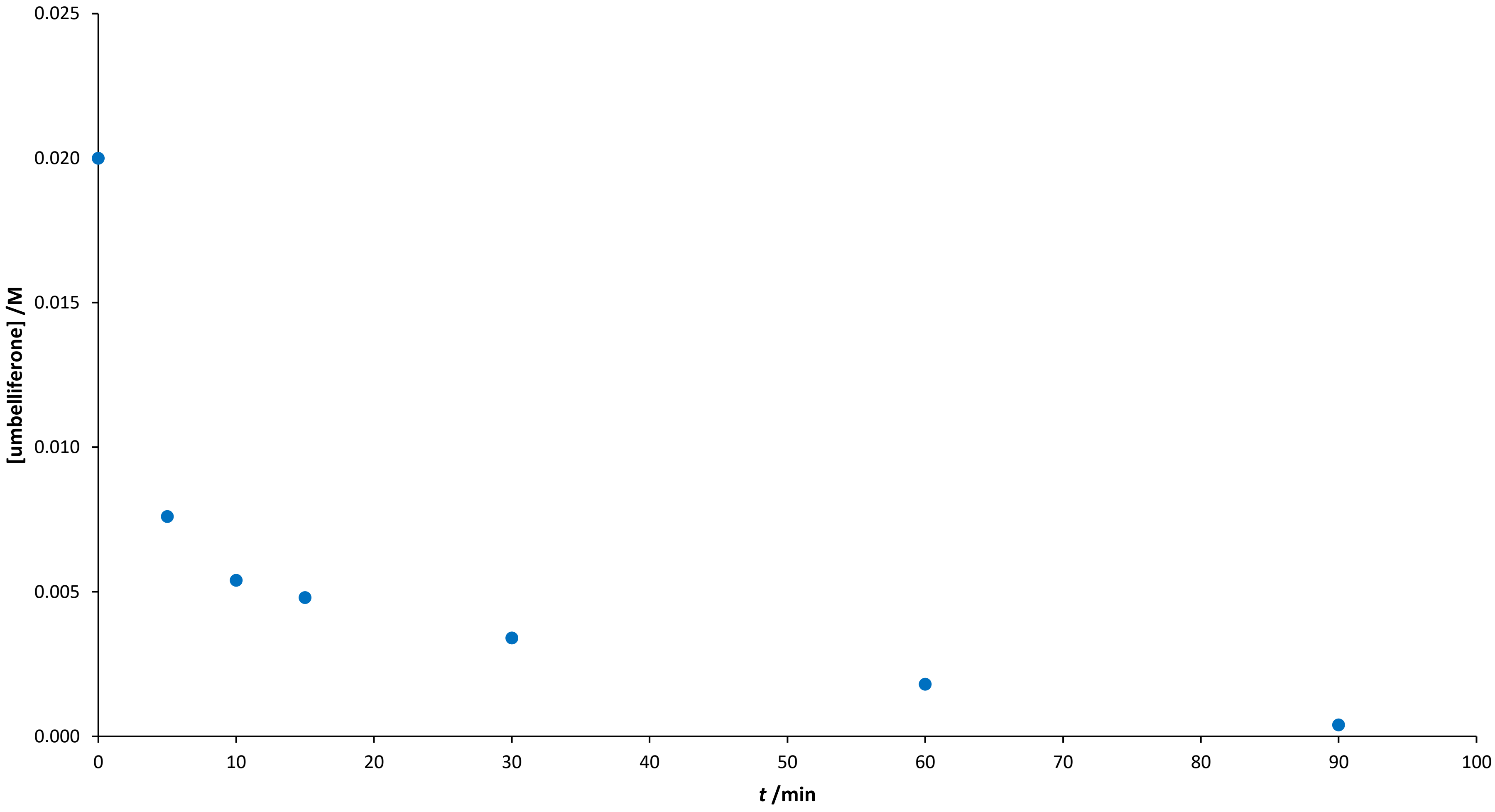

0.025
0.020
0.015
0.010
0.005
0.000
[umbelliferone] /M
0
10
20
30
40
50
60
70
80
90
100
t /min

PLS Model and Summary Statistics

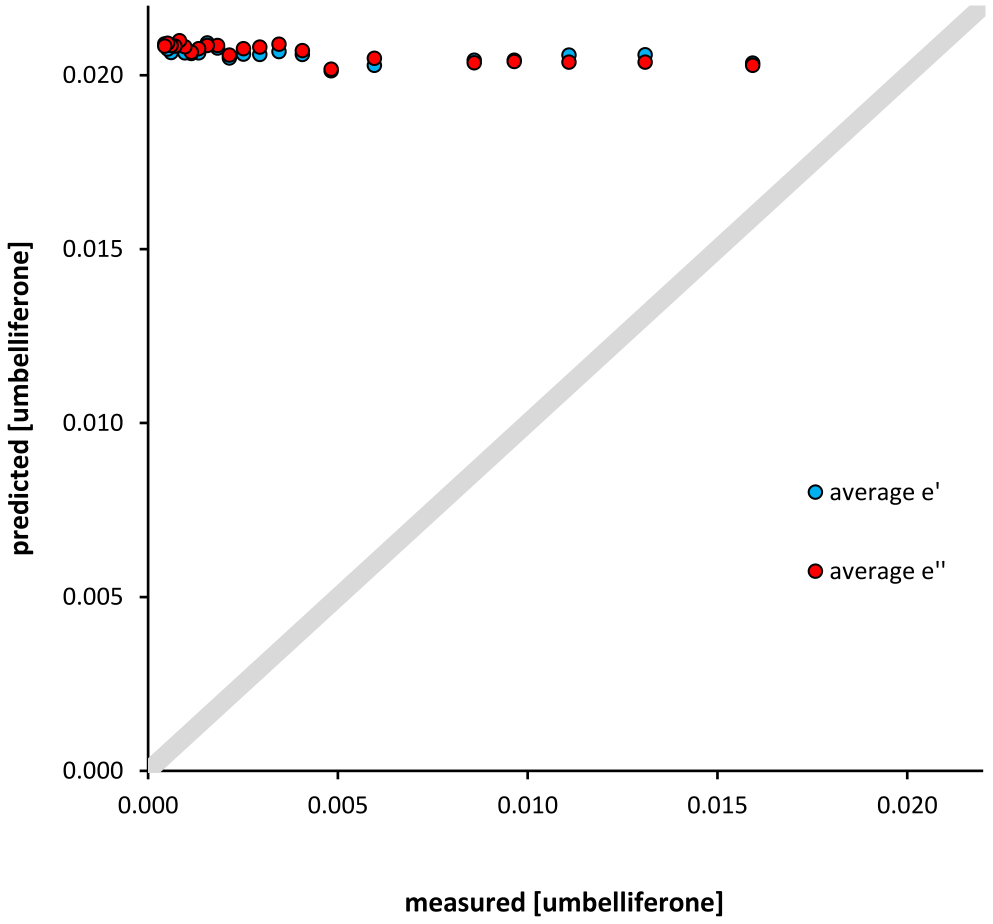

For dielectric constant model:

RMSEC = 0.000285

RMSECV = 0.000367

RMSEP = 0.016585 ± 0.000154 std dev

For dielectric loss model:

RMSEC = 0.000429

RMSECV = 0.000573

RMSEP = 0.016661 ± 0.016661 std dev

Gray line represents the ideal correlation between measured and predicted values ($x = y$).

8. Data From Manuscript Figure 8

Plots of Dielectric Constant (ε’) *vs* Frequency

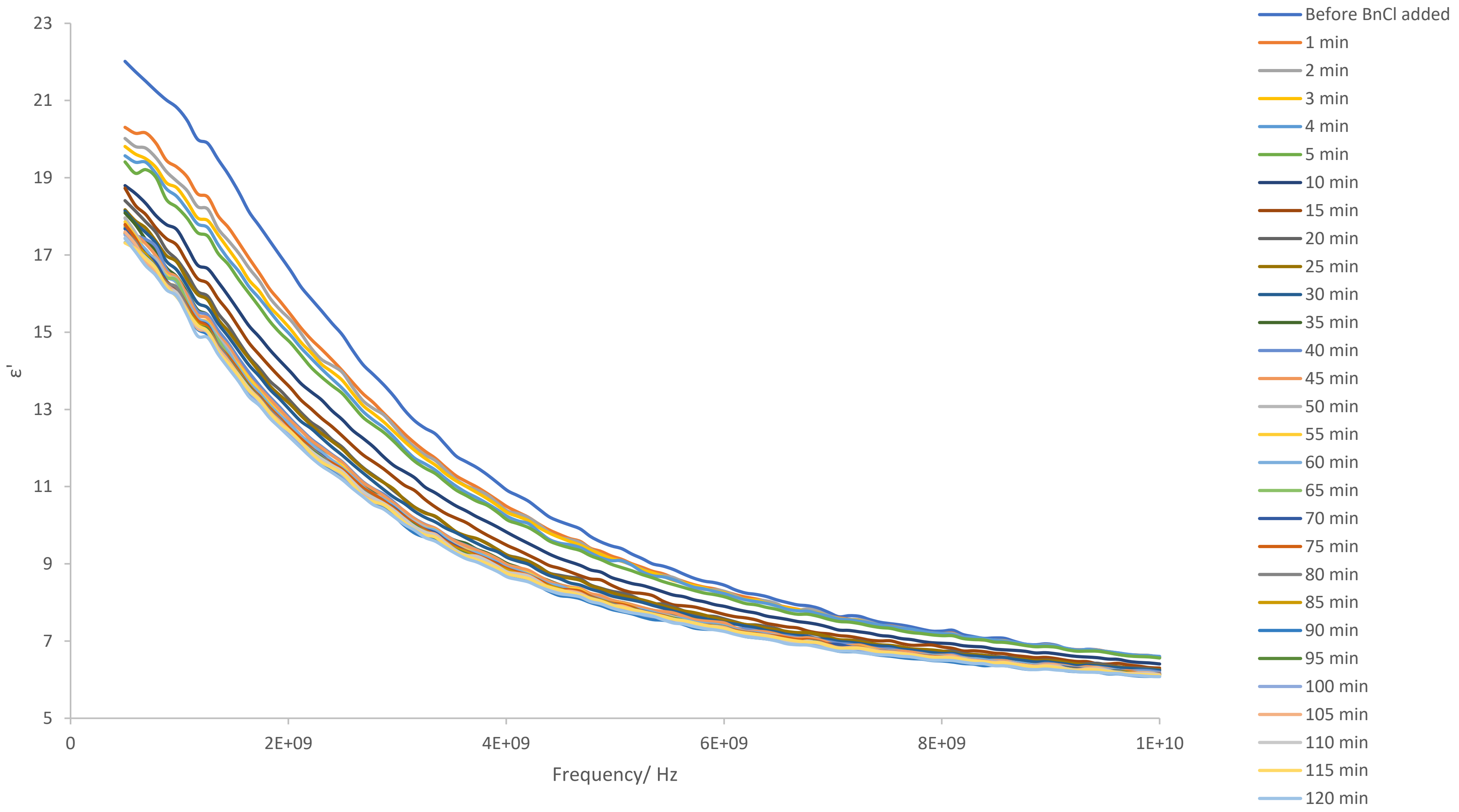

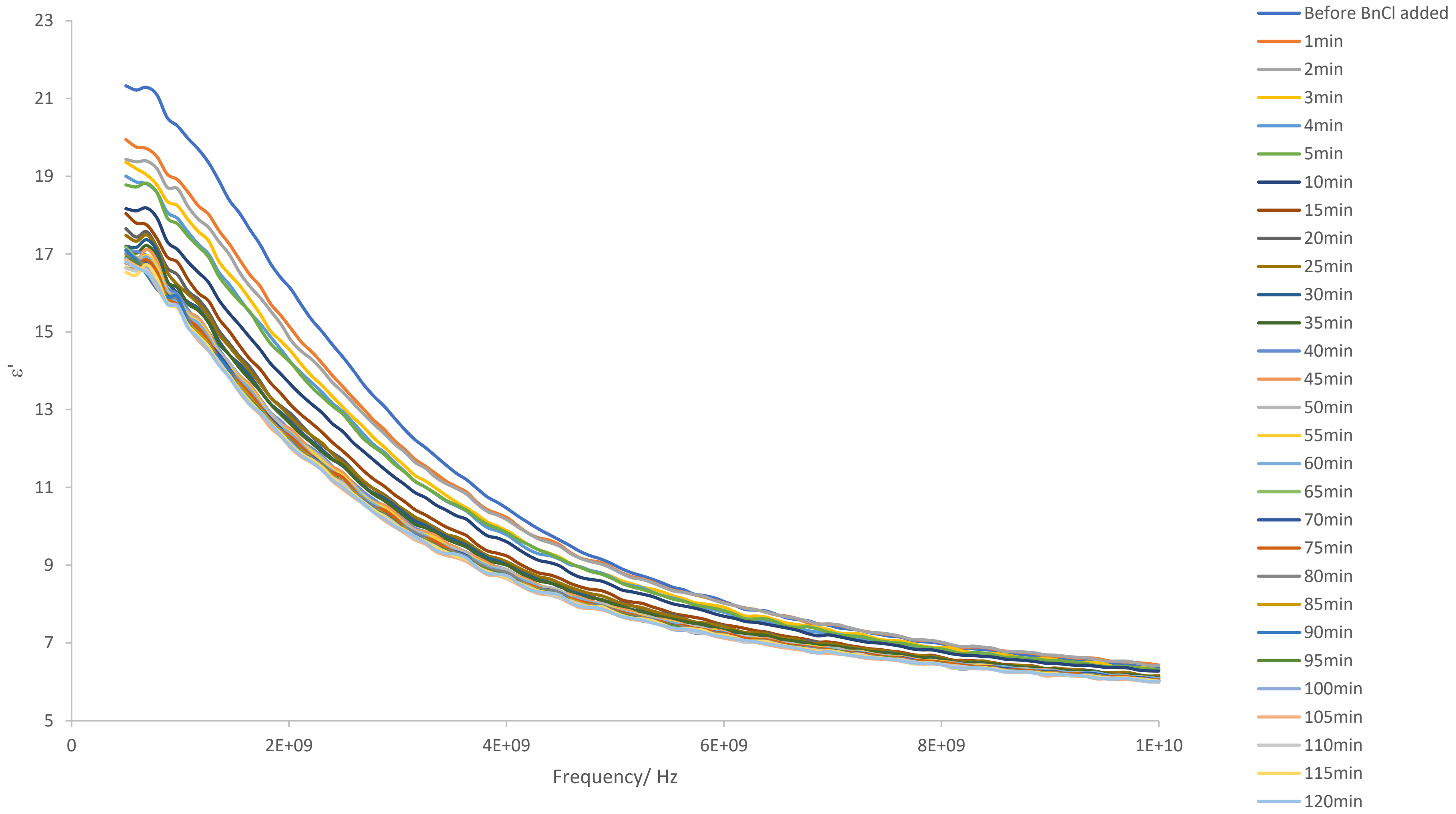

23
21
19
17
15
13
11
9
7
5
ε'
0
2E+09
4E+09
6E+09
8E+09
1E+10
Frequency/ Hz
Before BnCl added
1min
2min
3min
4min
5min
10min
15min
20min
25min
30min
35min
40min
45min
50min
55min
60min
65min
70min
75min
80min
85min
90min
95min
100min
105min
110min
115min
120min

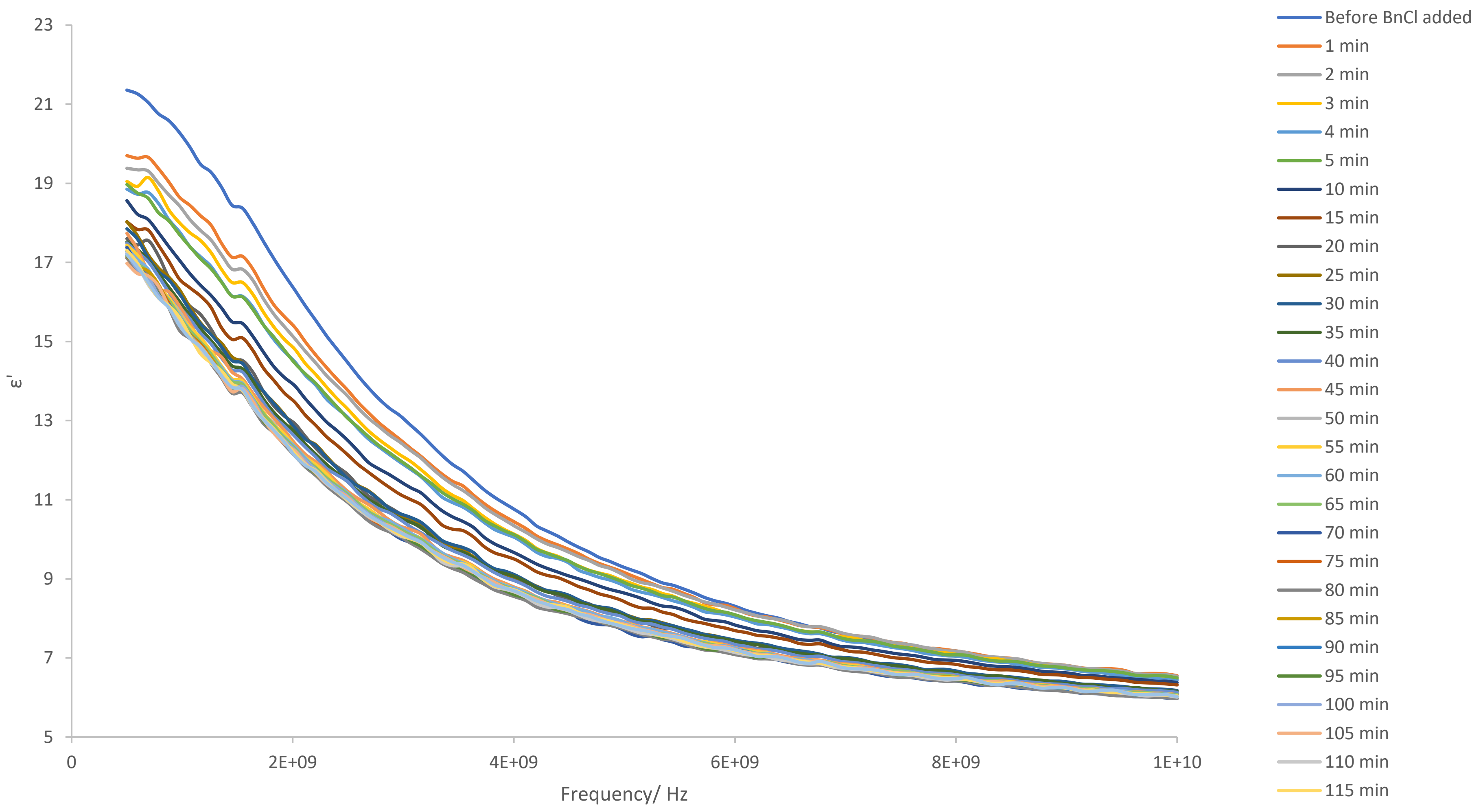

23
21
19
17
15
13
11
9
7
5
ε'
0
2E+09
4E+09
6E+09
8E+09
1E+10
Frequency/ Hz
Before BnCl added
1 min
2 min
3 min
4 min
5 min
10 min
15 min
20 min
25 min
30 min
35 min
40 min
45 min
50 min
55 min
60 min
65 min
70 min
75 min
80 min
85 min
90 min
95 min
100 min
105 min
110 min
115 min
120 min

Plots of Dielectric Loss (ε'') *vs* Frequency

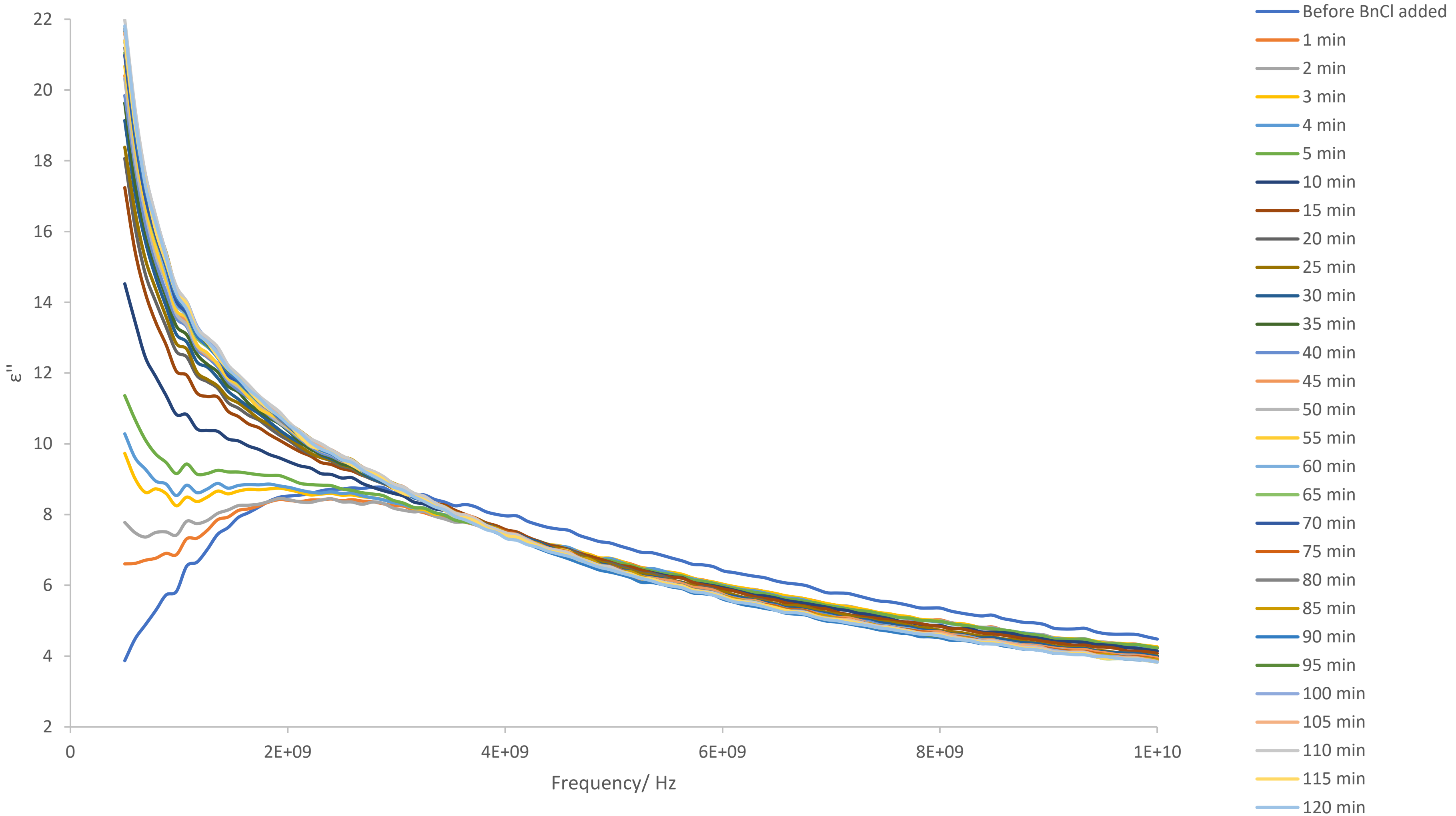

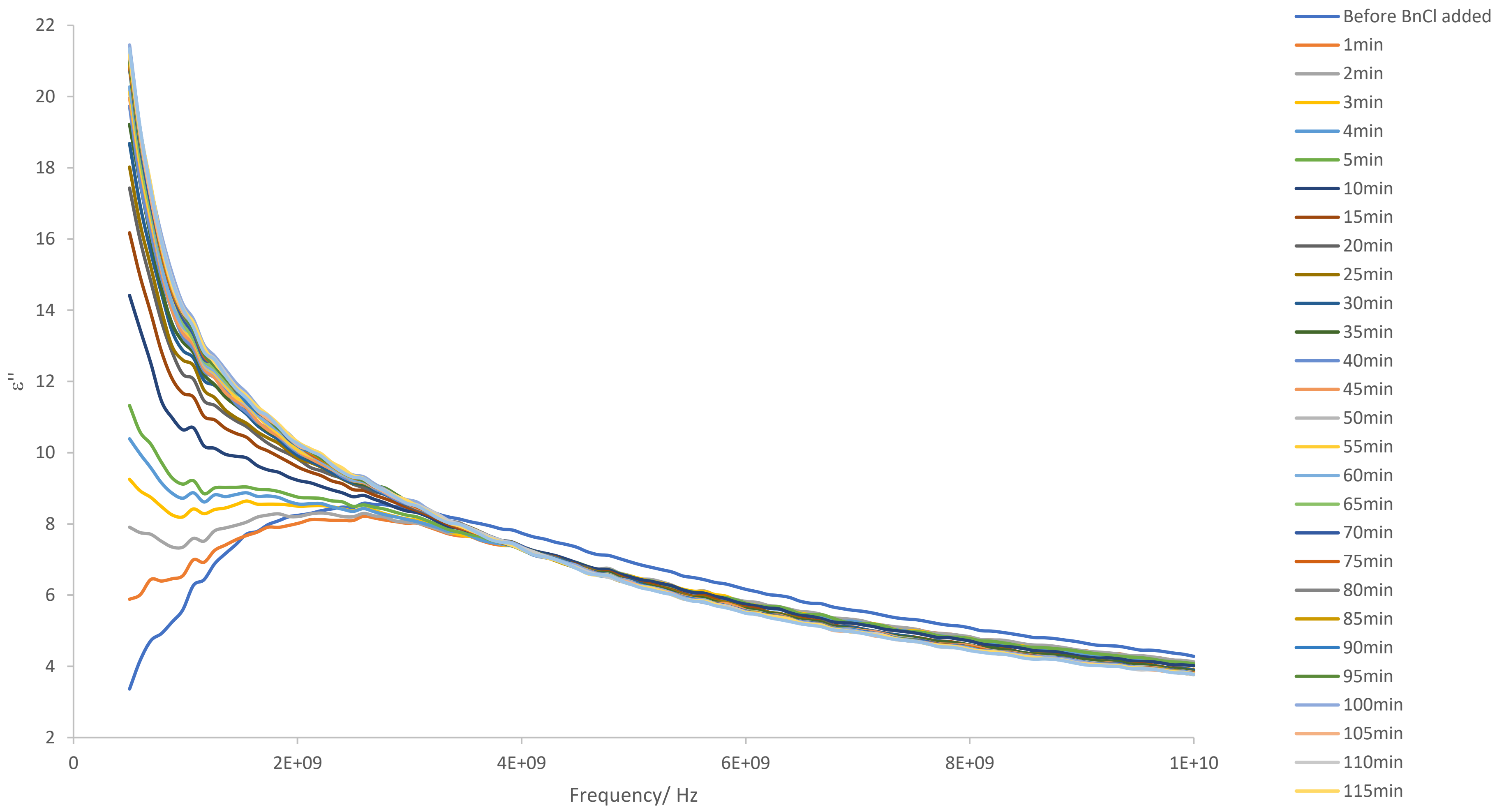
22
20
18
16
14
12
10
8
6
4
2
ε''
0
2E+09
4E+09
6E+09
8E+09
1E+10
Frequency/ Hz
Before BnCl added
1min
2min
3min
4min
5min
10min
15min
20min
25min
30min
35min
40min
45min
50min
55min
60min
65min
70min
75min
80min
85min
90min
95min
100min
105min
110min
115min
120min

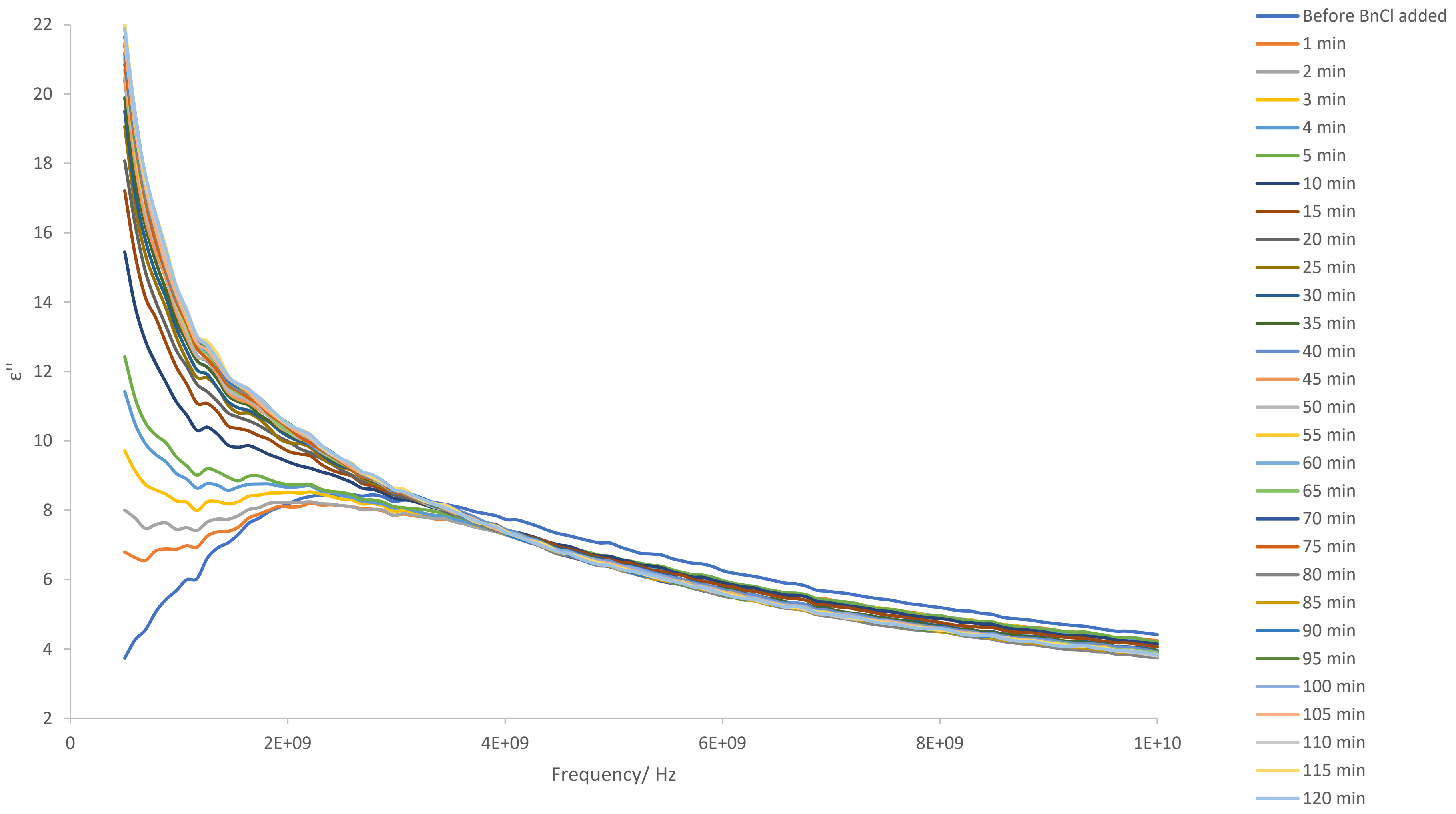

22
20
18
16
14
12
10
8
6
4
2
ε''
0
2E+09
4E+09
6E+09
8E+09
1E+10
Frequency/ Hz
Before BnCl added
1 min
2 min
3 min
4 min
5 min
10 min
15 min
20 min
25 min
30 min
35 min
40 min
45 min
50 min
55 min
60 min
65 min
70 min
75 min
80 min
85 min
90 min
95 min
100 min
105 min
110 min
115 min
120 min

Concentration-Time Plots Measured by ^{1}H NMR Spectroscopy

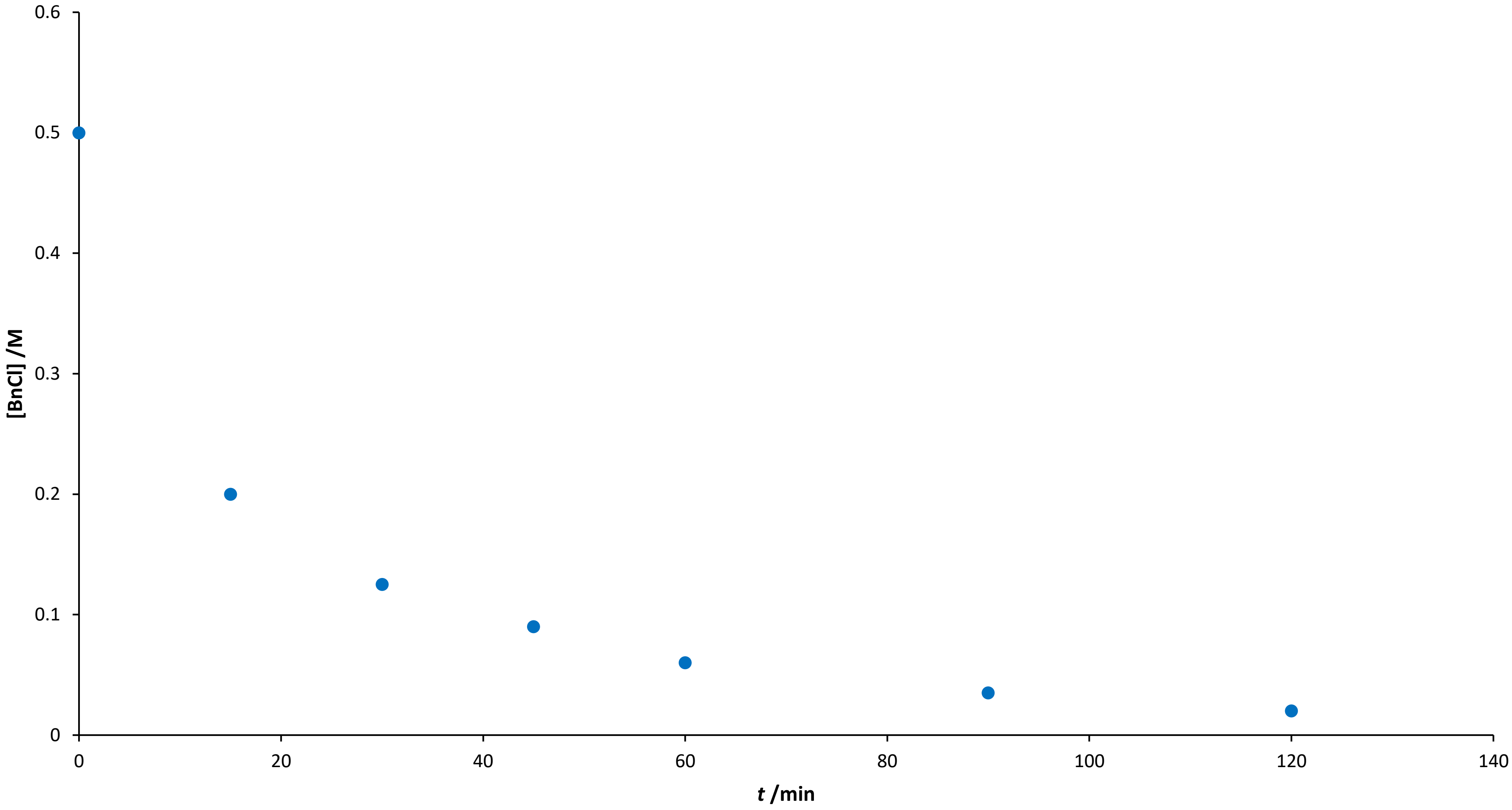

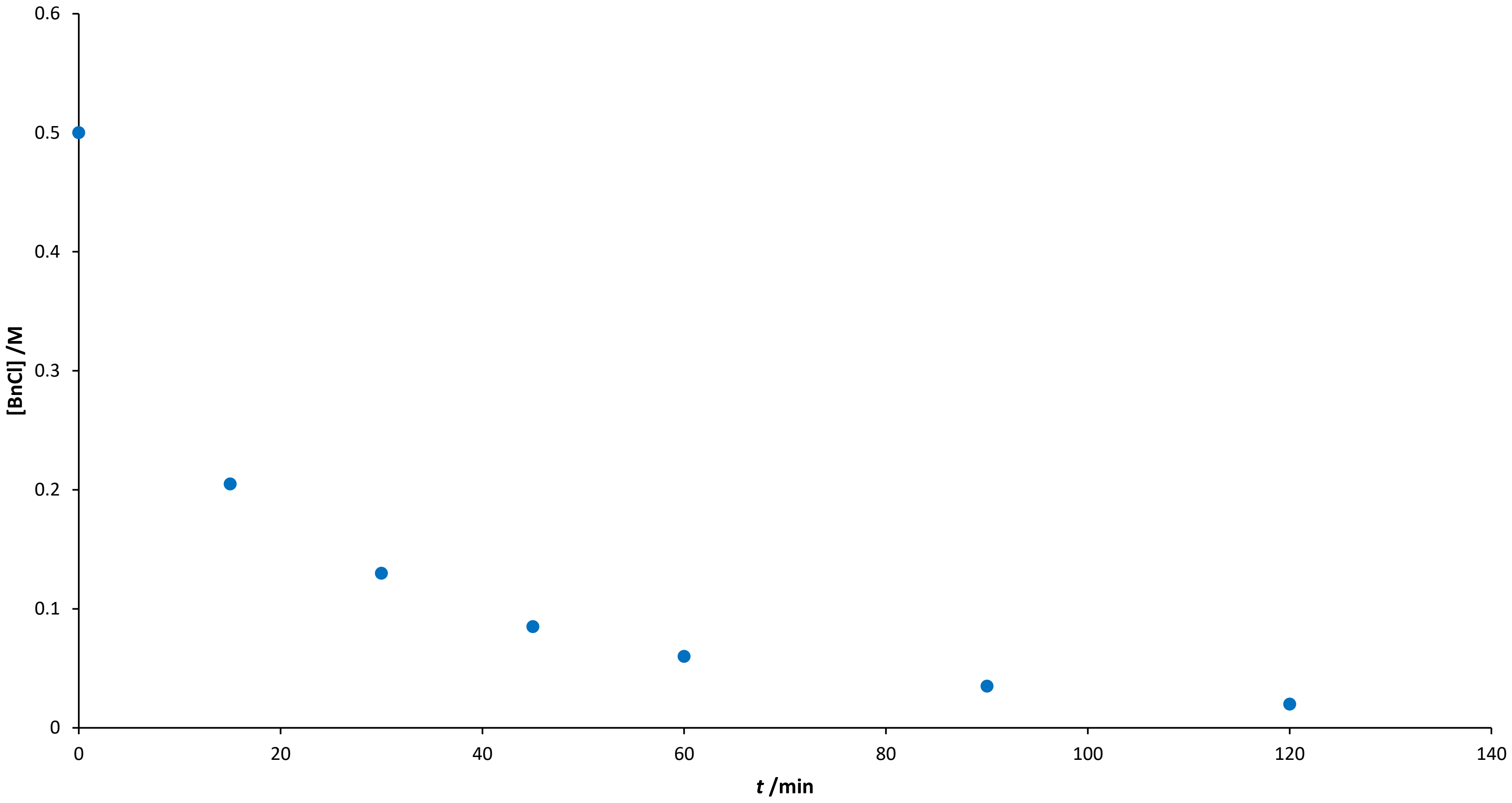

0.6
0.5
0.4
0.3
0.2
0.1
0
[BnCl] /M
0
20
40
60
80
100
120
140
t /min

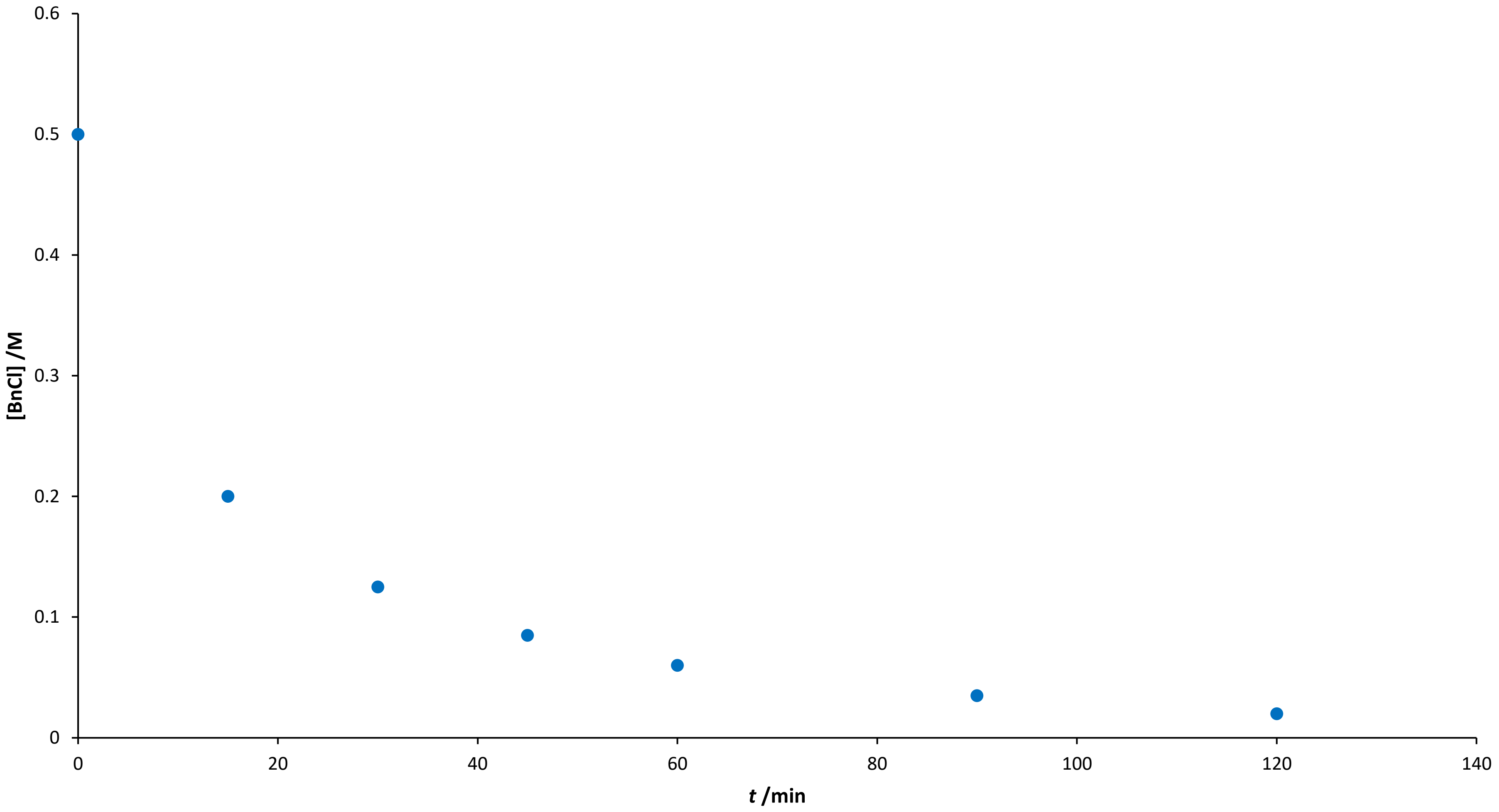

0.6
0.5
0.4
0.3
0.2
0.1
0
[BnCl] /M
0
20
40
60
80
100
120
140
t /min

PLS Model and Summary Statistics

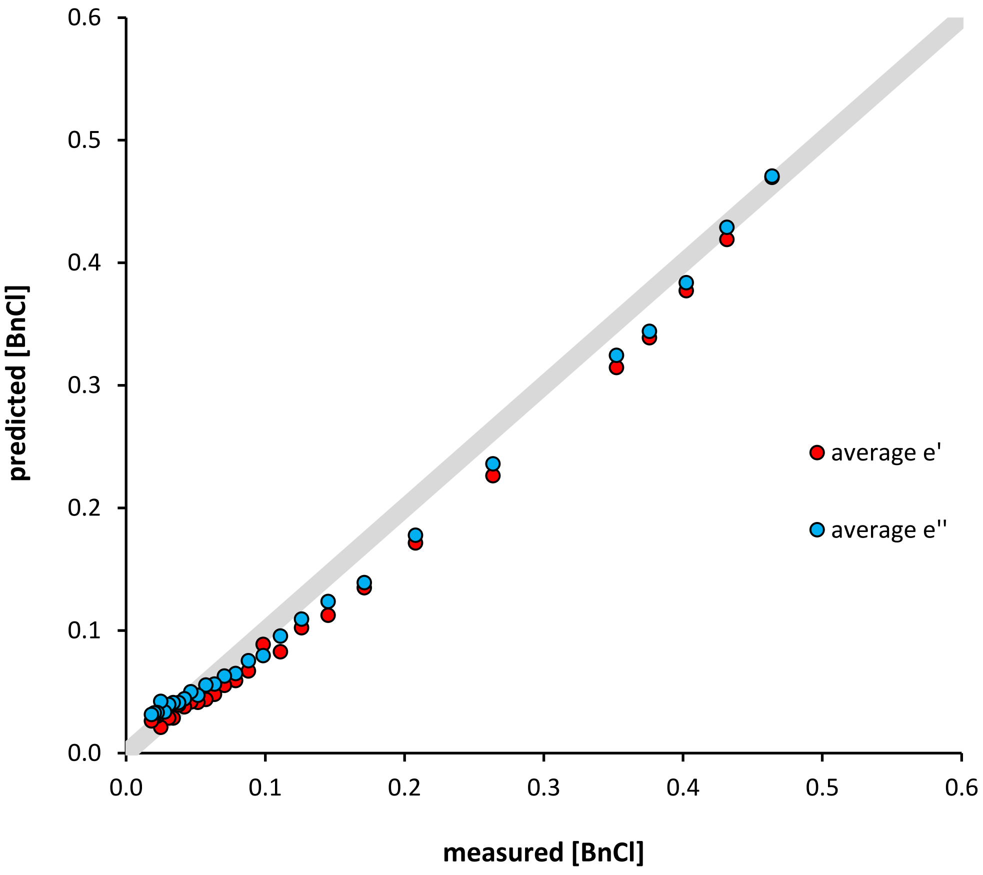

For dielectric constant model:

RMSEC = 0.000285

RMSECV = 0.000367

RMSEP = 0.016585 ± 0.000154 std dev

For dielectric loss model:

RMSEC = 0.000429

RMSECV = 0.000573

RMSEP = 0.016661 ± 0.016661 std dev

Gray line represents the ideal correlation between measured and predicted values (*x* = *y*).

9. NMR Spectra of Isolated Compounds

3 – ^{1}H NMR (400 MHz, $CDCl_3$):

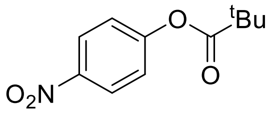

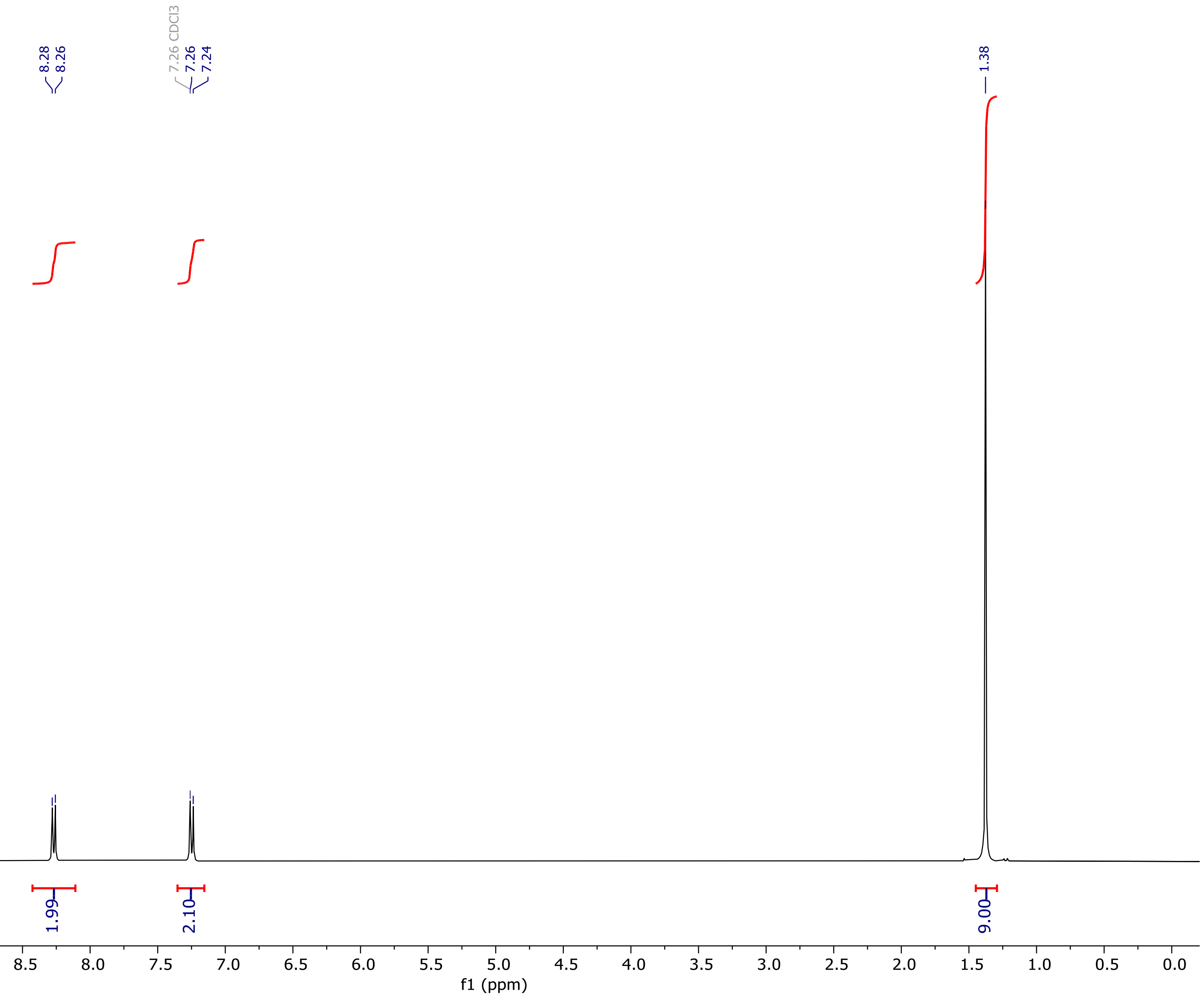

3 – $^{13}C\{^{1}H\}$ NMR (100 MHz, $CDCl_3$):

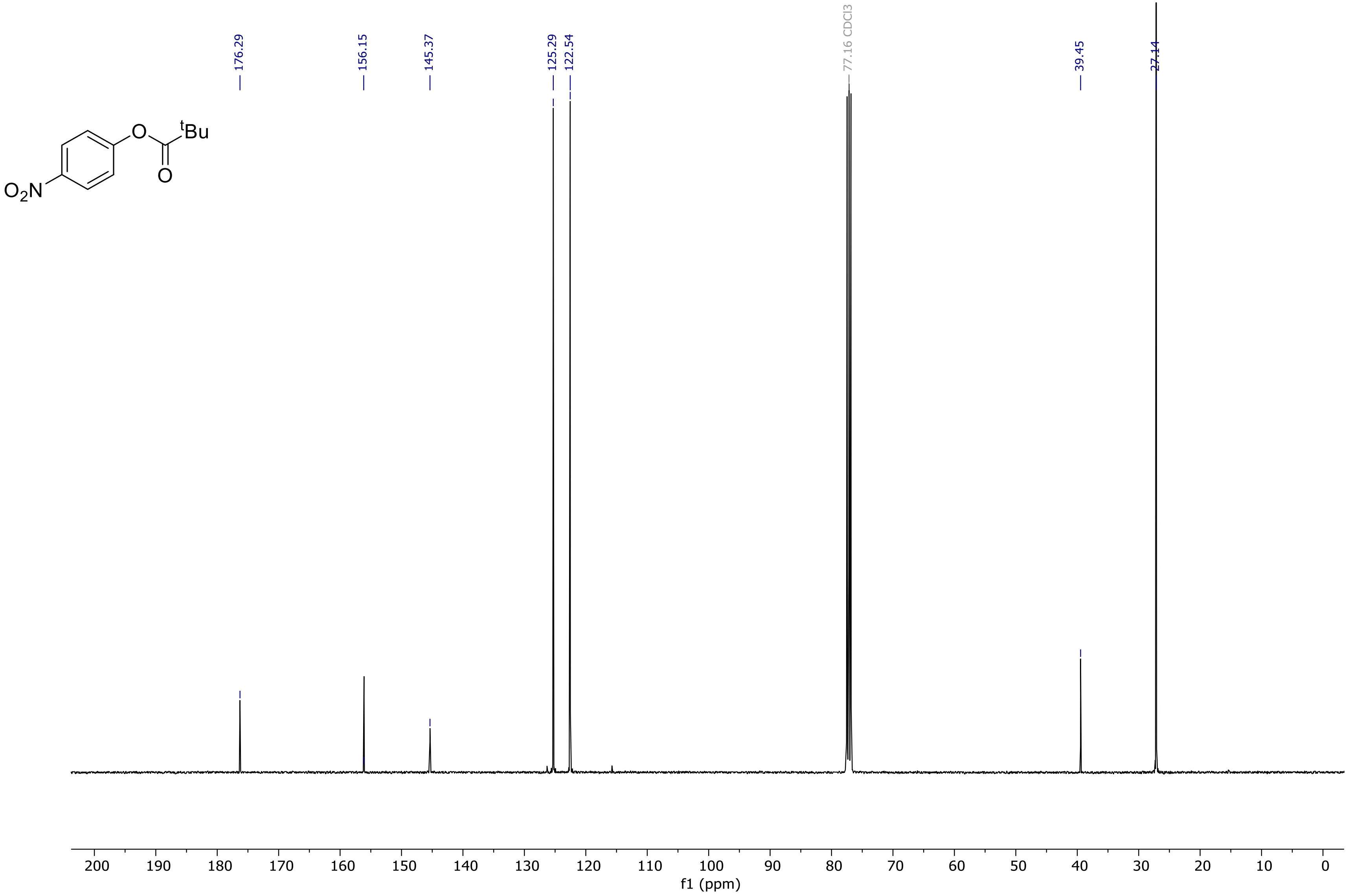

6 – ^{1}H NMR (400 MHz, $CDCl_3$):

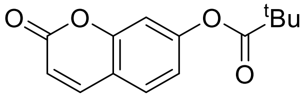

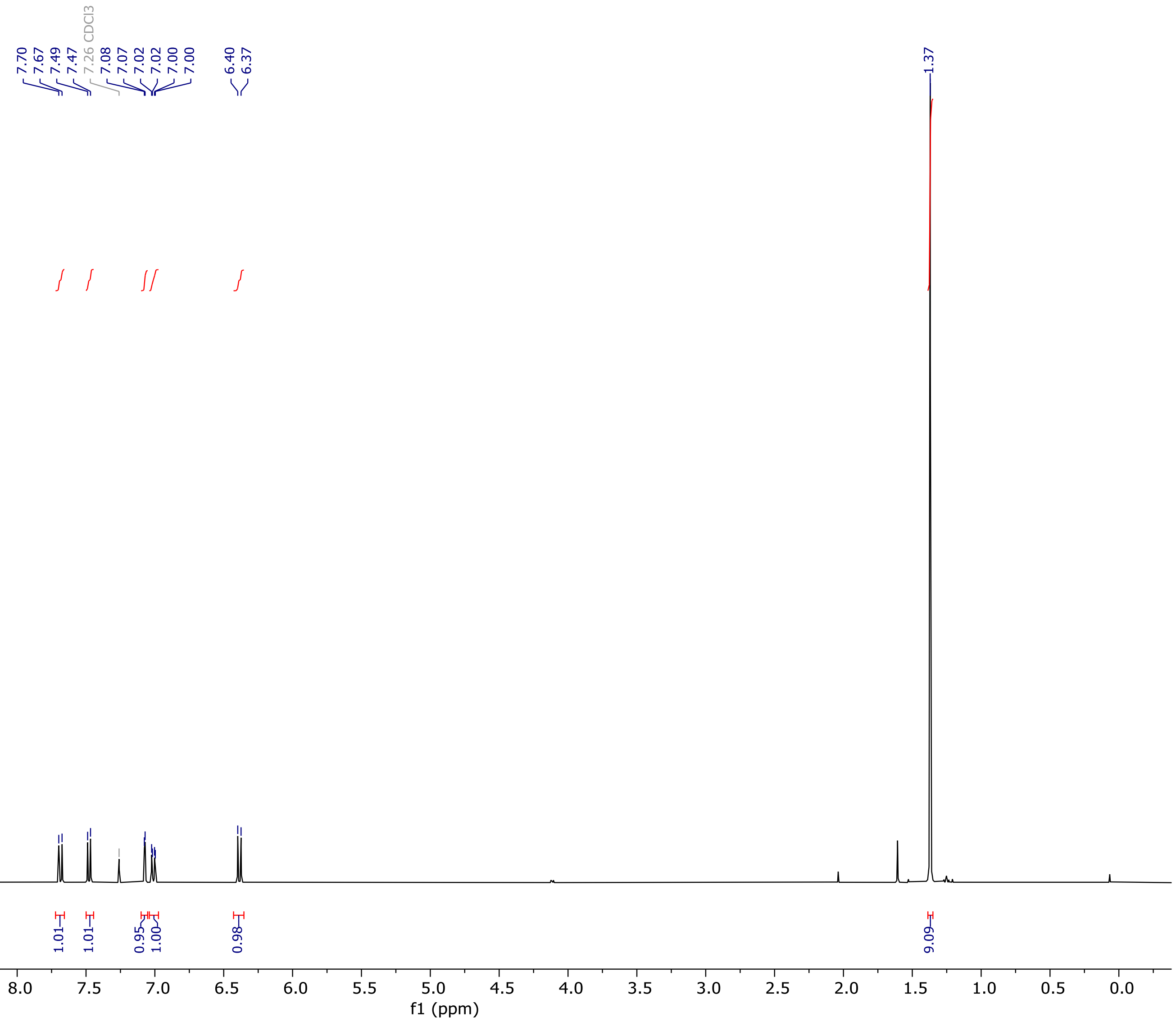

6 – $^{13}C\{^{1}H\}$ NMR (100 MHz, $CDCl_3$):

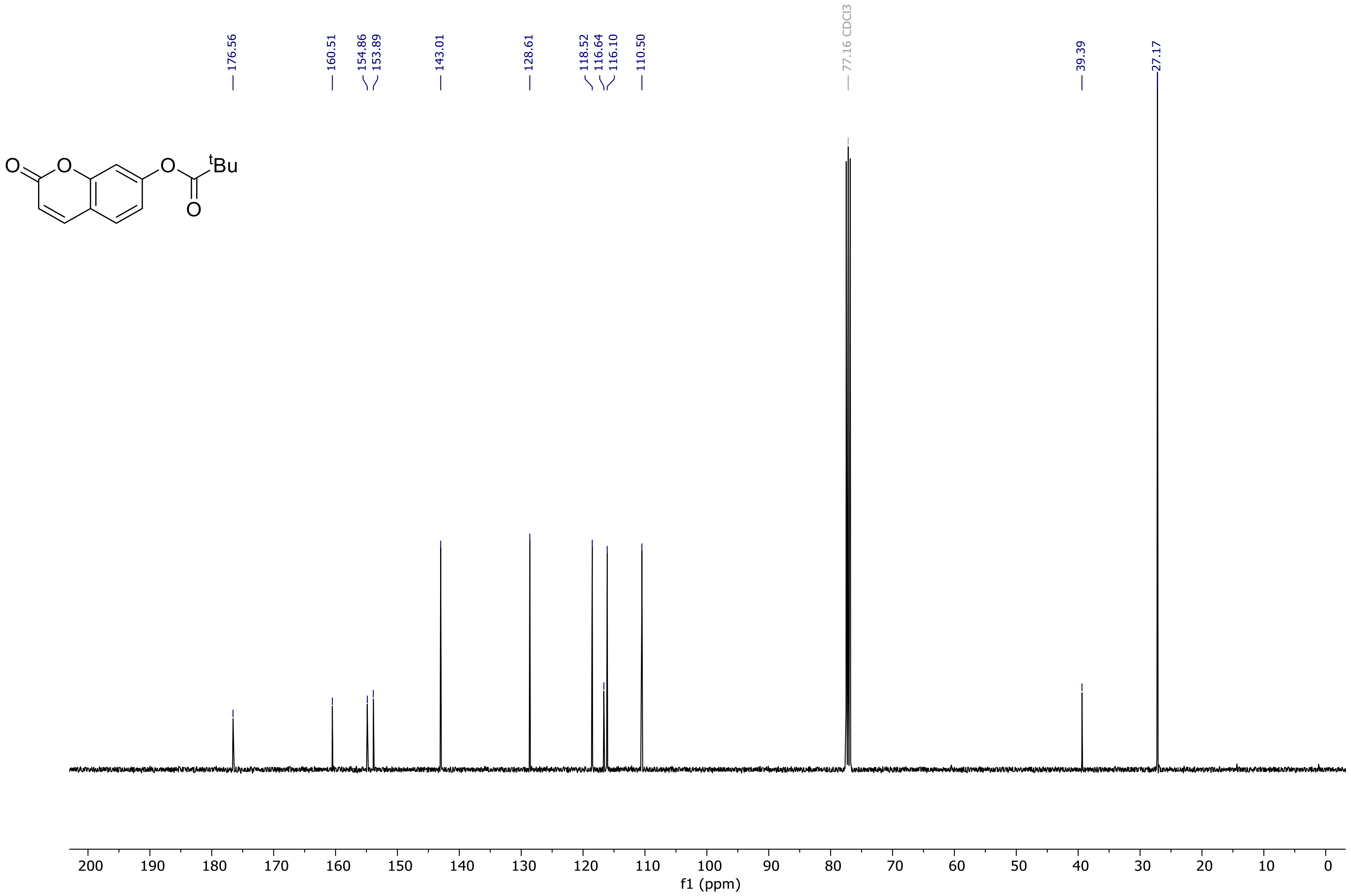

9 – ^{1}H NMR (400 MHz, DMSO-d_6):

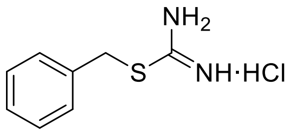

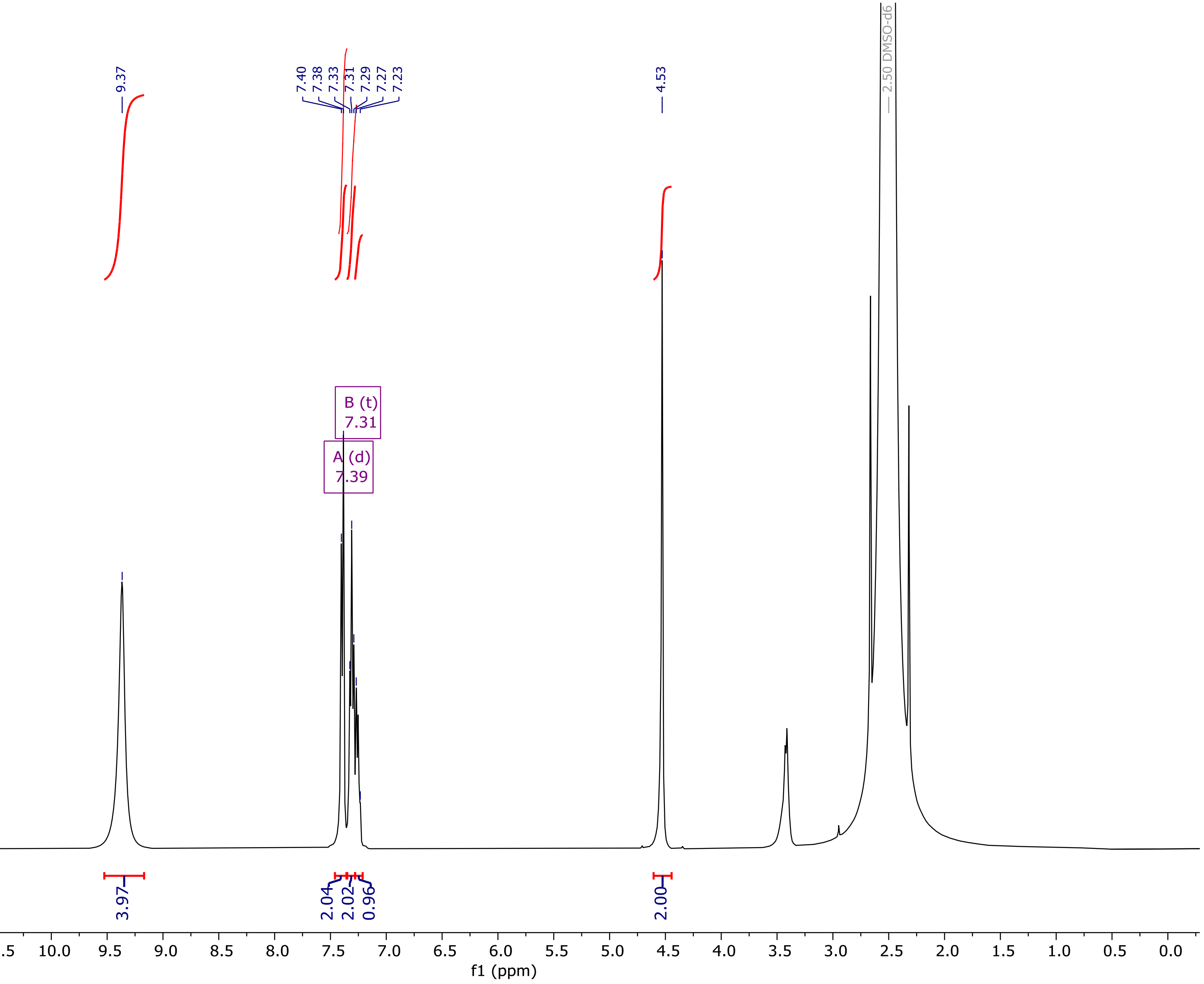

9 – $^{13}C\{^1H\}$ NMR (100 MHz, DMSO-d_6):

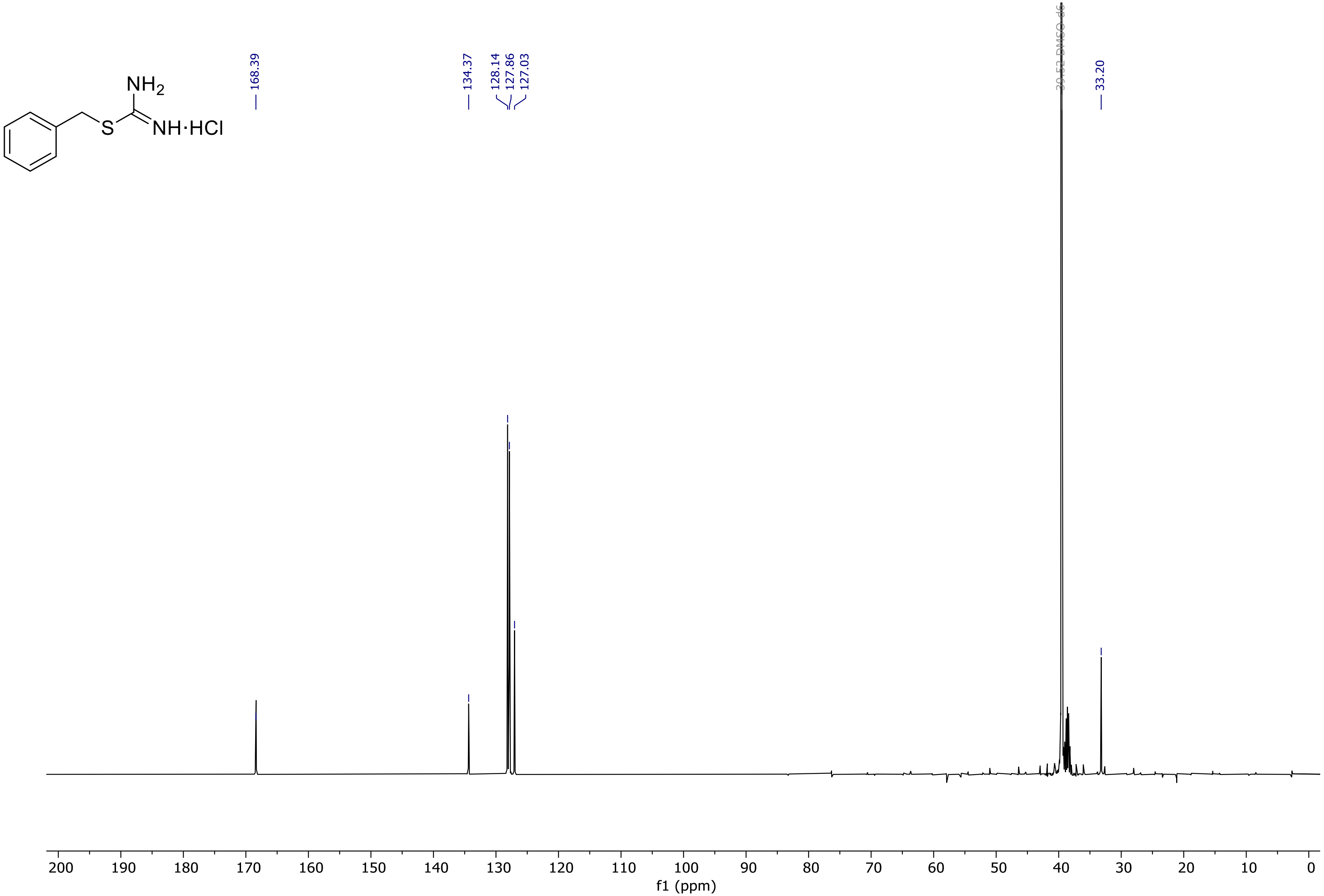

Supplement: Supplementary file 1 — op3c00081_si_001.pdf [file op3c00081_si_001.pdf]
